# Characterizing the omics landscape based on 10,000+ datasets

**DOI:** 10.1038/s41598-025-87256-5

**Published:** 2025-01-25

**Authors:** Eva Brombacher, Oliver Schilling, Clemens Kreutz

**Affiliations:** 1 Institute of Medical Biometry and Statistics, Faculty of Medicine and Medical Center-University of Freiburg, Freiburg, Germany; 2 Centre for Integrative Biological Signaling Studies (CIBSS), University of Freiburg, Freiburg, Germany; 3 Spemann Graduate School of Biology and Medicine (SGBM), University of Freiburg, Freiburg, Germany; 4 Faculty of Biology, University of Freiburg, Freiburg, Germany; 5 Institute for Surgical Pathology, Medical Center-University of Freiburg, Faculty of Medicine, University of Freiburg, Freiburg, Germany; 6 German Cancer Consortium (DKTK) and German Cancer Research Center (DKFZ), Heidelberg, Germany; 7 BIOSS Centre for Biological Signaling Studies, University of Freiburg, Freiburg, Germany

**Keywords:** Bioinformatics, Data acquisition, Data processing, Biological techniques

## Abstract

The characteristics of data produced by omics technologies are pivotal, as they critically influence the feasibility and effectiveness of computational methods applied in downstream analyses, such as data harmonization and differential abundance analyses. Furthermore, variability in these data characteristics across datasets plays a crucial role, leading to diverging outcomes in benchmarking studies, which are essential for guiding the selection of appropriate analysis methods in all omics fields. Additionally, downstream analysis tools are often developed and applied within specific omics communities due to the presumed differences in data characteristics attributed to each omics technology. In this study, we investigate over ten thousand datasets to understand how proteomics, metabolomics, lipidomics, transcriptomics, and microbiome data vary in specific data characteristics. We were able to show patterns of data characteristics specific to the investigated omics types and provide a tool that enables researchers to assess how representative a given omics dataset is for its respective discipline. Moreover, we illustrate how data characteristics can impact analyses at the example of normalization in the presence of sample-dependent proportions of missing values. Given the variability of omics data characteristics, we encourage the systematic inspection of these characteristics in benchmark studies and for downstream analyses to prevent suboptimal method selection and unintended bias.

## Introduction

Data characteristics impact algorithm performance and, consequently, the outcome of benchmarking studies. Researchers must account for these characteristics when selecting analysis methods and interpreting benchmark results. The general concept that there is no one-fits-all data processing strategy^1^is also applicable for biological high-throughput (omics) data. Thus, rather than focusing on the best method in general, performance should be evaluated in relation to the dataset characteristics that influence method performance, as these characteristics play a key role^1^. Previous publications emphasize the importance of selecting appropriate benchmark datasets^2^. To make meaningful statements and prevent studies from being biased or underpowered, it is recommended to consider an adequate number of datasets in benchmark studies^3–5^, which are as representative as possible to cover the domain of interest^6,7^. The domain of interest can be viewed as a population, of which a sample consisting of several datasets should be representative^1^. In the present study, our population corresponds to all available omics datasets of dedicated databases, from which we extract selected data characteristics, which could, in the future, be used to support the data characteristic-based interpretation of benchmark study results.

Furthermore, a better knowledge on the characteristics of experimental data of different omics disciplines helps in making simulated datasets more realistic, as shown at the example of single-cell RNA-seq data^8^. Based on these data characteristics, it could be assessed whether a simulated dataset mimics real experimental datasets realistically, which is imperative if using simulated datasets in the scope of benchmark studies^9^.

**Fig. 1:**
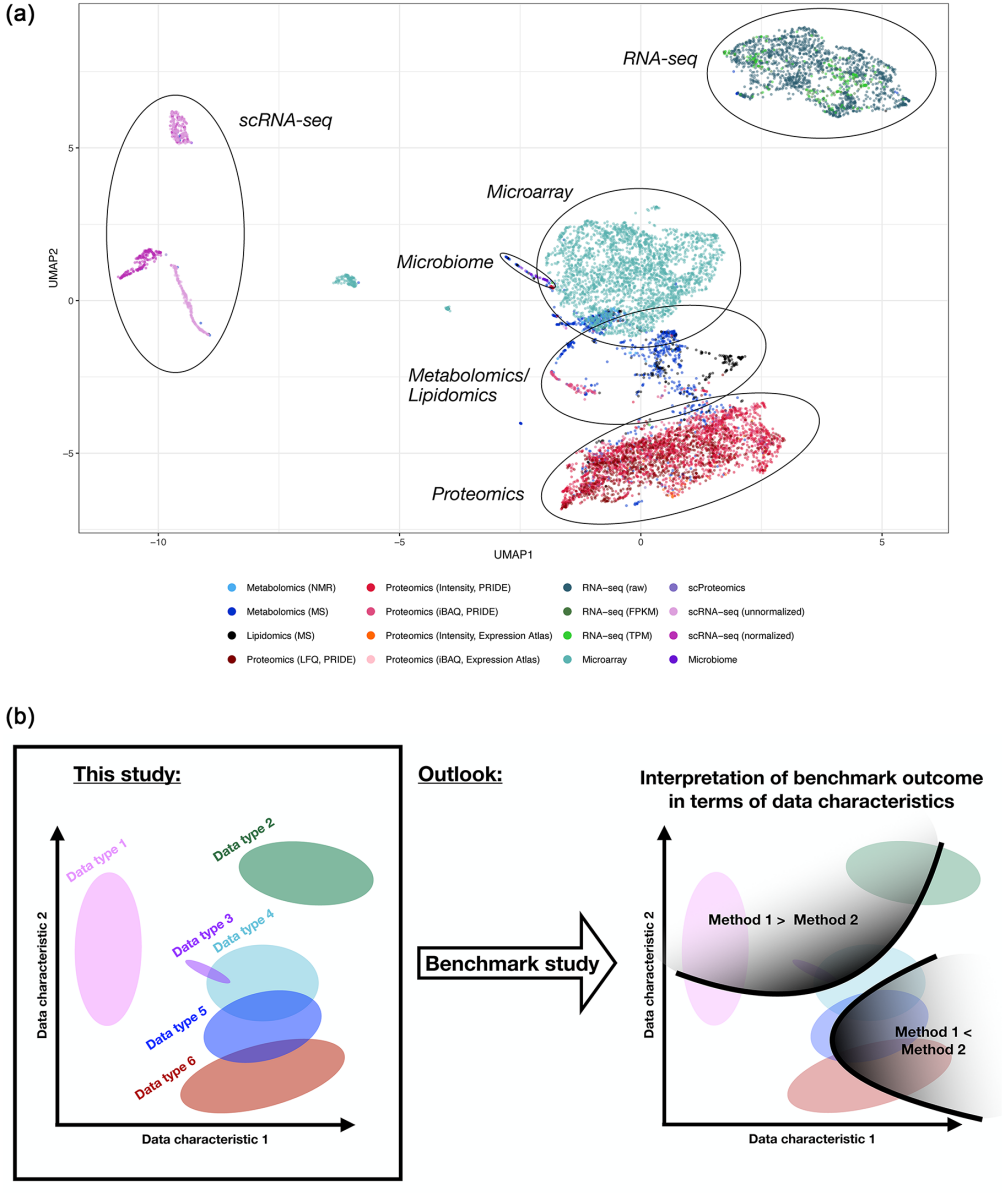
The data types cluster according to the investigated data characteristics. (**a**) Clustering results obtained using Uniform Manifold Approximation and Projection (UMAP), where each data point represents a dataset. Distinct clusters are formed for scRNA-seq, bulk RNA-seq, microarray, microbiome, metabolomics/lipidomics, and proteomics datasets. (**b**) Objective of this study and outlook.

Moreover, distinct omics communities often develop separate algorithms for similar purposes, based on presumed differences in data characteristics. Such algorithms could potentially be reused across different omics disciplines, provided they share relevant data characteristics necessary for optimal algorithm performance. A notable example is limma^10^, originally developed for differential expression analysis of microarray data, which is now also widely applied to other omics types, such as proteomics (see, e.g., van Ooijen et al. 2018^11^). Other methods such as zero-inflated negative binomial models are currently mainly used for the analysis of metagenomic omics data in the microbiome field and their applicability and performance for other omics data, such as proteomics or single-cell RNA sequencing (scRNA-seq) data, which also exhibit zero-inflation, is largely unclear.

Here, we present a descriptive study on selected characteristics of data generated by various omics disciplines. We investigate over ten thousand publicly available omics datasets, selected based on their accessibility from respective databases at the time of retrieval. To investigate typical patterns, we extracted 29 data characteristics from 10,109 datasets across 16 different omics data types - including metabolomics (mass spectrometry (MS)- and nuclear magnetic resonance (NMR)-based), lipidomics (MS-based), bulk and single-cell proteomics, transcriptomics (single-cell and bulk RNA-sequencing (RNA-seq), microarray), and microbiome data - in the form of quantitative matrices. We selected these data types (and their subtypes) as they represent typical omics data types.

## Results

### Distinct preprocessing steps are required for different data types

Prior to determining the data characteristics, all zeros were set to missing values (i.e., ‘not available’, NAs). Thus, when we refer to missing values in the following, this will also include zeros.

Subsequently, all analyte quantities were log2-transformed, and we derived 29 data characteristics (Table 1) for 16 different data types across various omics disciplines (Table 2) including metabolomics, lipidomics, proteomics, transcriptomics (RNA-seq, microarray), and microbiome. We applied the logarithm to the data characteristics ‘# Samples’, ‘# Analytes’, and ‘Variance’ to achieve more symmetrical distributions. Specifically, we used the binary logarithm (log2), as it provides more interpretable values compared to other logarithmic bases.

The data types mentioned above were further subdivided into subgroups (Supplementary Table S1) based on the criteria specified below. This subgrouping ensured that the resulting groups were sufficiently large and that the number of subgroups remained at a manageable level.

In metabolomics and lipidomics, chromatography is often conducted before MS analysis. We subdivided the Metabolomics (MS) and Lipidomics (MS) data types by chromatography method, forming subgroups for gas chromatography (GC) and liquid chromatography (LC), which were further divided based on the most commonly used MS technologies: (quadrupole) time-of-flight ((q)TOF), linear trap quadrupole (LTQ), triple quadrupole (TQ), and quadrupole (Q). Similarly, proteomics datasets derived from the PRIDE database^12^ (iBAQ, Intensity, and LFQ) were split by MS technology, creating subgroups for Orbitrap, Q Exactive, maXis, TripleTOF, and timsTOF mass spectrometers. The microarray data type was divided by manufacturer (Affymetrix and Agilent). scRNA-seq datasets were subdivided into datasets generated by droplet-based or SMART-like technologies. Finally, microbiome datasets were categorized into 16S amplicon or whole genome shotgun sequencing (WGS).

In addition, the data types Metabolomics (NMR), Proteomics (iBAQ, Expression Atlas), Proteomics (Intensity, Expression Atlas), scProteomics, RNA-seq (raw), RNA-seq (FPKM), and RNA-seq (TPM), which were not further subdivided, are included in the comparison, resulting in a total of 38 subgroups.

Each data type required a distinct preprocessing procedure to prepare it for consistent calculation of data characteristics. The data characteristics were selected based on their anticipated impact on the performance of downstream analysis methods. The resulting table with the data characteristics for each investigated dataset is provided in the supplemental material and may benefit future studies exploring the relationship between algorithm performance and data characteristics.

**Table 1:** Data characteristics of datasets investigated in this study. The data characteristics were calculated on log2-transformed data. ‘mtx’ denotes the entire quantitative matrix of the dataset, where *i* refers to analytes (rows) and and *j* to samples (columns). NIPALS = nonlinear iterative partial least squares, PCA = principal component analysis.

**Data Characteristic**	**Alias**	**Relevance**	**Pseudocode**
Log2 of number of samples	log2(# Samples)	Sample size impacts the power of statistical tests.	log2(# Samples)
Log2 of number of analytes	log2(# Analytes)	High-dimensional datasets can pose unique challenges for statistical testing, e.g., due to correlations or interactions of analytes.	log2(# Analytes)
Mean	Mean	Feature of the distribution	mean(mtx)
Median	Median	Feature of the distribution	median(mtx)
Minimum	Min	Feature of the distribution	min(mtx)
Maximum	Max	Feature of the distribution	max(mtx)
Log2 of variance	log2(Variance)	Feature of the distribution	log2(var(mtx))
Median of the variance of all analytes	median(Variance of analytes)	Feature of the distribution	median(var(mtx$$_{i.}$$))
Median of the variance of all samples	median(Variance of samples)	Feature of the distribution	median(var(mtx$$_{.j}$$))
Kurtosis	Kurtosis	Feature of the distribution	kurtosis(mtx)
Skewness	Skewness	Feature of the distribution	skewness(mtx)
Absolute skewness	|Skewness|	Feature of the distribution	abs(skewness(mtx))
Percentage of distinct values	% Distinct values	The percentage of distinct values is related to the variance of the data.	(unique(mtx)/length(mtx)) * 100
Percentage of missing values or zeros	% NA	Missing values/zeros caused by various mechanisms, are a prominent feature of many omics data types.	%NA(mtx)
Minimum of percentage of missing values across analytes	min(% NA in analytes)	Missing values/zeros caused by various mechanisms, are a prominent feature of many omics data types.	min(%NA(mtx$$_{i.}$$))
Maximum of percentage of missing values across analytes	max(% NA in analytes)	Missing values/zeros caused by various mechanisms, are a prominent feature of many omics data types.	max(%NA(mtx$$_{i.}$$))
Minimum of percentage of missing values across samples	min(% NA in samples)	Missing values/zeros caused by various mechanisms, are a prominent feature of many omics data types.	min(%NA(mtx$$_{.j}$$))
Maximum of percentage of missing values across samples	max(% NA in samples)	Missing values/zeros caused by various mechanisms, are a prominent feature of many omics data types.	max(%NA(mtx$$_{.j}$$))
Percentage of analytes with missing values	% Analytes with NAs	Missing values/zeros caused by various mechanisms, are a prominent feature of many omics data types.	%(sum(is.na(mtx$$_{i.}$$)) > 1)
Percentage of samples with missing values	% Samples with NAs	Missing values/zeros caused by various mechanisms, are a prominent feature of many omics data types.	%(sum(is.na(mtx$$_{.j}$$)) > 1)
Coefficient of the Spearman correlation between the percentage of missing values or zeros and the mean value of each analyte	Corr(Mean vs. % NA) (Analytes)	A slight negative correlation between analyte mean and the percentage of the missing values of analytes was observed for proteomics data^13^.	cor(%NA(mtx$$_{i.}$$), mean(mtx$$_{i.}$$))
Coefficient of the Spearman correlation between the percentage of missing values or zeros and the mean value of each sample	Corr(Mean vs. % NA) (Samples)	A strong correlation between sample mean and the percentage of the missing values of samples ov varying directions was observed for proteomics data^13^.	cor(%NA(mtx$$_{.j}$$), mean(mtx$$_{.j}$$))
Standard deviation of the intensity where a sample’s probability of having of a missing value is 50 %	sd(Intensity w/ prob(NA) = 50% for sample)	A higher value hints at the presence of sample-dependent detection thresholds.	sd(probNA50%(mtx$$_{.j}$$))
Standard deviation of the intensity where a sample’s probability of having of a missing value is 90 %	sd(Intensity w/ prob(NA) = 90% for sample)	A higher value hints at the presence of sample-dependent detection thresholds.	sd(probNA90%(mtx$$_{.j}$$))
Percentage of variance explained by first principal component (PC1) of NIPALS implementation of PCA	% Var. explained by PC1	PCA is often used for the exploratory data analysis of high-dimensional data^14^.	%var(PCA(t(mtx))$PC1)
Percentage of variance explained by second principal component (PC2) of NIPALS implementation of PCA	% Var. explained by PC2	PCA is often used for the exploratory data analysis of high-dimensional data^14^.	%var(PCA(t(mtx))$PC2)
Agglomerative coefficient of hierarchical clustering of analytes	Agglom. coef. hierarch. analyte clustering	Measure of the magnitude of clustering	coef.hclust(hcluster(mtx$$_{i.}$$))
Linear coefficient of second order polynomial fit with analyte means against analyte variances	Lin. coef. of Poly2(Means vs. Vars) (Analytes)	Quantitative measure for the kind of overdispersion	Lin.coef(var(mtx$$_{i.}$$) $$\sim$$ mean(mtx$$_{i.}$$) + I(mean(mtx$$_{i.}$$)$$^2$$))
Quadratic coefficient of second order polynomial fit with analyte means against analyte variances	Quadr. coef. of Poly2(Means vs. Vars) (Analytes))	Quantitative measure for the kind of overdispersion	Quadr.coef(var(mtx$$_{i.}$$) $$\sim$$ mean(mtx$$_{i.}$$) + I(mean(mtx$$_{i.}$$)$$^2$$))

**Table 2:** Data types investigated in this study (with more than 5 datasets).

**Data type**	**Alias**	**Number Of Datasets**	**Database Name**	**File Name Pattern**
Metabolomics data generated by nuclear magnetic resonance (NMR)	Metabolomics (NMR)	102	MetaboLights$$^\textrm{c}$$^15^	^m_.*$$\backslash \backslash$$ .tsv$ (if file name contains ‘NMR’)$$^\textrm{b}$$
Metabolomics data generated by mass spectrometry (MS)	Metabolomics (MS)	887	MetaboLights$$^\textrm{c}$$^15^	^m_.*$$\backslash \backslash$$ .tsv$ (if file name does not contain ‘NMR’)
Lipidomics data generated by MS	Lipidomics (MS)	324	MetaboLights$$^\textrm{c}$$^15^	^m_.*$$\backslash \backslash$$ .tsv$ (if file name does not contain ‘NMR’)
Label-free protein-level proteomics data generated in data-dependent acquisition (DDA) mode and processed by MaxQuant$$^\textrm{a}$$^16^	Proteomics ([iBAQ, Intensity, LFQ], PRIDE)	iBAQ: 569, Intensity: 1492, LFQ: 1076	PRIDE$$^\textrm{d}$$^12^	proteinGroups$$^\textrm{b}$$
Label-free protein-level proteomics data generated in DDA mode and processed by MaxQuant$$^\textrm{a}$$^16^	Proteomics ([iBAQ, Intensity], Expression Atlas)	iBAQ: 43, Intensity: 43	Expression Atlas$$^\textrm{e}$$^17^	-proteinGroups.txt$$$^\textrm{b}$$
MS-based single-cell proteomics (SCP) data	scProteomics	15	scpdata R package^18,19^ (Snapshot date: 31/10/2022)	
Normalized microarray data (as is provided on Expression Atlas website https://www.ebi.ac.uk/gxa/home)	Microarray	2948	Expression Atlas$$^\textrm{e}$$^17^	-normalized-expressions.tsv$$$^\textrm{b}$$
Raw RNA-seq data and undecorated raw RNA-seq data (comes with FPKM and TPM datasets, undecorated meaning that Gene names are not added)	RNA-seq (raw)	1571	Expression Atlas$$^\textrm{e}$$^17^	-raw-counts.tsv$ and -raw-counts.tsv.undecorated
Median of RNA-seq data after Fragments Per Kilobase Million (FPKM) normalization	RNA-seq (FPKM)	153	Expression Atlas$$^\textrm{e}$$^17^	-fpkms.tsv
Median of RNA-seq data after Transcripts Per Kilobase Million (TPM) normalization	RNA-seq (TPM)	156	Expression Atlas$$^\textrm{e}$$^17^	-tpms.tsv
Unnormalized single-cell RNA sequencing (scRNA-seq) data	scRNA-seq (unnormalized)	349	Single Cell Expression Atlas$$^\textrm{f}$$^17,20^	.aggregated$$\_$$filtered$$\_$$counts.mtx
ScRNA-seq data normalised to counts per million	scRNA-seq (normalized)	349	Single Cell Expression Atlas$$^{f}$$^17,20^	.aggregated$$\_$$filtered$$\_$$normalised$$\_$$counts.mtx
Microbiome data containing taxon abundances from 16S amplicon or whole genome shotgun sequencing (WGS) studies	Microbiome	31	MicrobiomeDB$$^{g}$$^21^	taxon$$\_$$abundance.tsv

$^a$MaxQuant^16^ proteomics data are analysed separately for ‘iBAQ’, ‘Intensity’, and ‘LFQ’ columns.

$$^b$$ Case ignored.

$^c$ ftp://ftp.ebi.ac.uk/pub/databases/metabolights/studies/public/

$^d$ ftp://ftp.pride.ebi.ac.uk/pride/data/archive/

$^e$ ftp://ftp.ebi.ac.uk/pub/databases/microarray/data/atlas/experiments/

$^f$ ftp://ftp.ebi.ac.uk/pub/databases/microarray/data/atlas/sc_experiments/

$^g$ https://microbiomedb.org/mbio/app/search/dataset/Studies/result

**Fig. 2:**
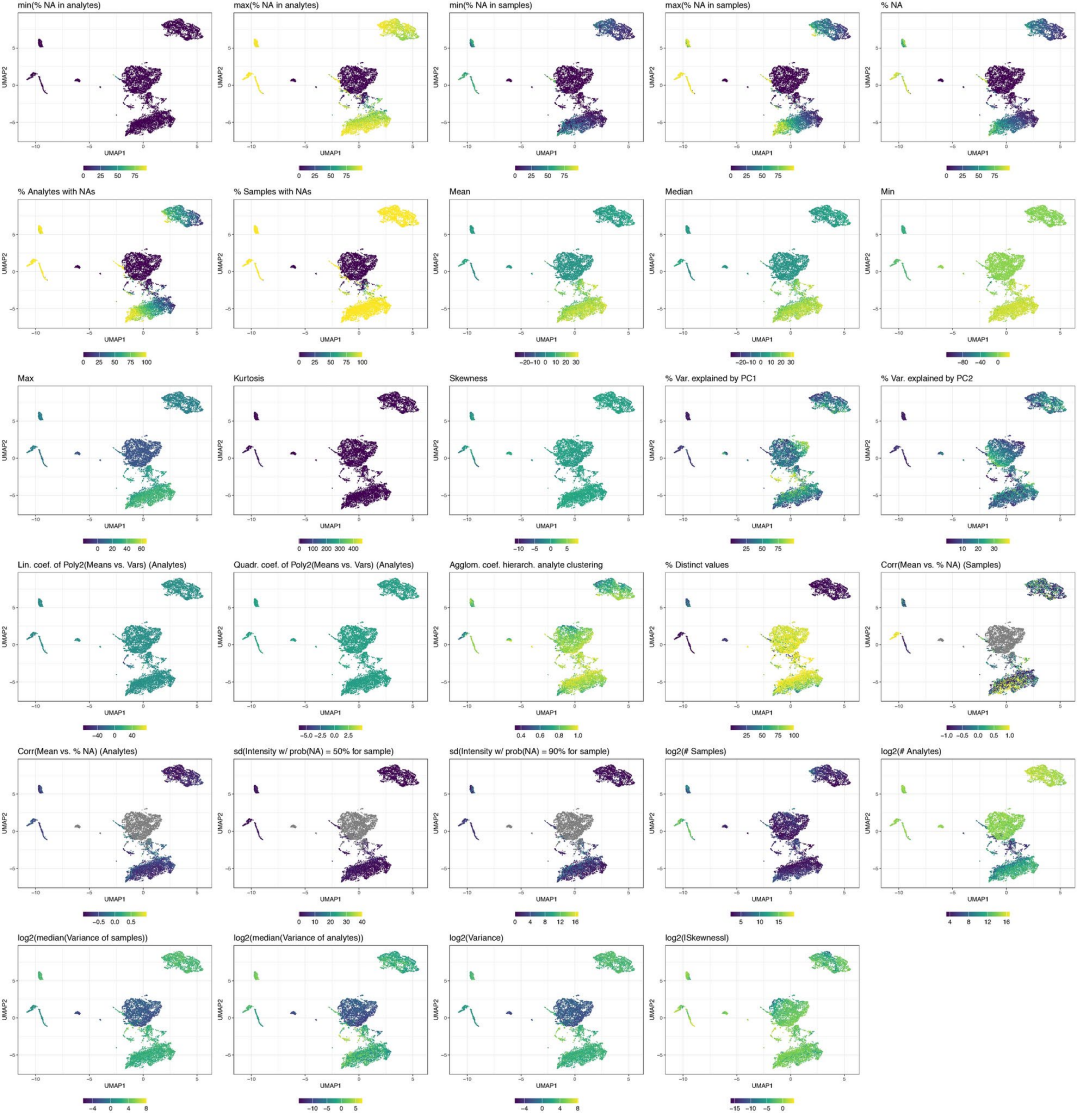
Visualization of how data characteristics contribute to the clustering of different data types in a Uniform Manifold Approximation and Projection (UMAP). The subplots are color-coded according to the values of the investigated data characteristics. Grey points represent datasets for which no information on the respective data characteristic is available.

### Low-dimensional representation reveals clustering of different data types

Based on the 29 data characteristics, distinct clusters of certain data types are visible using Uniform Manifold Approximation and Projection (UMAP) (Figure 1a, Supplementary Figure S1) and nonlinear iterative partial least squares (NIPALS)-based principal component analysis (PCA) (Supplementary Figure S2). NIPALS was chosen as it accommodates missing values, allowing the inclusion of data characteristics containing missing data.

The clustering indicates that most data types investigated have combinations of data characteristics specific to them. This knowledge of the data characteristics of omics datasets can, in the future, inform the data-driven selection of optimal downstream methods (such as normalization) and aid in interpreting benchmark results (Figure 1b).

In the PCA results, the microarray datasets form the most distinct and prominent cluster, while the metabolomics and lipidomics datasets are the most dispersed, overlapping with bulk RNA-seq and proteomics data types (Supplementary Figure S2).

Since UMAP cannot be applied to data with missing values, the following data characteristics were excluded due to having too many missing values: ‘Corr(Mean vs. % NA) (Samples)’, ‘Corr(Mean vs. % NA) (Analytes)’, ‘sd(Intensity w/ prob(NA) = 50% for sample)’, and ‘sd(Intensity w/ prob(NA) = 90% for sample)’. Additionally, two proteomics datasets were excluded for having missing values in the remaining data characteristics.

The contribution of individual data characteristics to the UMAP clustering is shown in Figure 2. Some example data characteristics contributing to cluster separation include ‘% Analytes with NAs’ and ‘% Samples with NAs’, both of which are small for the microarray datasets cluster, as these datasets contain no missing values. The number of measured analytes (‘log2(# Analytes)’) is lowest for metabolomics and lipidomics datasets, while the number of samples (‘log2(# Samples)’) is highest for scRNA-seq datasets. The single-cell and bulk RNA-seq clusters exhibit a low percentage of distinct values (‘% Distinct values’). Additionally, some proteomics datasets and the cluster of normalized scRNA-seq datasets show a high correlation between the mean and the missing value proportion (‘Corr(Mean vs. % NA) (Samples)’), an informative metric for characterizing detection limits, as discussed below.

**Fig. 3:**
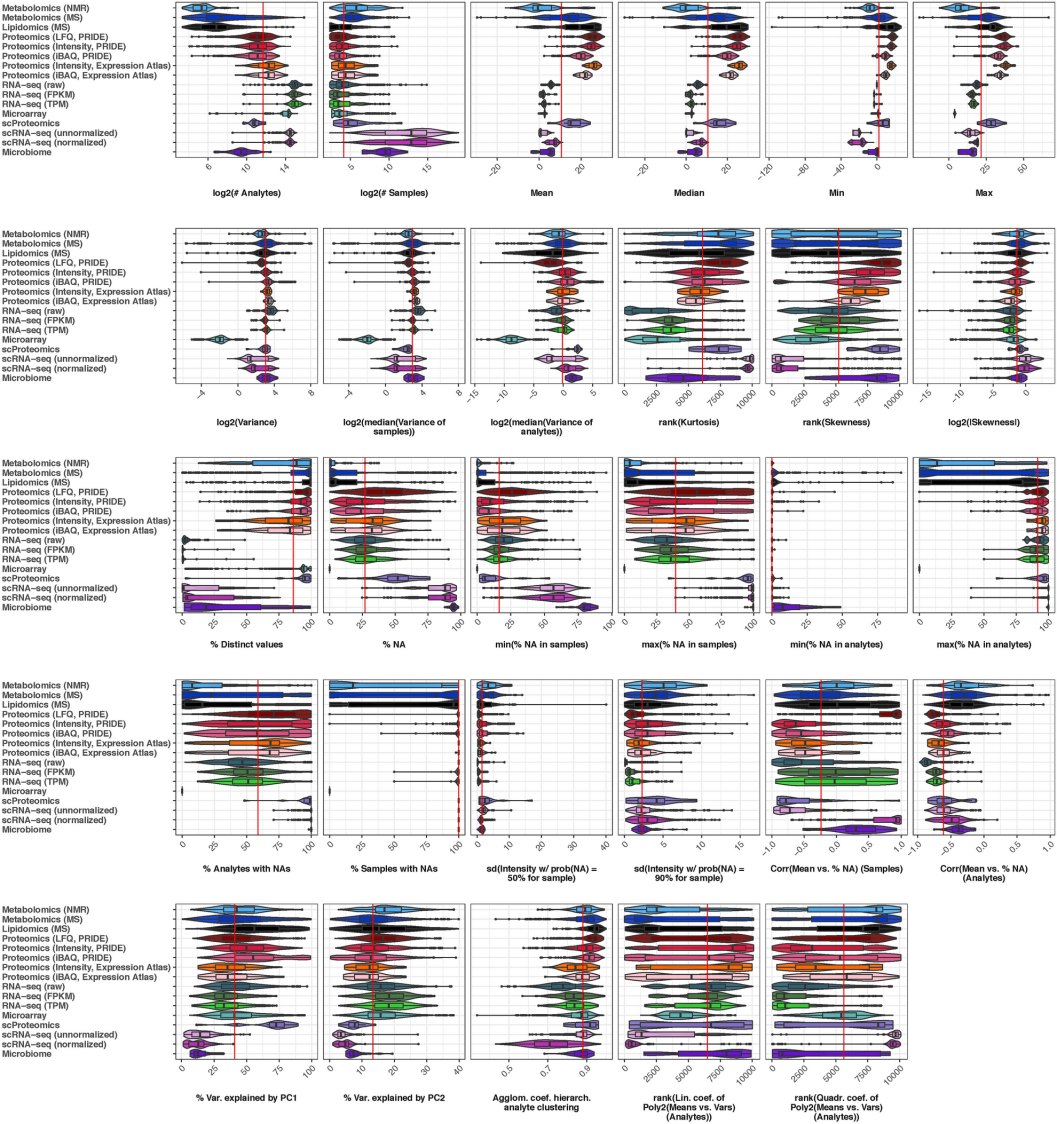
For most investigated data characteristics, substantial differences exist between the data types. The vertical red lines represent the median of the medians for each data type. For better comparison, the rank is displayed for ‘Kurtosis’, ‘Skewness’, ‘Lin. coef. of Poly2(Means vs. Vars)(Analytes)’, and ‘Quadr. coef. of Poly2(Means vs. Vars)(Analytes)’.

### Distribution of individual data characteristics varies across data types

Following the global view of data characteristic-based clustering, we now examine how individual data characteristics are distributed across the investigated data types (Figure 3) and their subgroups (Supplementary Figure S3). For better comparison, the ranks of ‘Kurtosis’, ‘Skewness’, ‘Lin. coef. of Poly2(Means vs. Vars)(Analytes)’, and ‘Quadr. coef. of Poly2(Means vs. Vars)(Analytes)’ are displayed instead of the absolute values. Distributions of the absolute values and ranks for all data characteristics are shown in Supplementary Figures S4 and S5 and Supplementary Figures S6 and S7, respectively.

Supplementary Table S2 summarizes the most notable trends, highlighting which data types stand out for specific data characteristics, some of which are discussed below.

The generally large variation in certain data characteristics observed for metabolomics and lipidomics datasets may be due to less standardized data processing compared to other omics data types^22^.

Since microarray datasets contain no missing values or zeros, the missing value-related characteristics ‘% NA’, ‘min(% NA in samples)’, ‘max(% NA in samples)’, ‘max(% NA in analytes)’, ‘% Analytes with NAs’, and ‘% Samples with NAs’ are lowest for microarray datasets. For the same reason, no values are displayed for microarray datasets for ‘Corr(Mean vs. % NA) (Samples)’, ‘Corr(Mean vs. % NA) (Analytes)’, ‘sd(Intensity w/ prob(NA) = 50% for sample)’, and ‘sd(Intensity w/ prob(NA) = 90% for sample)’.

Most data characteristics differ strongly between droplet-based and SMART-like scRNA-seq data (Supplementary Figure S3), resulting in two distinct groups in the violin plots when these subgroups are combined (Figure 3). Similarly, 16S and WGS microbiome datasets differ in many characteristics, as 16S datasets represent count data, while WGS datasets are compositional, with taxa percentages summing to 100%.

Metabolomics and lipidomics datasets contain the fewest analytes, while transcriptomics data (microarray as well as bulk and single-cell RNA-seq) contain the most. However, scRNA-seq and microbiome datasets, known for their sparsity, have the highest number of samples and the largest percentage of missing values (Figure 3). These data types also exhibit the smallest contributions of the first two principal components in a PCA, indicating that much of the variation in microbiome and scRNA-seq datasets is not explained by these components.

The variance-related values ‘log2(Variance)’, ‘log2(median(Variance of samples))’, and ‘log2(median(Variance of analytes))’ are smaller for droplet-based scRNA-seq data than for SMART-like scRNA-seq data. The same is true when comparing 16S microbiome data to WGS microbiome data. Moreover, ‘% Distinct values’ is lowest for bulk and single-cell RNA-seq data, Agilent microarray data, and 16S microbiome data.

The data-dependent acquisition (DDA) proteomics datasets analyzed in this study were processed using MaxQuant^16^, which reports protein quantities as Intensity, intensity-based absolute quantification (iBAQ)^23^, and label-free quantification (LFQ)^24^values. Across all measures – Intensity, iBAQ, and LFQ – the overall median is higher compared to other data types, regardless of whether the datasets were derived from the PRIDE or Expression Atlas^17,20^ databases. However, LFQ stands out, along with normalized droplet-based scRNA-seq datasets, for having the highest ‘Corr(Mean vs. % NA) (Samples)’ value, indicating a strong positive correlation between missing values and the mean across samples. This positive correlation may lead to biased normalization results. Notably, when considering all data types, ‘Corr(Mean vs. % NA) (Samples)’ values range from negative to positive, while ‘Corr(Mean vs. % NA) (Analytes)’ predominantly shows negative correlations.

### Correlation between data characteristics varies across data types

When examining the Spearman correlations between selected data characteristics across the different data types (Supplementary Figures S8 to S23) and their subgroups (Supplementary Figures S24 to S54), it is important to consider the differences in the number of datasets per data type. The number of datasets included affects the reliability of the detected correlations, i.e., data types with fewer datasets may have less power to detect correlations compared to those with more datasets. Thus, we merely include the corresponding correlation plots for the sake of completeness.

In the correlation plots, each data point represents a single dataset. Some correlations are common across data types, such as the positive correlation between ‘log2(# Samples)’ and ‘max% NA in analytes)’, between ‘log2(|Skewness|)’ and ‘Kurtosis’, and between ‘sd(Intensity w/ prob(NA) = 50% for sample)’ and ‘sd(Intensity w/ prob(NA) = 90% for sample)’. There is also a negative correlation between ‘Lin. coef. of Poly2(Means vs. Vars) (Analytes)’ and ‘Quadr. coef. of Poly2(Means vs. Vars) (Analytes)’.

However, differences in correlations can also be observed, such as those between microarray datasets generated by different manufacturers. While Affymetrix microarray datasets show a negative correlation between ‘Mean’ and ‘log2(Variance)’, this is not the case for Agilent datasets. Agilent datasets, on the other hand, show a negative correlation between ‘log2(Variance)’ and both ‘Skewness’ and ‘Kurtosis’. Additionally, while Affymetrix datasets display both positive and negative skewness, most Agilent datasets exhibit positive skewness (Supplementary Figure S5).

### ‘Corr(Mean vs. % NA) (Samples)’ and normalization: An example of a data characteristic impacting downstream analysis

**Fig. 4:**
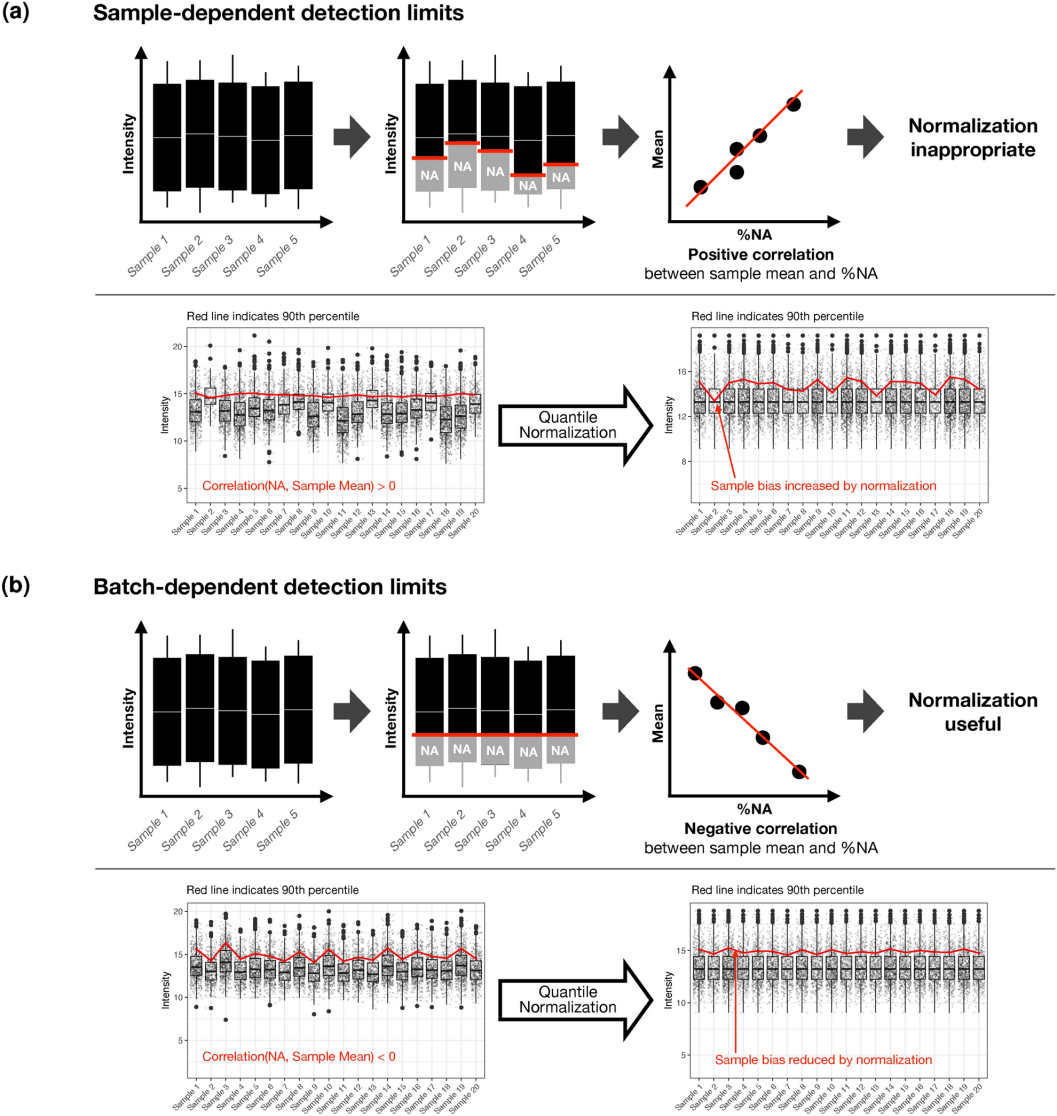
Relationship between sample- and batch-dependent detection limits, sample mean-missing value (NA) correlations, and the appropriateness of a normalization step. (**a**) A dataset with sample-dependent detection limits shows a positive correlation. This positive correlation indicates that normalization is inappropriate – demonstrated by quantile normalization applied to a simulated dataset with sample-dependent detection limits – as it increases sample bias. The red line, representing the 90th percentile, is used as a reference, as it should remain relatively unaffected by bias caused by small intensity values becoming missing values. (**b**) A dataset with batch-dependent detection limits shows a negative correlation. This negative correlation indicates that normalization is beneficial – demonstrated by quantile normalization applied to a simulated dataset with batch-dependent detection limits – as it decreases sample bias.

As previously suggested, if a data characteristic is assumed to influence the performance of certain algorithms, its causal influence can be confirmed through simulation experiments in which the respective data characteristic is systematically altered^25^. In line with this notion – and using the ‘Corr(Mean vs. % NA) (Samples)’ data characteristic as an example of how data characteristics can influence downstream analyses – we illustrate a mechanism that explains the positive and negative correlation between missing values and sample means (Figure 4). Specifically, sample-dependent detection limits, which vary between samples, cause a positive correlation when they result in missing values (Figure 4a). In contrast, batch-dependent detection limits, which are consistent across all measured samples, and detection limits that result in zeros cause a negative correlation (Figure 4b).

Using simulations, we show that in the case of a positive correlation originating from sample-dependent detection limits, conducting quantile normalization increases sample bias and should therefore be avoided (Figure 4a). In contrast, for a negative correlation originating from batch-dependent detection limits, normalization reduces sample bias and therefore is beneficial (Figure 4b). This rule also applies to datasets that contain zeros instead of NAs. For such datasets, the correlation between missing quantities – in this case quantified with zeros as measured values – and sample means is negative for both sample- and batch-dependent detection limits and, thus, normalization is beneficial (though different normalization methods may be more suitable compared to those used for datasets with NAs). The negative correlation arises because zeros are included in the calculation of the sample mean, unlike NAs, which are excluded.

This illustrates that understanding data characteristics, such as the correlation between missing values and sample means, can guide decisions on whether to apply normalization. While this provides an example of how a data characteristic can influence downstream analyses, a comprehensive evaluation of the relationships between data characteristics and the performance of analysis methods is beyond the scope of this publication and will be addressed in future research.

### Provided tools

To aid further exploration, we provide a simulation tool, accessible at https://missingvaluesimulation.imbi.uni-freiburg.de (Supplementary Figure S55, code available at https://github.com/kreutz-lab/dataCharacteristics/tree/main/shinyApps). This tool allows users to explore the effects of sample- or batch-dependent detection limits on the correlation between sample means and missing quantities, compared to random missing quantities, with the option to represent missing quantities as either NAs or zeros.

Additionally, we provide a tool accessible at https://omicscharacterization.imbi.uni-freiburg.de/, which allows researchers to upload their datasets (with samples in columns and analytes in rows) and, using boxplots, the NIPALS implementation of PCA, and UMAP, assess how well their data fits within the set of datasets analyzed in this study for a specific omics discipline (Supplementary Figure S56). For files larger than 100 MB, the tool can be run using the code available at https://github.com/kreutz-lab/dataCharacteristics/tree/main/shinyApps by increasing the ’shiny.maxRequestSize’ parameter. This tool allows researchers to detect outliers, assess data quality, and evaluate how representative an omics benchmark dataset is for a given omics discipline.

## Discussion

As there is no general strategy for selecting data characteristics, we chose characteristics that represent common summary statistics, along with additional characteristics that may be relevant in specific contexts. Specifically, we included data characteristics related to missing values (e.g., the percentage of missing values within the data matrix) and data distribution (e.g., skewness and kurtosis), as well as more general characteristics (e.g., the number of samples, the number of analytes, and the mean of the data matrix) and higher-level characteristics (e.g., the percentage of variation explained by the first two principal components of a PCA performed on the data matrix). Data characteristics reliant on biological meta-information, such as sample groupings that may provide information about the biological relationship of the samples, were not included. In general, we cannot account for the biological meta-information of samples, such as correlations arising from shared origin, treatment, condition, time point, etc., because this information is too heterogeneous to be appropriately accounted for, and not available in a standardized format in the considered databases. Furthermore, the separation in the low-dimensional representations depends on the selected data characteristics and may differ if other characteristics were used to characterize the data and generate the low-dimensional representations. Different data properties may prove informative depending on the analysis in question. Identifying key data characteristics that significantly impact algorithm performance will be essential in the future for developing more robust analysis methods and selecting the most suitable approach for a specific dataset.

To ensure that differences between different omics types were not simply due to some omics types containing zeros while others contain NAs, we converted all zeros to NAs to make the datasets more comparable. Additionally, setting all NAs to zero would have introduced more bias in the calculation of data characteristics than the reverse. Since our manuscript considers and averages over a huge number of biological conditions and, thus, focuses on differences between measurement techniques, we do not and cannot distinguish between structural and real zeros and NAs, i.e., we are unable to distinguish between different origins (e.g., insufficient sequencing depth vs. analyte concentrations below technical detection limits). For simplicity, we also treat individual cells in scRNA-seq datasets as separate samples, even though multiple cells may originate from the same individual.

## Conclusion

Data characteristics play a crucial role in determining algorithm performance. This comprehensive descriptive study of data characteristics across various omics data types provides a valuable foundation for future research investigating how these characteristics influence algorithm effectiveness. The observed differences in data characteristics may serve as a guide for method selection in omics analyses.

Our work complements existing frameworks that explore these associations within benchmark studies, such as the model-based recursive partitioning approach introduced by Eugster et al.^25^, and ‘Omnibenchmark’^26^, a community-driven platform that systematically organizes benchmarks while continuously updating the set of available algorithms and datasets.

An important insight from this study is the potential for transferring algorithms between omics types by identifying the key data characteristics that impact performance. For example, we observed a positive correlation between sample means and the percentage of missing values in DDA proteomics data processed by the MaxLFQ algorithm^24^in MaxQuant^16^, a pattern also seen in a recent benchmarking study^13^ for DIA proteomics datasets processed by certain DIA software packages. This positive correlation can be caused by sample-dependent detection limits and suggests that applying normalization may introduce bias. In contrast, negative correlations – arising from batch-dependent detection limits – indicate that normalization is necessary. Interestingly, DDA proteomics datasets not processed by MaxLFQ did not show this positive correlation, underscoring that data characteristics can be entirely altered by a single processing step. This finding highlights the importance of understanding how preprocessing influences data characteristics and downstream analysis decisions.

Looking ahead, we aim to uncover additional connections between specific data characteristics and the performance of downstream methods. Furthermore, we plan to expand our analysis to include biological background information, offering a more holistic view of how both technical and biological factors influence data characteristics.

We encourage researchers to leverage the extensive knowledge of omics data characteristics compiled in this study, not only for evaluating the similarity of omics datasets but also for optimizing downstream analysis strategies based on these characteristics.

## Methods

10,110 omics datasets were downloaded on January 6, 2023. For microbiome data, datasets were retrieved from the MicrobiomeDB^21^ website (https://microbiomedb.org/mbio/app/search/dataset/Studies/result, Release 31). For all other data types, project folders within relevant databases were searched for files matching a defined file name pattern (Table 2). The URLs of these files were used to download them via the command-line tool ‘wget’.

For the analysis of proteomics data processed with MaxQuant^16^, we analyzed the ‘iBAQ’, ‘Intensity’, and ‘LFQ’ columns separately when available. Fragments Per Kilobase Million (FPKM) and Transcripts Per Kilobase Million (TPM) files were processed using the median value, corresponding to the third of the five comma-separated numbers in each cell, for further analysis.

Datasets from the MetaboLights^15 ^database were manually screened to identify those containing primarily, with only few exceptions, quantitative information on fatty acids or lipids. These datasets were classified as the ‘Lipidomics’ data type, while all other MetaboLights datasets were classified as ‘Metabolomics’. Since only one nuclear magnetic resonance (NMR)-based lipidomics dataset was among the downloaded datasets, we chose not to create a separate group for NMR-based lipidomics. Consequently, only the remaining 10,109 datasets were included in this descriptive study. Single-cell RNA-seq datasets were further subdivided into those generated by droplet-based or SMART-like technologies^20^, based on bimodal patterns observed in data characteristics when both technologies were analyzed together. This classification was determined by the presence of the term ‘Alevin’ in the ‘[Project ID].software.tsv’ file in the project-specific FTP folder, as Expression Atlas used the Alevin pipeline^27^ for droplet-based scRNA-seq processing.

Information regarding specific metabolomics technologies used to generate mass spectrometry (MS) datasets was obtained from https://www.ebi.ac.uk/metabolights/ws/studies/technology. For PRIDE proteomics datasets, MS instrument information was extracted using the ‘PXDataset()’ function from the ‘rpx’ R package (version 2.6.3)^28^. Microarray manufacturers were identified from the json file in the project-specific folder at http://ftp.ebi.ac.uk/biostudies/nfs/.

Zeros and infinite values (‘Inf’, as in RNA-seq transcript data) were set to ‘not available’ (‘NA’). Duplicate datasets – e.g., re-uploaded datasets under different file names or project IDs – were removed. Data characteristics were calculated for datasets containing at least 5 experimental samples and 10 analytes. Further analysis was limited to datasets with total variance, median sample variance, and median analyte variance greater than zero, and to data types and subgroups with more than 5 datasets.

Given the peculiarities of each data type, we conducted data type-specific cleaning before analyzing the data characteristics. For instance, the specific naming conventions of relevant quantitative dataset columns were considered, and datasets with negative values (potentially due to prior log transformation) or with a median variance of zero across analytes were excluded. However, due to the automated nature of the analysis workflow, some aberrant datasets may still have been included. Furthermore, we applied a logarithmic transformation (log2) to the data values for each data type.

For computationally demanding calculations of the data characteristics ‘sd(Intensity w/ prob(NA) = 50% for sample)’, ‘sd(Intensity w/ prob(NA) = 90% for sample)’, and ‘Agglom. coef. hierarch. analyte clustering’, subsets of rows and/or columns were used. Specifically, for datasets with more than 200 samples, a random subset of 200 samples was drawn for calculating ‘sd(Intensity w/ prob(NA) = 50% for sample)’ and ‘sd(Intensity w/ prob(NA) = 90% for sample)’. For the calculation of ‘Agglom. coef. hierarch. analyte clustering’, only the 500 analytes with the fewest missing values were included if a dataset contained more than 500 analytes, and a random subset of 500 samples was included if a dataset had more than 500 samples.

## Supplementary Information

Supplementary Information.

## Data Availability

The data generated in the scope of this study, including the calculated data characteristics for each of the investigated omics datasets, are provided as supplemental material and at https://github.com/kreutz-lab/dataCharacteristics/Results_csv. The code used for acquiring and processing these datasets is provided at https://github.com/kreutz-lab/dataCharacteristics.
