# Supplementary material for: Characterizing the omics landscape based on 10,000+ datasets
Source: Sci Rep. 2025 Jan 25;15:3189. doi: 10.1038/s41598-025-87256-5 (PMC11762699; doi:10.1038/s41598-025-87256-5)
Supplement: Supplementary file 1 — Supplementary Information. [file 41598_2025_87256_MOESM1_ESM.pdf]

Supplementary Material

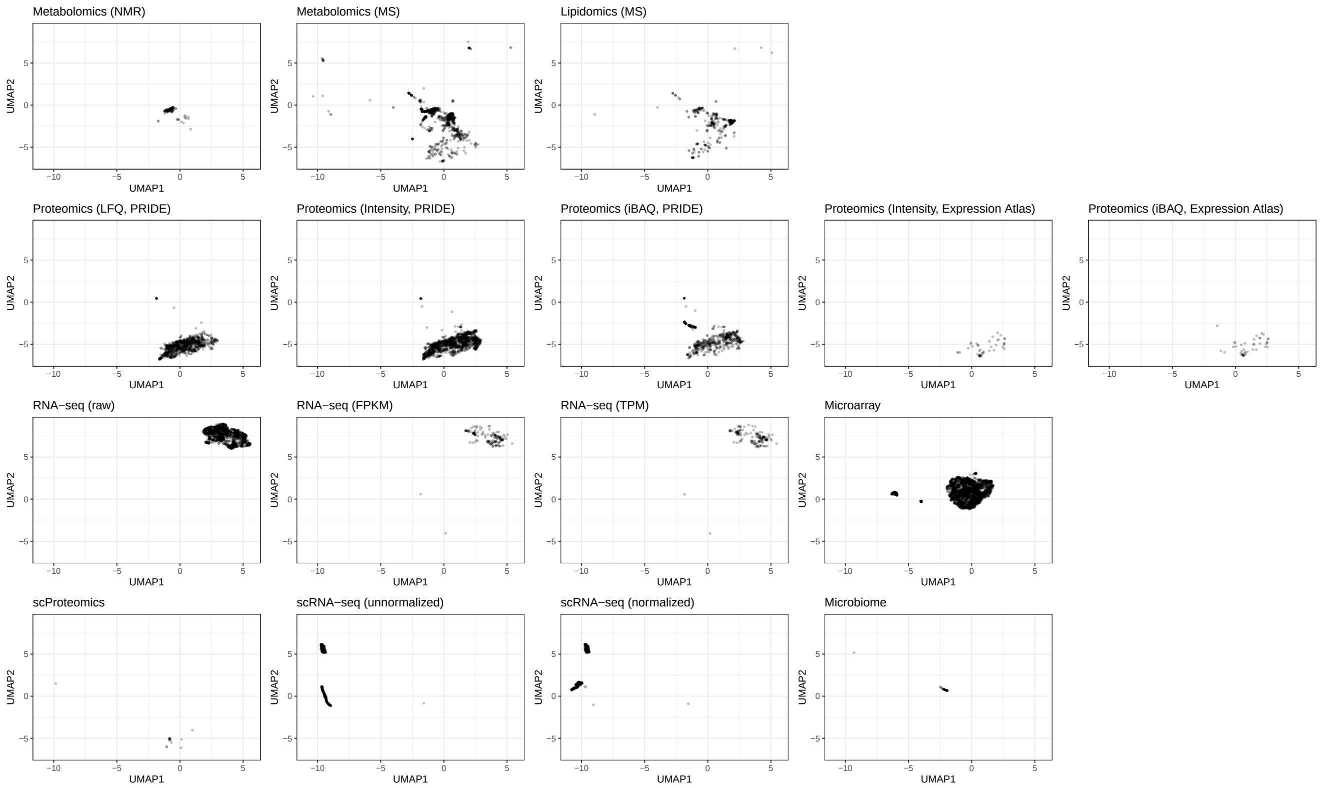

Figure S1. Different data types form distinct clusters at various positions in a Uniform Manifold Approximation and Projection (UMAP) plot, generated based on their data characteristics. In this figure, the data types shown together in the UMAP of Figure 1a are presented as separate subplots.

Table S1. Data type subgroups investigated in this study (with more than 5 datasets). For additional details on the higher-level data types, refer to Table 2.

Data type subgroup	Alias	Higher-level data type	Number Of Datasets	Database Name
Metabolomics data generated by gas chromatography (GC)-time-of-flight (TOF) or GC-quadrupole time-of-flight(qTOF) mass spectrometry (MS)	Metabolomics (GC-MS, TOF)	Metabolomics (MS)	54	MetaboLights[15]
Metabolomics data generated by GC-triple quadrupole (TQ) MS	Metabolomics (GC-MS, TQ)	Metabolomics (MS)	14	MetaboLights[15]
Metabolomics data generated by GC-quadrupole (Q) MS	Metabolomics (GC-MS, Q)	Metabolomics (MS)	47	MetaboLights[15]
Metabolomics data generated by liquid chromatography (LC)-TOF or LC-qTOF MS	Metabolomics (LC-MS, TOF)	Metabolomics (MS)	232	MetaboLights[15]
Metabolomics data generated by LC-linear trap quadrupole (LTQ) MS	Metabolomics (LC-MS, LTQ)	Metabolomics (MS)	258	MetaboLights[15]
Metabolomics data generated by LC-triple quadrupole (TQ) MS	Metabolomics (LC-MS, TQ)	Metabolomics (MS)	42	MetaboLights[15]
Lipidomics data generated by LC-TOF or LC-qTOF MS	Lipidomics (LC-MS, TOF)	Lipidomics (MS)	62	MetaboLights[15]
Lipidomics data generated by LC-LTQ MS	Lipidomics (LC-MS, LTQ)	Lipidomics (MS)	60	MetaboLights[15]
Lipidomics data generated by LC-TQ MS	Lipidomics (LC-MS, TQ)	Lipidomics (MS)	10	MetaboLights[15]
Label-free protein-level proteomics data generated in data-dependent acquisition (DDA) mode on an Orbitrap mass spectrometer and processed by MaxQuant[16]	Proteomics ([iBAQ, Intensity, LFQ], PRIDE, Orbitrap)	Proteomics ([iBAQ, Intensity, LFQ], PRIDE)	iBAQ: 186, Intensity: 489, LFQ: 337	PRIDE[12]
Label-free protein-level proteomics data generated in DDA mode on a Q Exactive mass spectrometer and processed by MaxQuant[16]	Proteomics ([iBAQ, Intensity, LFQ], PRIDE, Q Exactive)	Proteomics ([iBAQ, Intensity, LFQ], PRIDE)	iBAQ: 300, Intensity: 793, LFQ: 612	PRIDE[12]
Label-free protein-level proteomics data generated in DDA mode on a maXis mass spectrometer and processed by MaxQuant[16]	Proteomics ([Intensity, LFQ], PRIDE, maXis)	Proteomics ([Intensity, LFQ], PRIDE)	Intensity: 9, LFQ: 8	PRIDE[12]
Label-free protein-level proteomics data generated in DDA mode on a TripleTOF mass spectrometer and processed by MaxQuant[16]	Proteomics ([iBAQ, Intensity, LFQ], PRIDE, TripleTOF)	Proteomics ([iBAQ, Intensity, LFQ], PRIDE)	iBAQ: 12, Intensity: 17, LFQ: 16	PRIDE[12]
Label-free protein-level proteomics data generated in DDA mode on a timsTOF mass spectrometer and processed by MaxQuant[16]	Proteomics ([iBAQ, Intensity, LFQ], PRIDE, timsTOF)	Proteomics ([iBAQ, Intensity, LFQ], PRIDE)	iBAQ: 7, Intensity: 8, LFQ: 7	PRIDE[12]
Normalized microarray data (as is provided on Expression Atlas website https://www.ebi.ac.uk/gxa/home) generated using a Affymetrix microarray	Microarray (Affymetrix)	Microarray	2816	Expression Atlas[17]
Normalized microarray data (normalized as is provided on Expression Atlas website https://www.ebi.ac.uk/gxa/home) generated using a Agilent microarray	Microarray (Agilent)	Microarray	127	Expression Atlas[17]
Unnormalized single-cell RNA sequencing (scRNA-seq) data generated by SMART-like technologies	scRNA-seq (SMART-like, unnormalized)	scRNA-seq (unnormalized)	135	Single Cell Expression Atlas[17,20]
Unnormalized scRNA-seq data generated by droplet-based technologies	scRNA-seq (Droplet-based, unnormalized)	scRNA-seq (unnormalized)	214	Single Cell Expression Atlas[17,20]
ScRNA-seq data generated by SMART-like technologies and normalised to counts per million	scRNA-seq (SMART-like, normalized)	scRNA-seq (normalized)	135	Single Cell Expression Atlas[17,20]
ScRNA-seq data generated by droplet-based technologies and normalised to counts per million	scRNA-seq (Droplet-based, normalized)	scRNA-seq (normalized)	214	Single Cell Expression Atlas[17,20]
Microbiome data containing taxon abundances from 16S amplicon studies	Microbiome (16S)	Microbiome	22	MicrobiomeDB[21]
Microbiome data containing compositional taxon abundances from whole genome shotgun sequencing (WGS) studies	Microbiome (WGS)	Microbiome	9	MicrobiomeDB[21]

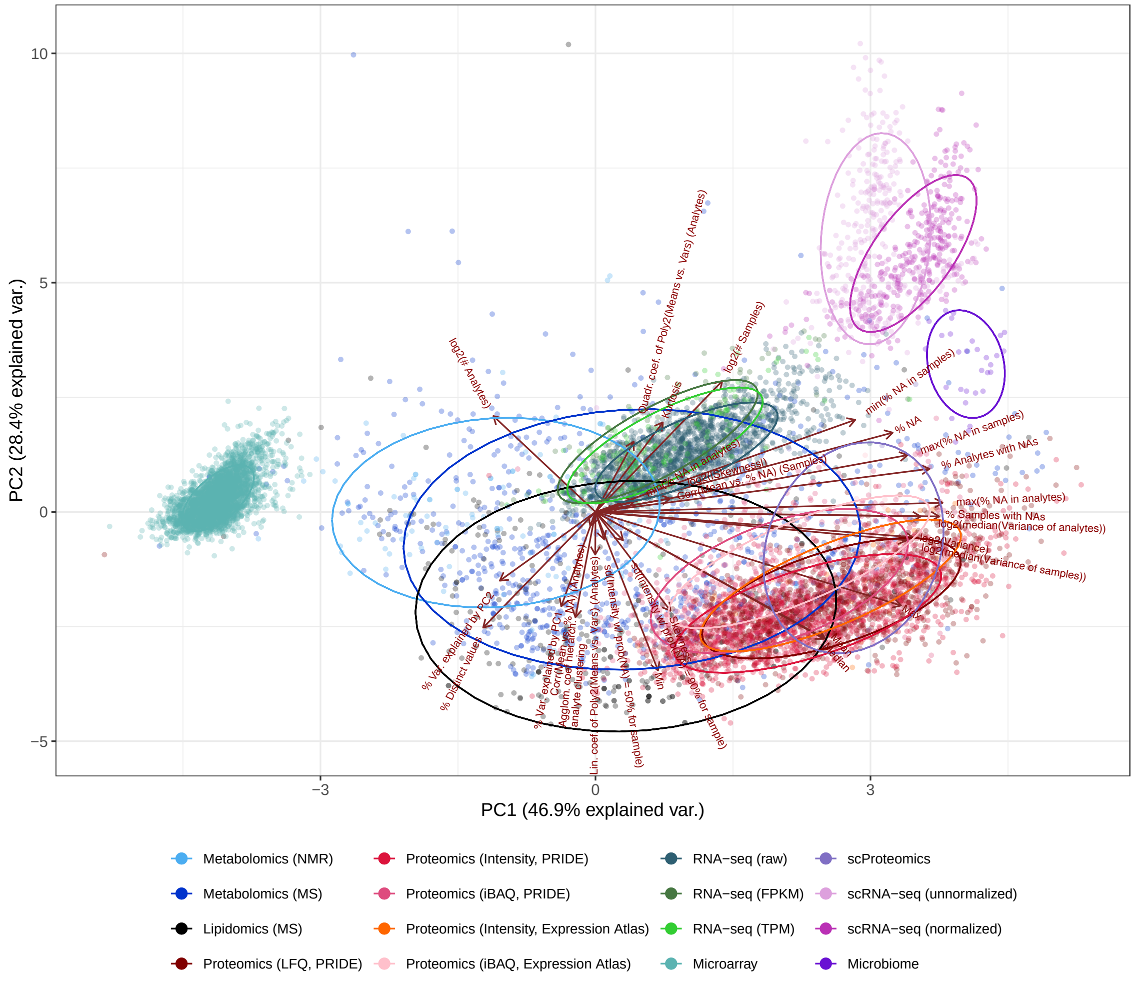

Figure S2. Clustering of the different data types based on their data characteristics, using the nonlinear iterative partial least squares (NIPALS) implementation of principal component analysis (PCA). Arrows indicate the degree of contribution of the various data characteristics to the first two principal components (PC1 and PC2). Ellipses represent the areas capturing 68% of the datasets for each data type. Two lipidomics (MS) datasets and one metabolomics (MS) dataset fall outside the depicted PC2 range and are not shown in this figure.

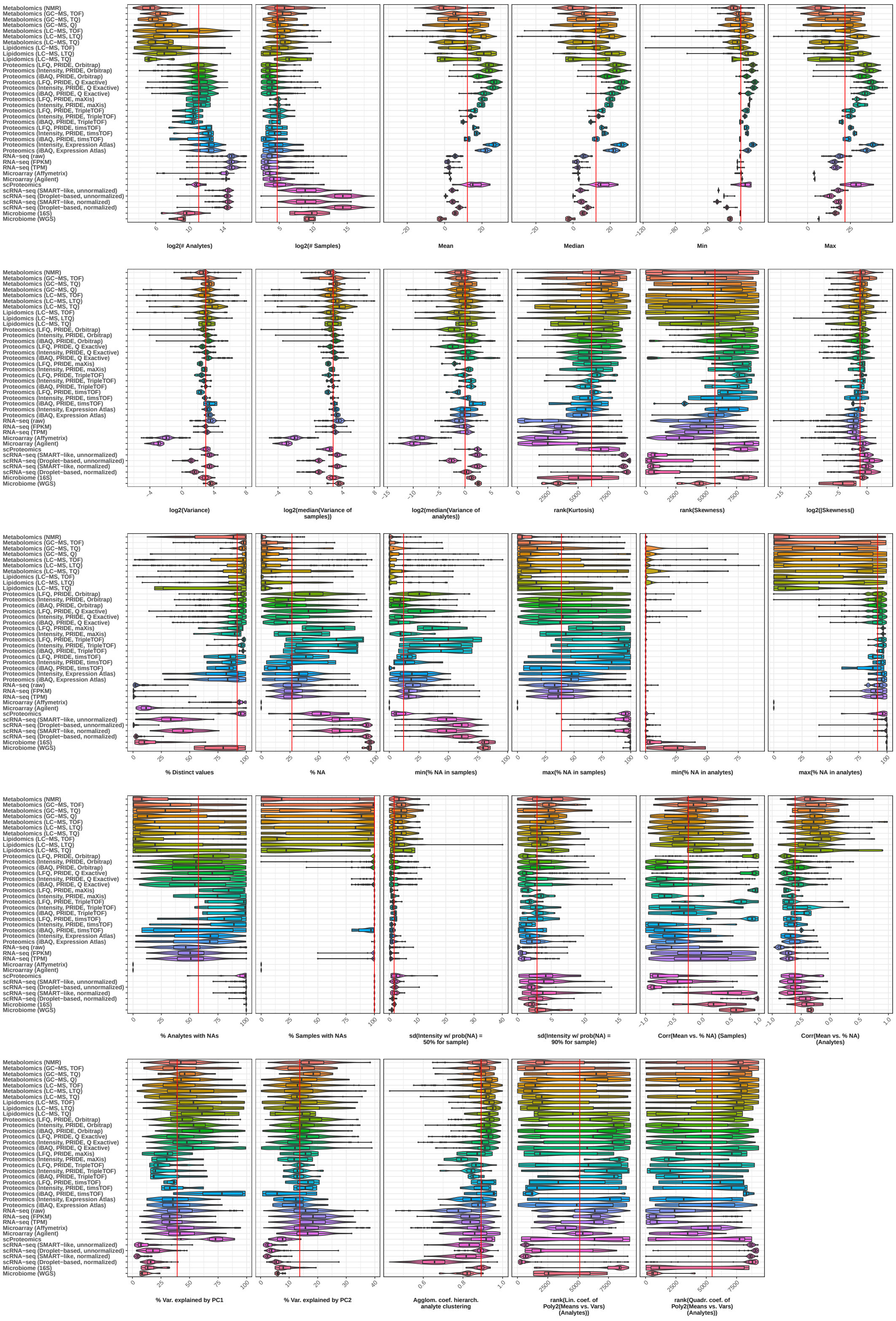

Figure S3. For most investigated data characteristics, substantial differences exist between the data type subgroups. The vertical red lines represent the median of the medians for each data type subgroup. For better comparison, the rank is displayed for ‘Kurtosis’, ‘Skewness’, ‘Lin. coef. of Poly2(Means vs. Vars)(Analytes)’, and ‘Quadr. coef. of Poly2(Means vs. Vars)(Analytes)’.

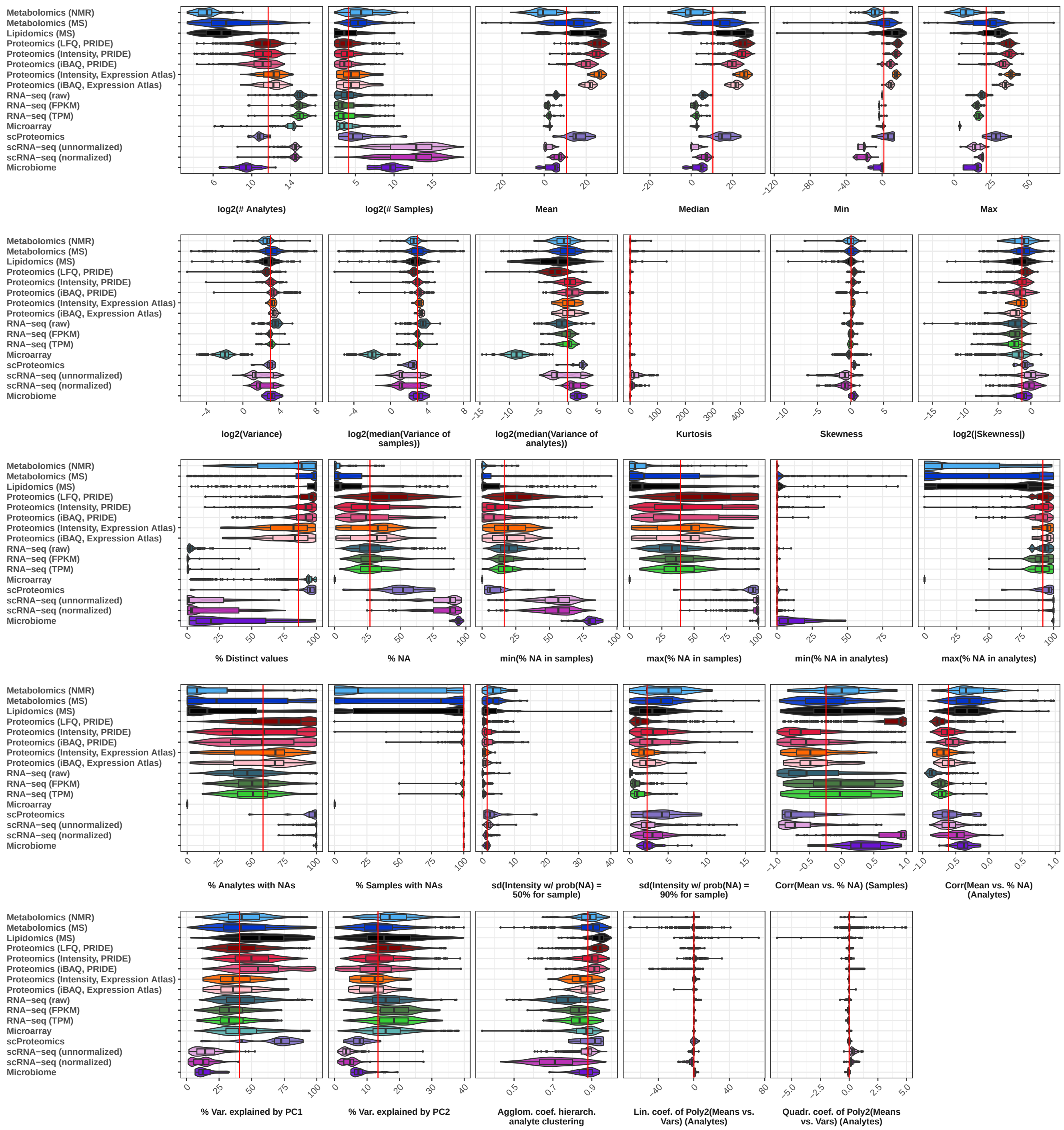

Figure S4. For most investigated data characteristics, substantial differences exist between the data types. The vertical red lines represent the median of the medians for each data type.

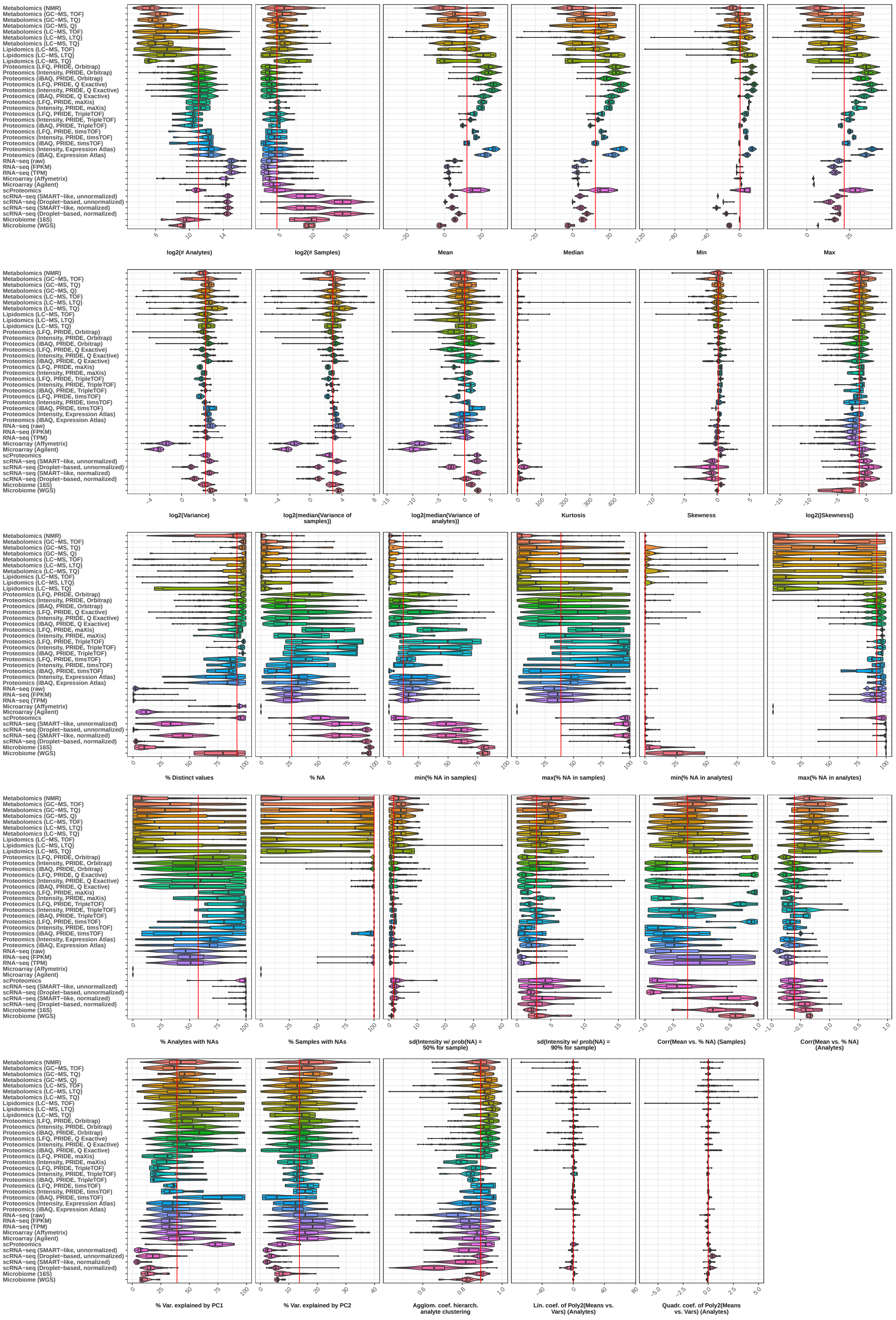

Figure S5. For most investigated data characteristics, substantial differences exist between the data type subgroups. The vertical red lines represent the median of the medians for each data type subgroup.

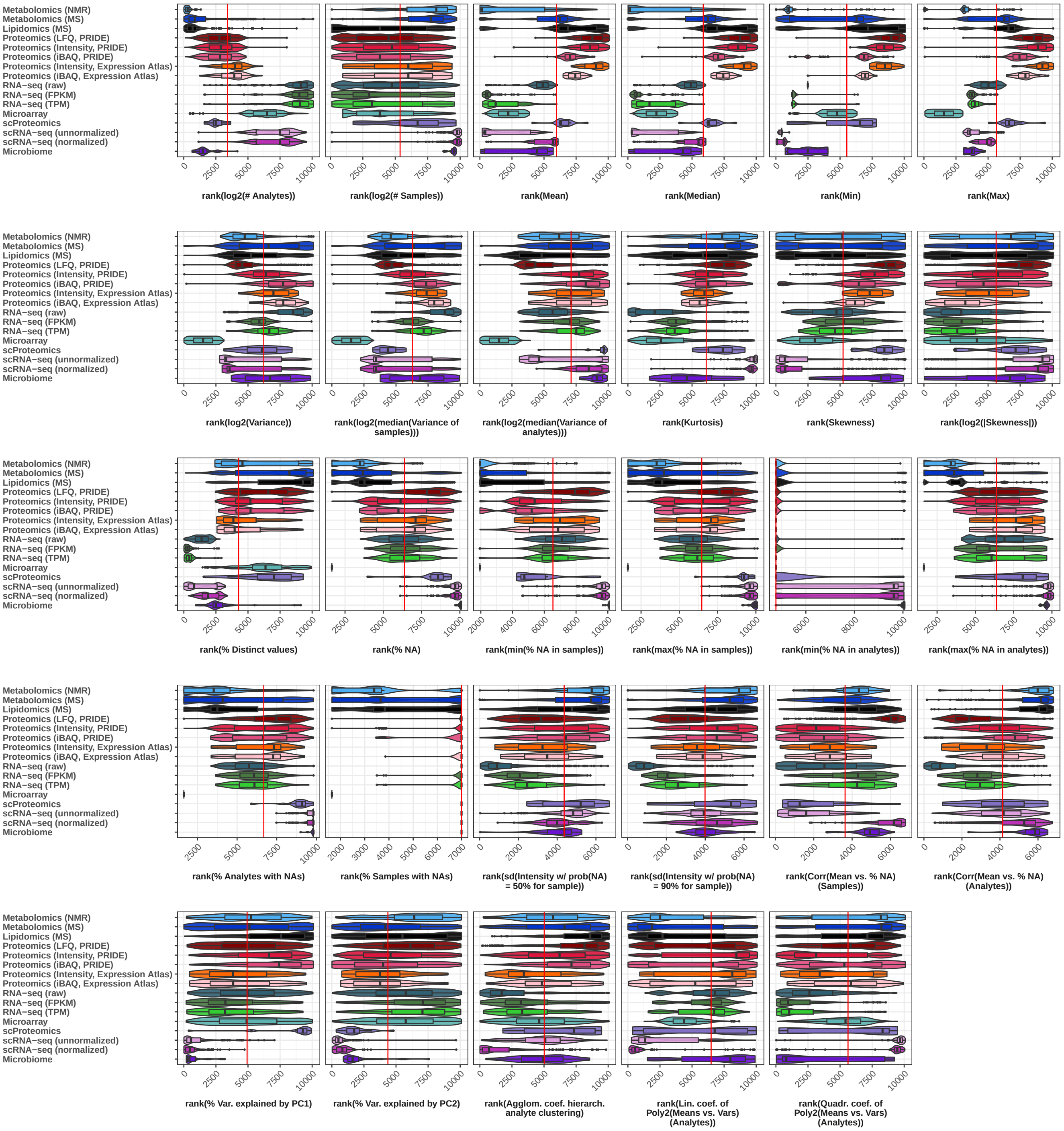

Figure S6. For most investigated data characteristics, substantial differences exist between the data types, with each characteristic ranked separately. The vertical red lines represent the median of the medians for each data type.

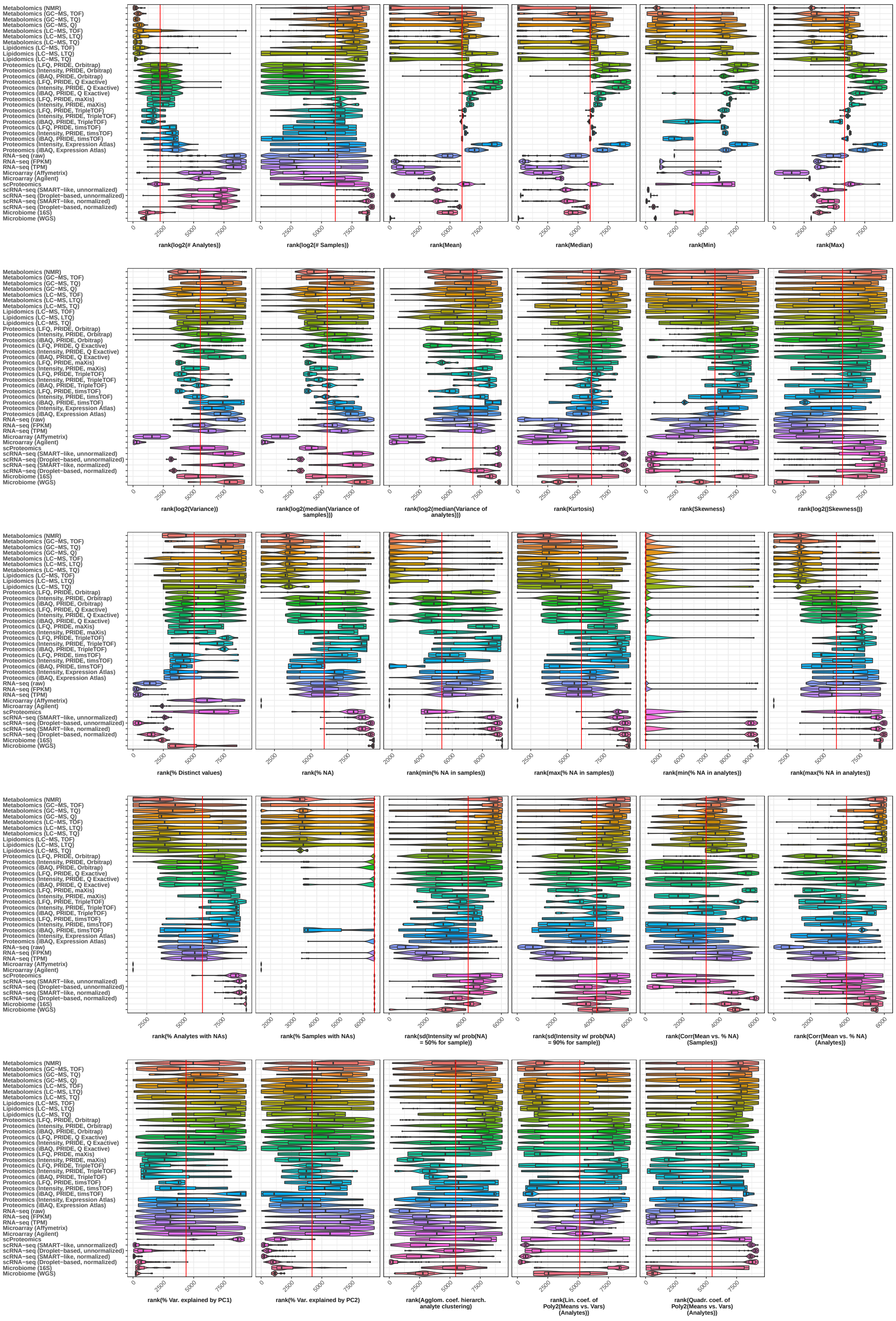

Figure S7. For most investigated data characteristics, substantial differences exist between the data type subgroups,with each characteristic ranked separately. The vertical red lines represent the median of the medians for each data type subgroup.

Table S2. Summary of data types or subgroups exhibiting the lowest and highest values for individual data characteristics, highlighting the most notable trends.

Data characteristic(s)	Lowest	Highest
log2(# Analytes)	Metabolomics, lipidomics	Microarray, RNA-seq (bulk and single-cell)
log2(# Samples)		ScRNA-seq (droplet-based higher than SMART-like), microbiome
Mean, Median	Microbiome (WGS)	Lipidomics, metabolomics (MS), proteomics (bulk and single-cell)
Min	ScRNA-seq	Lipidomics, metabolomics (MS), proteomics (bulk and single-cell)
Max	Metabolomics (NMR), microarray	Lipidomics, metabolomics (MS), proteomics (bulk and single-cell)
log2(Variance), log2(median(Variance of samples)), log2(median(Variance of analytes))	Microarray (Agilent smaller than Affymetrix)	
Kurtosis		ScRNA-seq
log2(|Skewness|)	Microbiome (WGS)	ScRNA-seq data (left-skewed)
% Distinct values	RNA-seq data (bulk and single-cell, droplet-based lower than SMART-like), microarray (Agilent, high for microarray(Affymetrix)), microbiome (16S)	
% NA, min(% NA in samples)	Microarray	ScRNA-seq (droplet-based higher than SMART-like), microbiome
max(% NA in samples)	Microarray	ScRNA-seq, microbiome, scProteomics
min(% NA in analytes)		Microbiome
max(% NA in analytes)	Microarray	ScRNA-seq, microbiome
% Analytes with NAs	Microarray	ScRNA-seq, microbiome, scProteomics
% Samples with NAs	Microarray	
sd(Intensity w/ prob(NA) = 50% for sample), sd(Intensity w/ prob(NA) = 90% for sample)	RNA-seq (bulk)	Metabolomics, lipidomics, scProteomics
Corr(Mean vs. % NA) (Samples)	ScRNA-seq (Droplet-based, unnormalized) (negative correlation)	Proteomics (LFQ, PRIDE), scRNA-seq (Droplet-based, normalized) (both showing a positive correlation)
Corr(Mean vs. % NA) (Analytes)	RNA-seq (raw)	Metabolomics, lipidomics
% Var. explained by PC1	ScRNA-seq, microbiome	ScProteomics
% Var. explained by PC2	ScProteomics, scRNA-seq, microbiome	
Agglom. coef. hierarch. analyte clustering	ScRNA-seq (Droplet-based, normalized)	
Lin. coef. of Poly2(Means vs. Vars) (Analytes)	ScRNA-seq	
Quadr. coef. of Poly2(Means vs. Vars) (Analytes)		ScRNA-seq

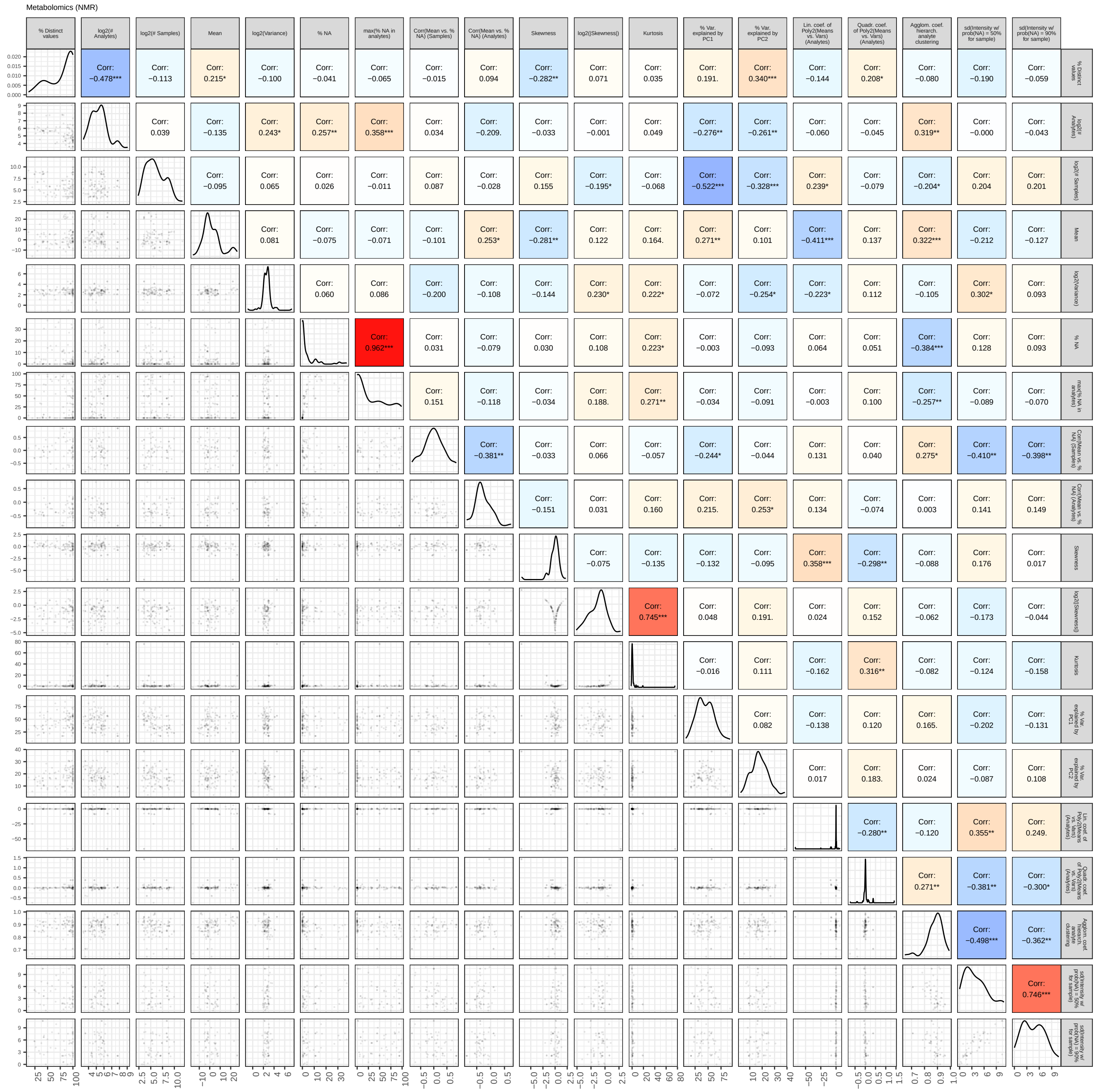

Figure S8. Spearman correlation plot of selected data characteristics across Metabolomics nuclear magnetic resonance (NMR) datasets. In the upper right corner the Spearman correlation coefficients for pairs of data characteristics are displayed, where *** = p<0.001, ** = p<0.01, * = p<0.05, and . = p<0.1. The intensity of red and blue corresponds to the strength of the positive and negative correlations, respectively. In the bottom left corner, scatter plots of pairs of data characteristics are shown, with each data point representing a dataset. On the diagonal, the distribution of each data characteristic is displayed.

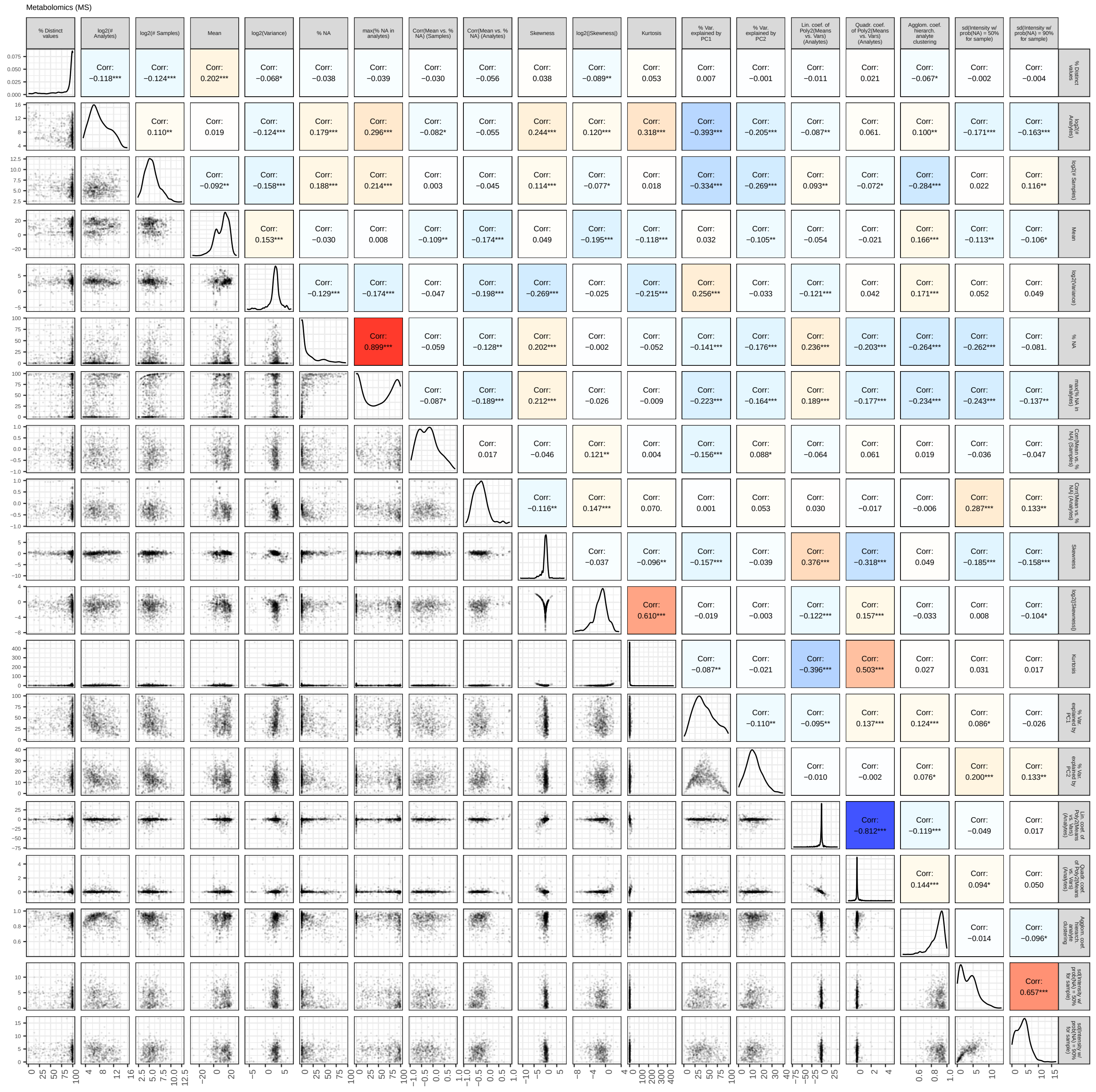

Figure S9. Spearman correlation plot of selected data characteristics across Metabolomics mass spectrometry (MS) datasets. In the upper right corner the Spearman correlation coefficients for pairs of data characteristics are displayed, where *** = p<0.001, ** = p<0.01, * = p<0.05, and . = p<0.1. The intensity of red and blue corresponds to the strength of the positive and negative correlations, respectively. In the bottom left corner, scatter plots of pairs of data characteristics are shown, with each data point representing a dataset. On the diagonal, the distribution of each data characteristic is displayed.

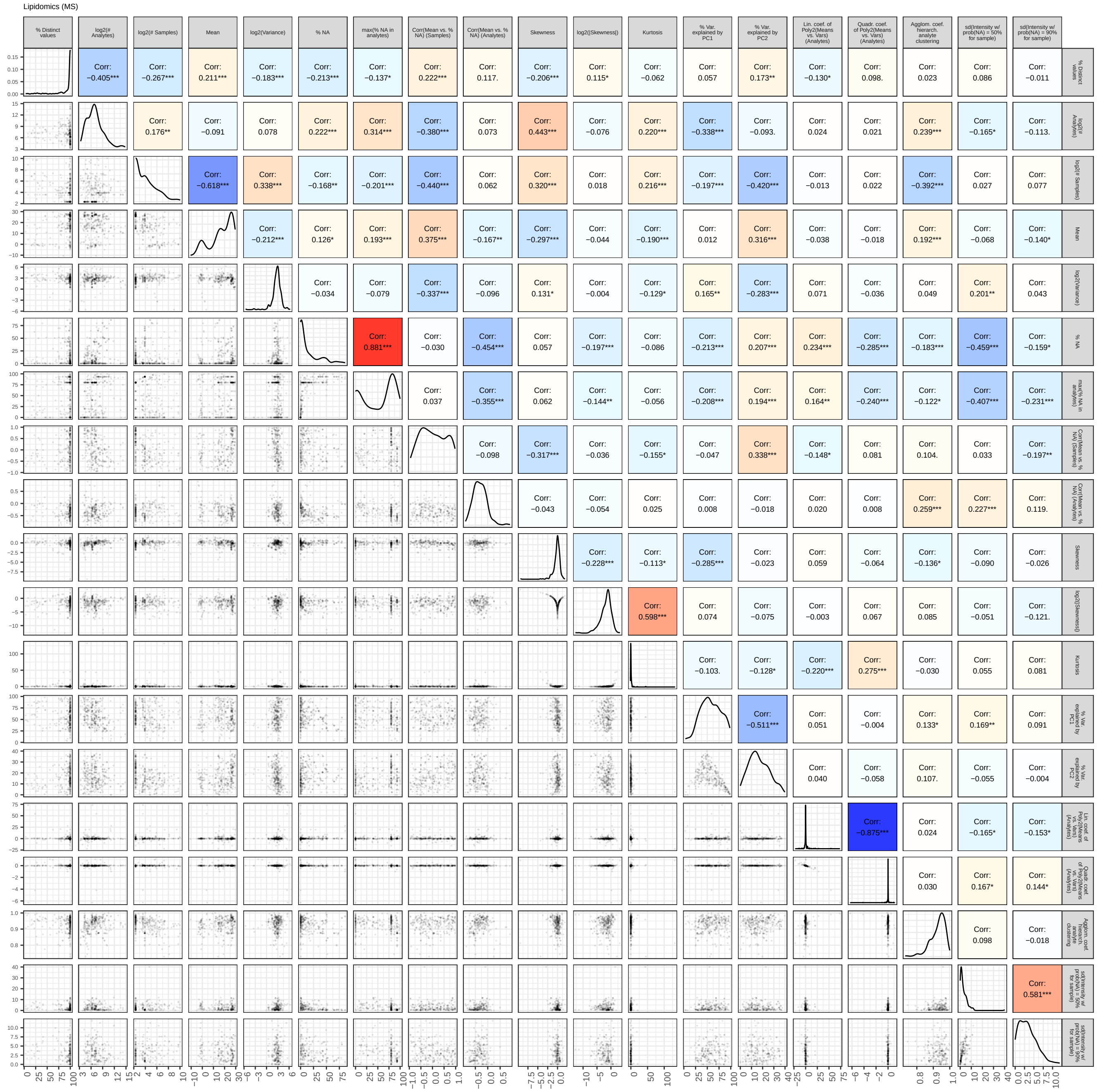

Figure S10. Spearman correlation plot of selected data characteristics across Lipidomics mass spectrometry (MS) datasets. In the upper right corner the Spearman correlation coefficients for pairs of data characteristics are displayed, where *** = p<0.001, ** = p<0.01, * = p<0.05, and . = p<0.1. The intensity of red and blue corresponds to the strength of the positive and negative correlations, respectively. In the bottom left corner, scatter plots of pairs of data characteristics are shown, with each data point representing a dataset. On the diagonal, the distribution of each data characteristic is displayed.

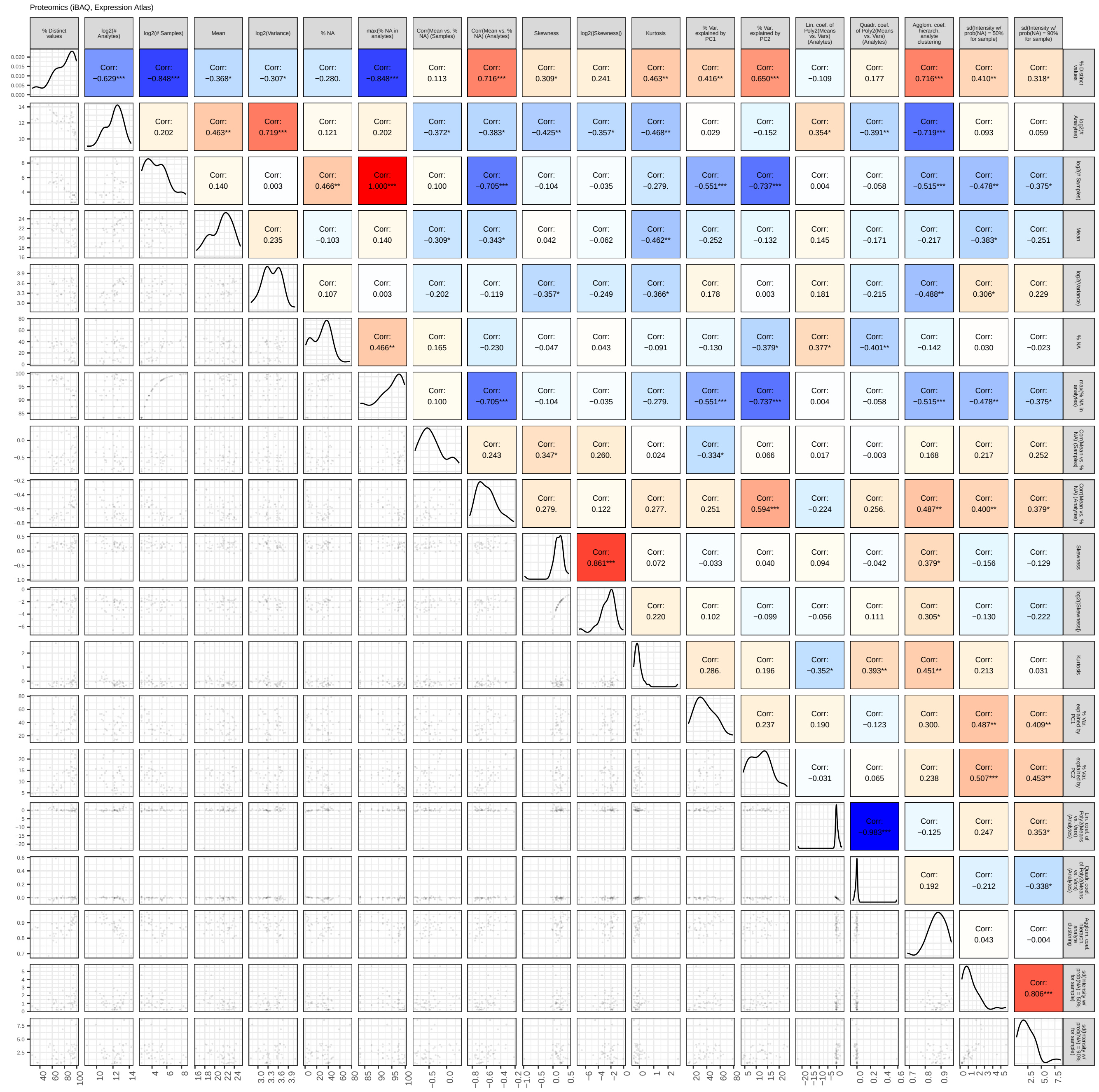

Figure S11. Spearman correlation plot of selected data characteristics across Proteomics (iBAQ, Expression Atlas) datasets. In the upper right corner the Spearman correlation coefficients for pairs of data characteristics are displayed, where *** = p<0.001, ** = p<0.01, * = p<0.05, and . = p<0.1. The intensity of red and blue corresponds to the strength of the positive and negative correlations, respectively. In the bottom left corner, scatter plots of pairs of data characteristics are shown, with each data point representing a dataset. On the diagonal, the distribution of each data characteristic is displayed.

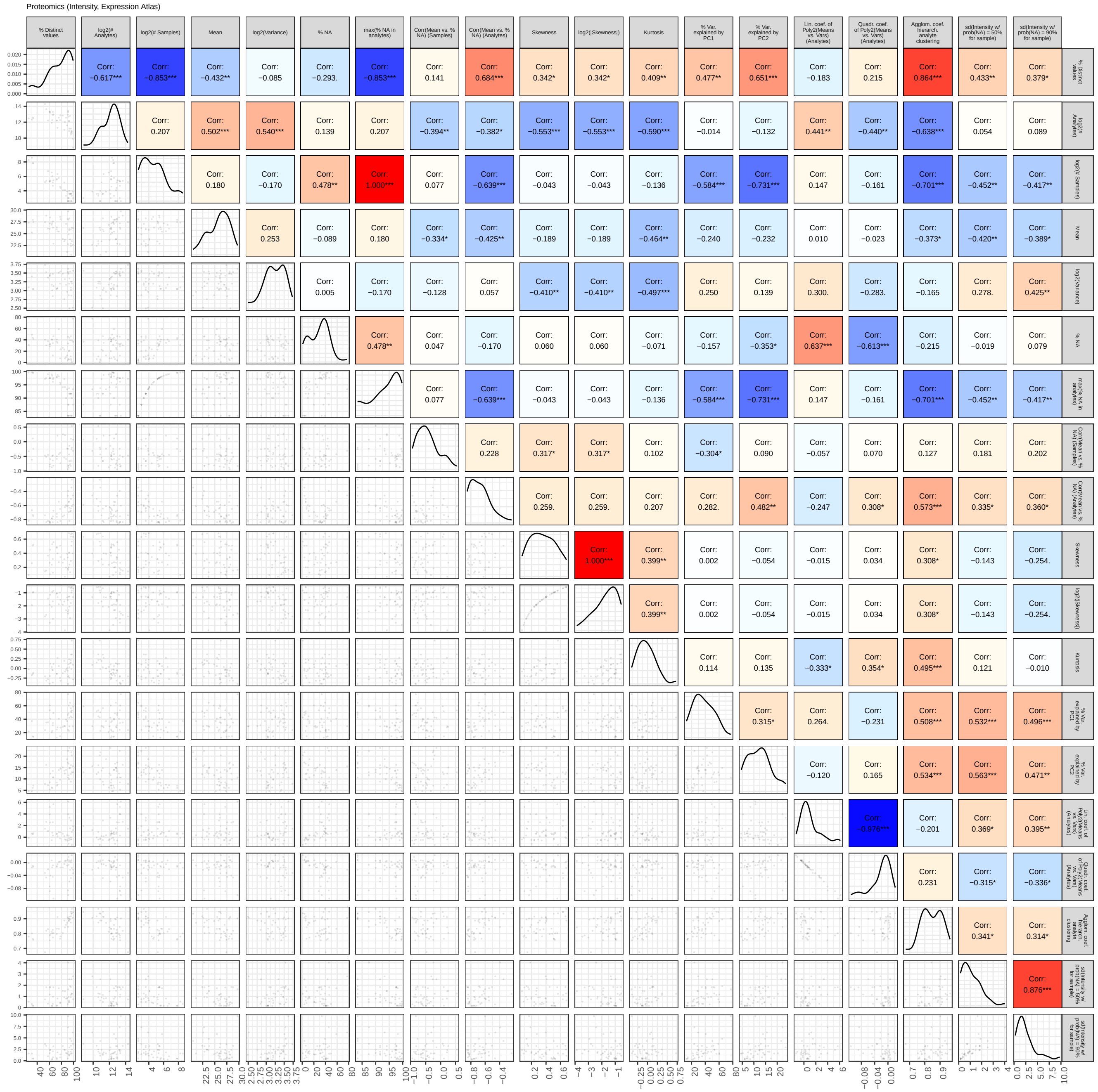

Figure S12. Spearman correlation plot of selected data characteristics across Proteomics (Intensity, Expression Atlas) datasets. In the upper right corner the Spearman correlation coefficients for pairs of data characteristics are displayed, where *** = p<0.001, ** = p<0.01, * = p<0.05, and . = p<0.1. The intensity of red and blue corresponds to the strength of the positive and negative correlations, respectively. In the bottom left corner, scatter plots of pairs of data characteristics are shown, with each data point representing a dataset. On the diagonal, the distribution of each data characteristic is displayed.

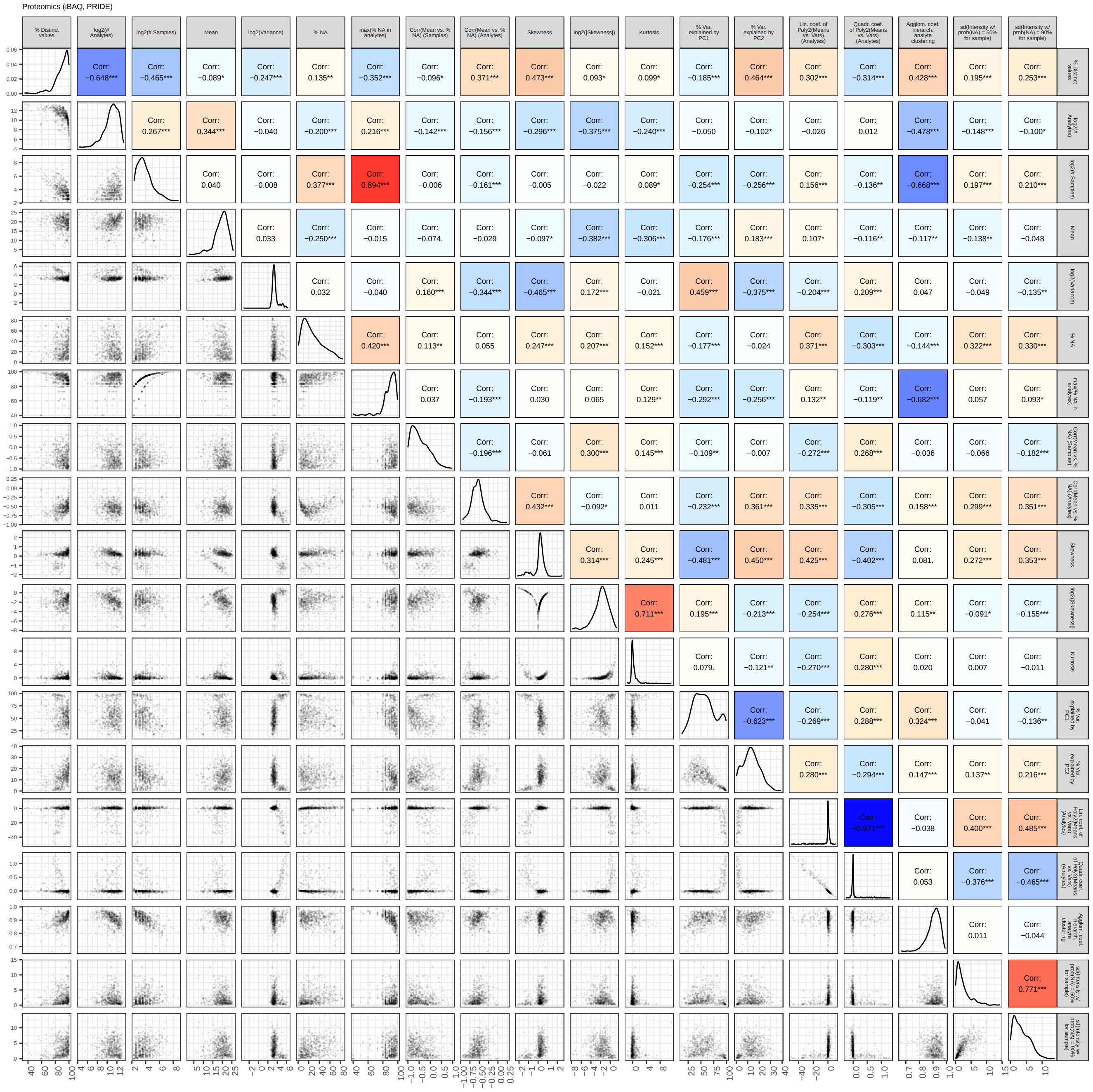

Figure S13. Spearman correlation plot of selected data characteristics across Proteomics (iBAQ, PRIDE) datasets. In the upper right corner the Spearman correlation coefficients for pairs of data characteristics are displayed, where *** = p<0.001, ** = p<0.01, * = p<0.05, and . = p<0.1. The intensity of red and blue corresponds to the strength of the positive and negative correlations, respectively. In the bottom left corner, scatter plots of pairs of data characteristics are shown, with each data point representing a dataset. On the diagonal, the distribution of each data characteristic is displayed.

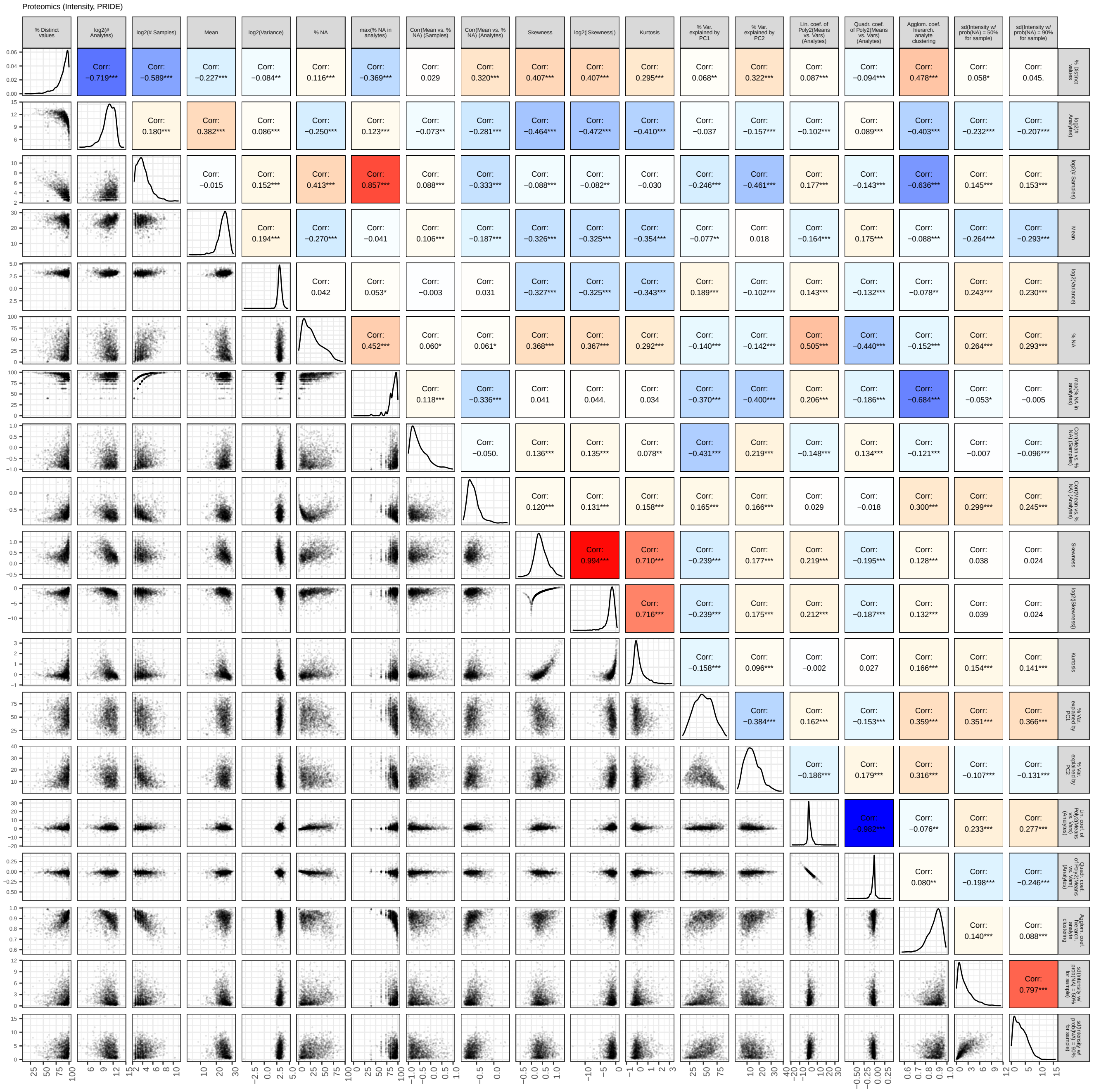

Figure S14. Spearman correlation plot of selected data characteristics across Proteomics (Intensity, PRIDE) datasets. In the upper right corner the Spearman correlation coefficients for pairs of data characteristics are displayed, where *** = $p<0.001$, ** = $p<0.01$, * = $p<0.05$, and . = $p<0.1$. The intensity of red and blue corresponds to the strength of the positive and negative correlations, respectively. In the bottom left corner, scatter plots of pairs of data characteristics are shown, with each data point representing a dataset. On the diagonal, the distribution of each data characteristic is displayed.

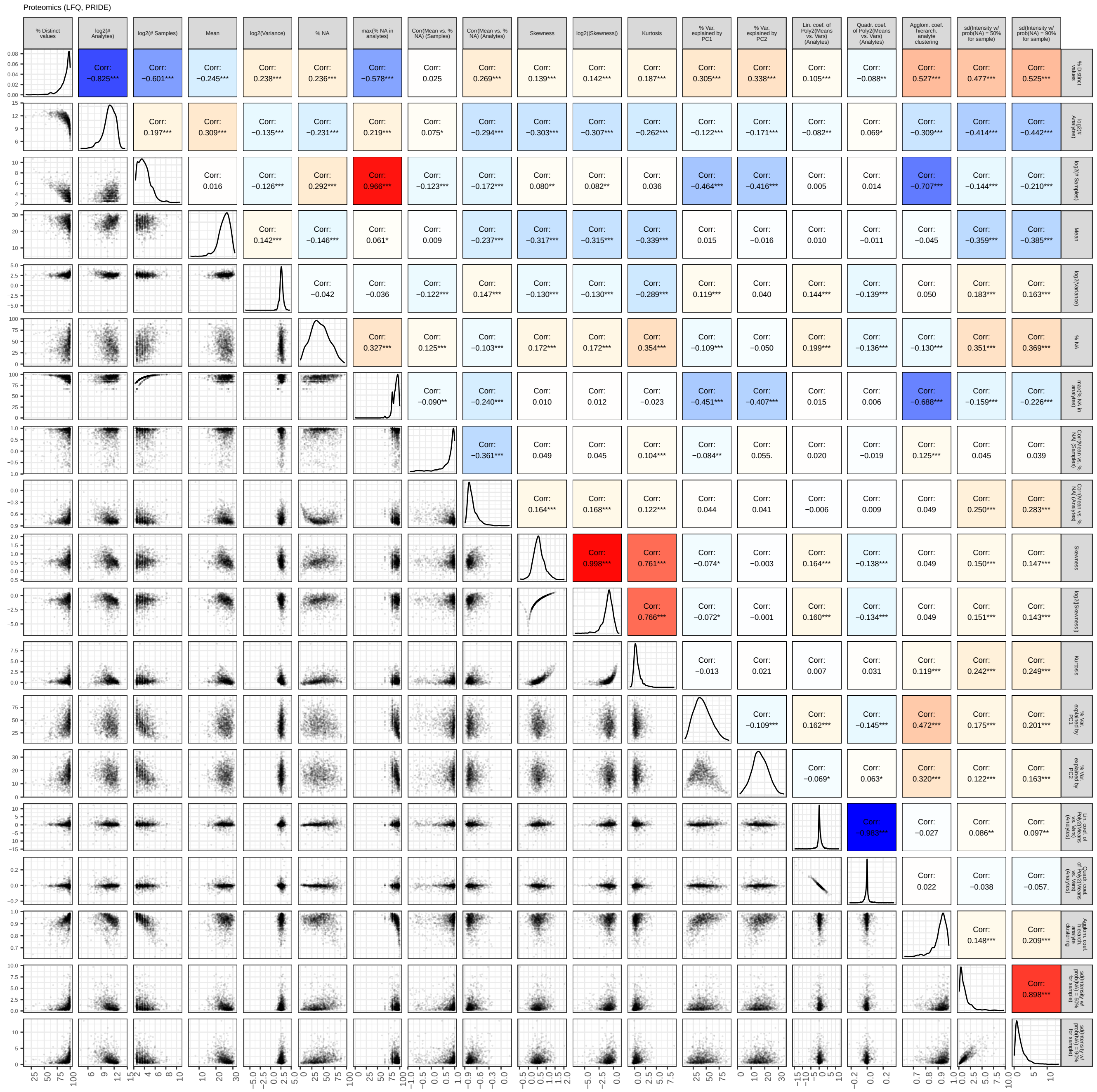

Figure S15. Spearman correlation plot of selected data characteristics across Proteomics (LFQ, PRIDE) datasets. In the upper right corner the Spearman correlation coefficients for pairs of data characteristics are displayed, where *** = p<0.001, ** = p<0.01, * = p<0.05, and . = p<0.1. The intensity of red and blue corresponds to the strength of the positive and negative correlations, respectively. In the bottom left corner, scatter plots of pairs of data characteristics are shown, with each data point representing a dataset. On the diagonal, the distribution of each data characteristic is displayed.

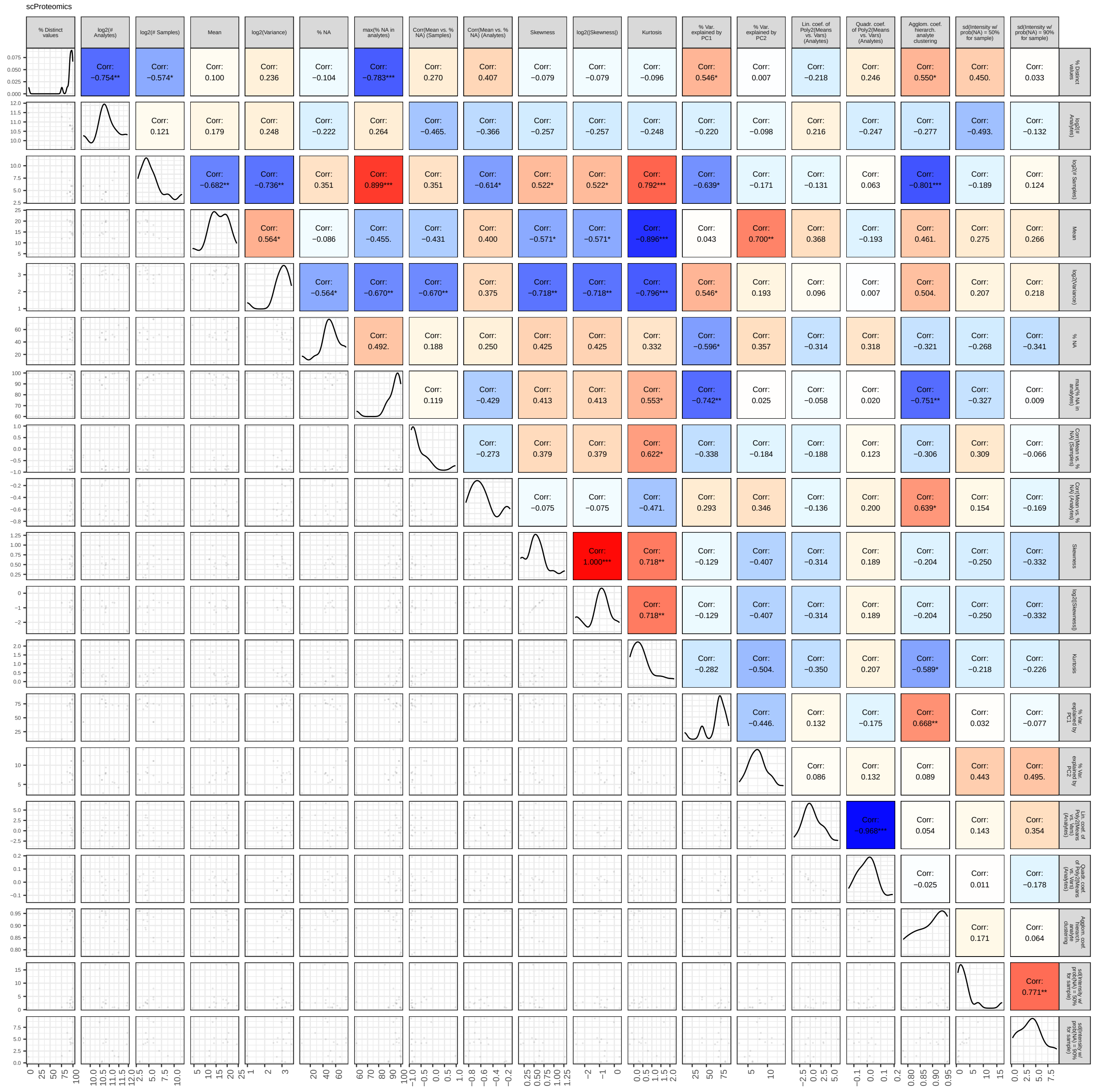

Figure S16. Spearman correlation plot of selected data characteristics across scProteomics datasets. In the upper right corner the Spearman correlation coefficients for pairs of data characteristics are displayed, where *** = p<0.001, ** = p<0.01, * = p<0.05, and . = p<0.1. The intensity of red and blue corresponds to the strength of the positive and negative correlations, respectively. In the bottom left corner, scatter plots of pairs of data characteristics are shown, with each data point representing a dataset. On the diagonal, the distribution of each data characteristic is displayed.

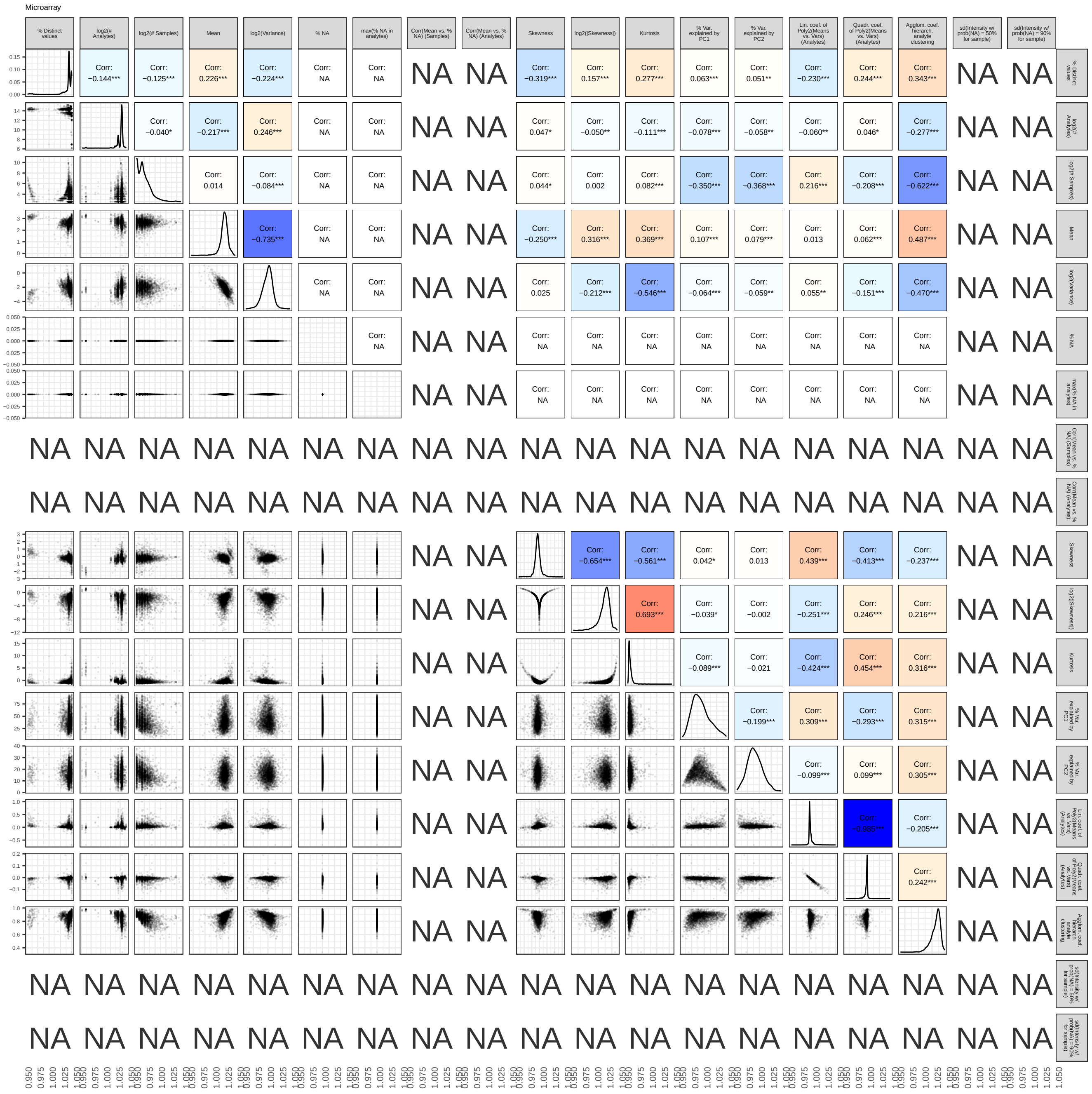

Figure S17. Spearman correlation plot of selected data characteristics across Microarray datasets. In the upper right corner the Spearman correlation coefficients for pairs of data characteristics are displayed, where *** = p<0.001, ** = p<0.01, * = p<0.05, and . = p<0.1. The intensity of red and blue corresponds to the strength of the positive and negative correlations, respectively. In the bottom left corner, scatter plots of pairs of data characteristics are shown, with each data point representing a dataset. On the diagonal, the distribution of each data characteristic is displayed. As microarray data does not contain missing values, no correlations could be calculated for data characteristics related to missing values, indicated by 'NA'.

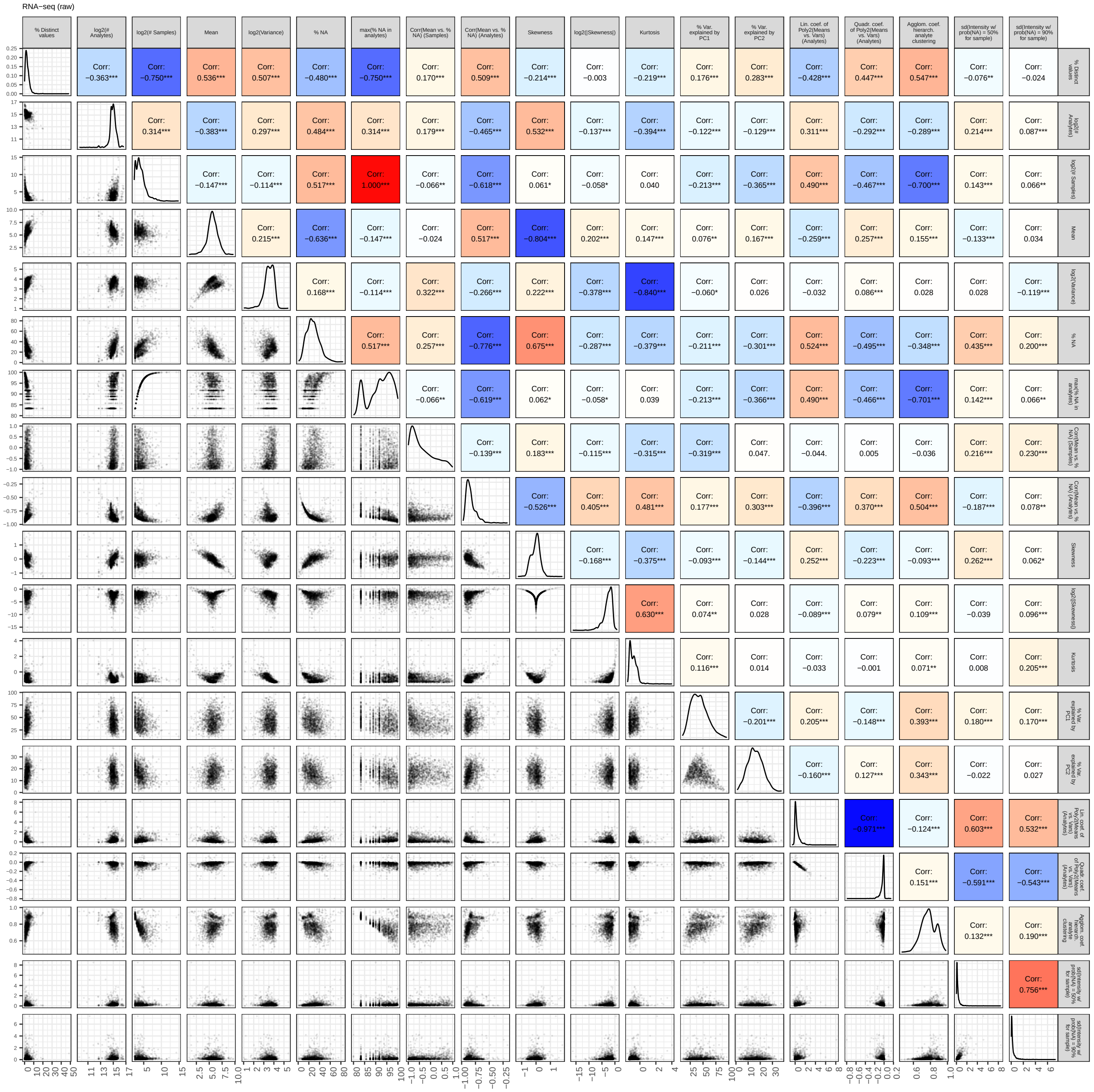

Figure S18. Spearman correlation plot of selected data characteristics across RNA-seq (raw) datasets. In the upper right corner the Spearman correlation coefficients for pairs of data characteristics are displayed, where *** = p<0.001, ** = p<0.01, * = p<0.05, and . = p<0.1. The intensity of red and blue corresponds to the strength of the positive and negative correlations, respectively. In the bottom left corner, scatter plots of pairs of data characteristics are shown, with each data point representing a dataset. On the diagonal, the distribution of each data characteristic is displayed.

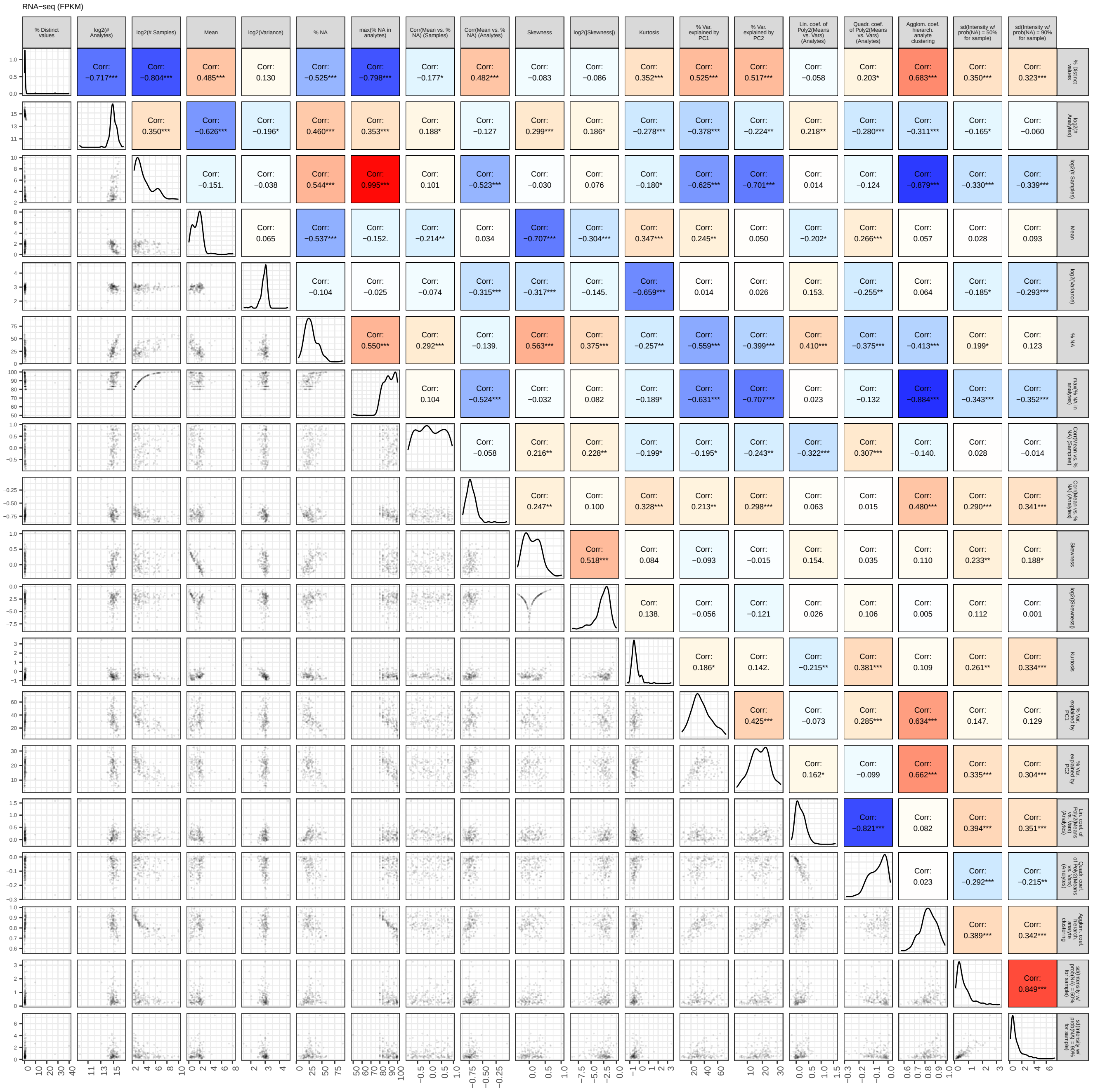

Figure S19. Spearman correlation plot of selected data characteristics across RNA-seq (FPKM) datasets. In the upper right corner the Spearman correlation coefficients for pairs of data characteristics are displayed, where *** = p<0.001, ** = p<0.01, * = p<0.05, and . = p<0.1. The intensity of red and blue corresponds to the strength of the positive and negative correlations, respectively. In the bottom left corner, scatter plots of pairs of data characteristics are shown, with each data point representing a dataset. On the diagonal, the distribution of each data characteristic is displayed.

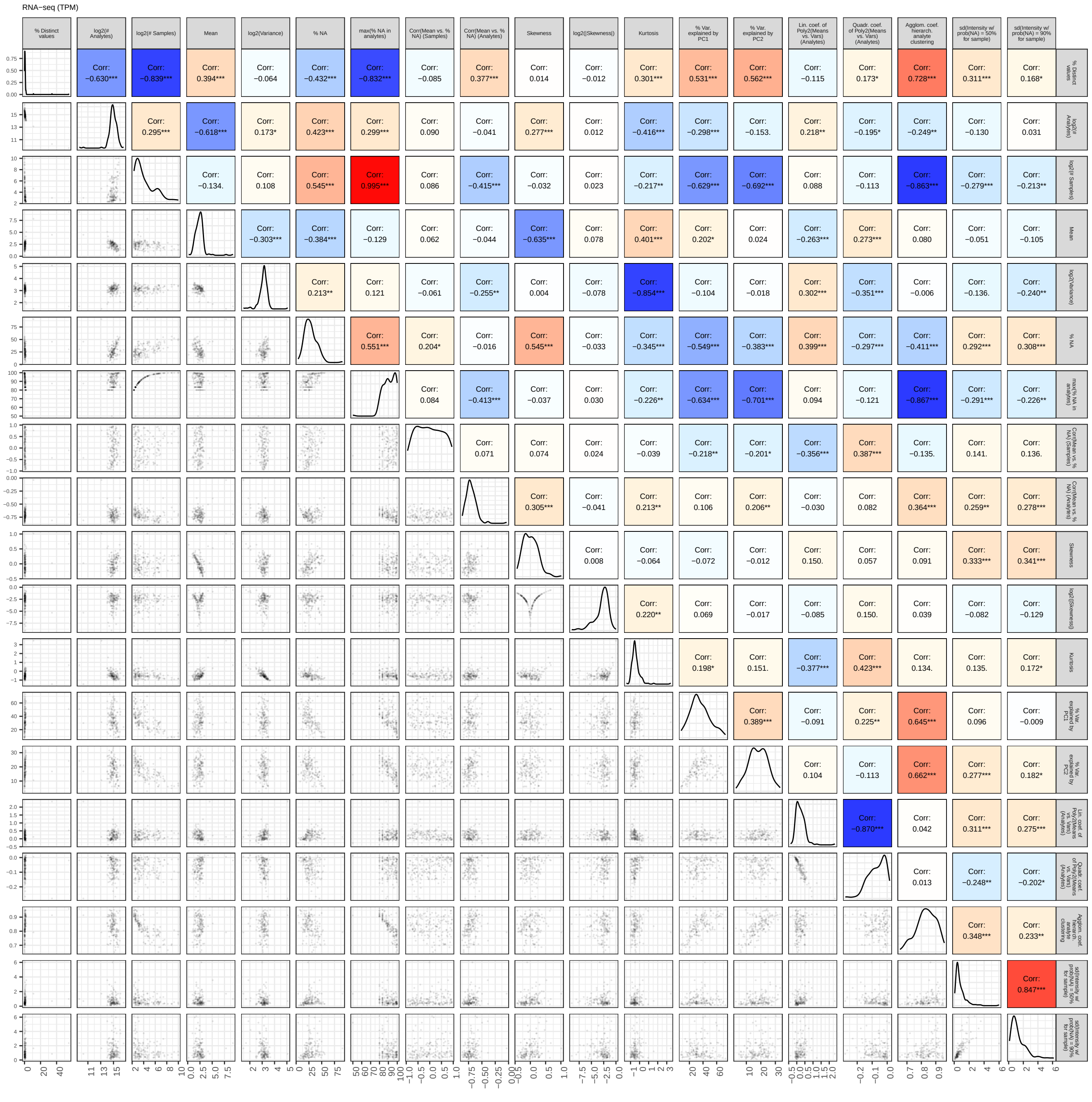

Figure S20. Spearman correlation plot of selected data characteristics across RNA-seq (TPM) datasets. In the upper right corner the Spearman correlation coefficients for pairs of data characteristics are displayed, where *** = p<0.001, ** = p<0.01, * = p<0.05, and . = p<0.1. The intensity of red and blue corresponds to the strength of the positive and negative correlations, respectively. In the bottom left corner, scatter plots of pairs of data characteristics are shown, with each data point representing a dataset. On the diagonal, the distribution of each data characteristic is displayed.

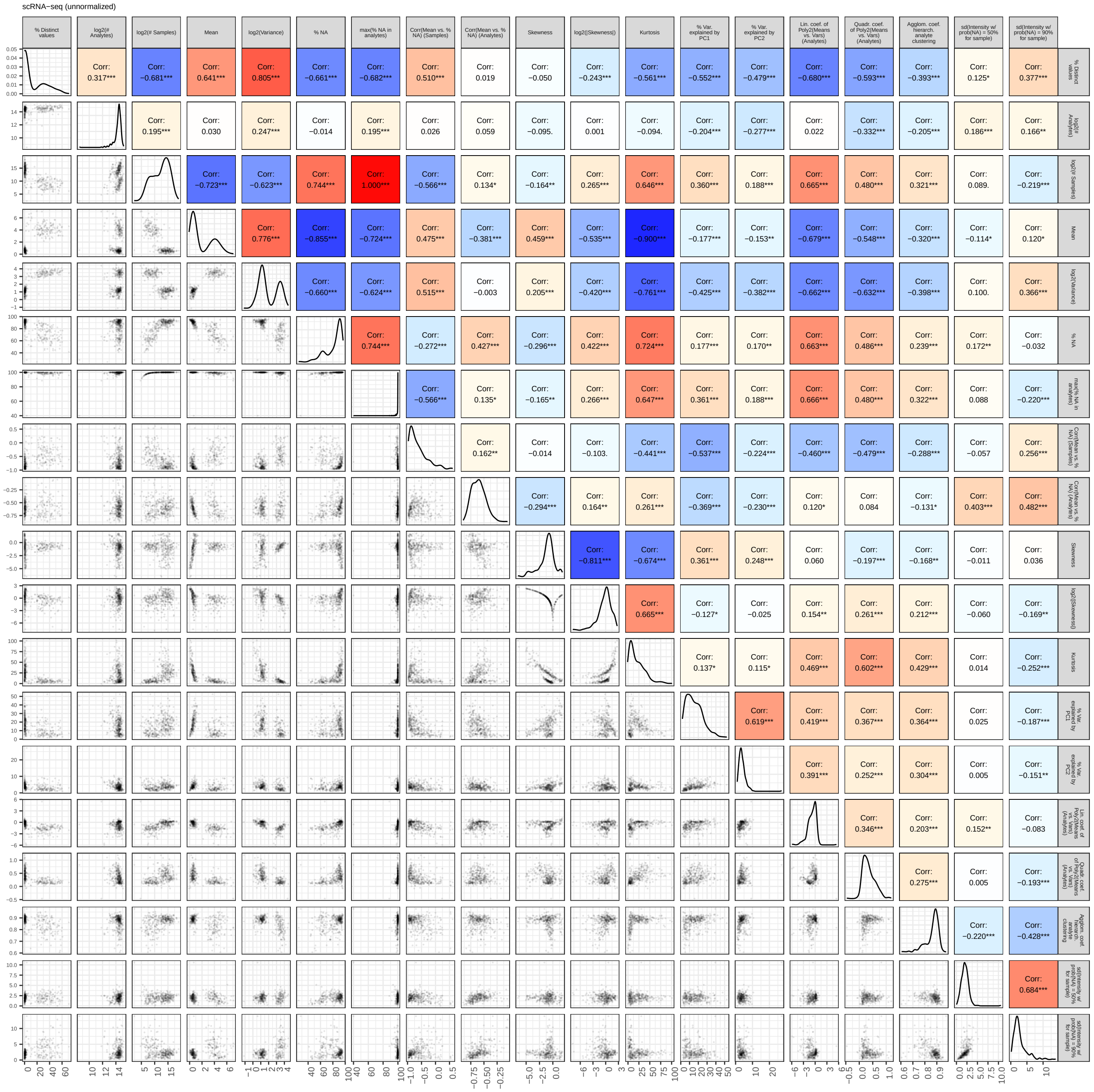

Figure S21. Spearman correlation plot of selected data characteristics across scRNA-seq (unnormalized) datasets. In the upper right corner the Spearman correlation coefficients for pairs of data characteristics are displayed, where *** = p<0.001, ** = p<0.01, * = p<0.05, and . = p<0.1. The intensity of red and blue corresponds to the strength of the positive and negative correlations, respectively. In the bottom left corner, scatter plots of pairs of data characteristics are shown, with each data point representing a dataset. On the diagonal, the distribution of each data characteristic is displayed.

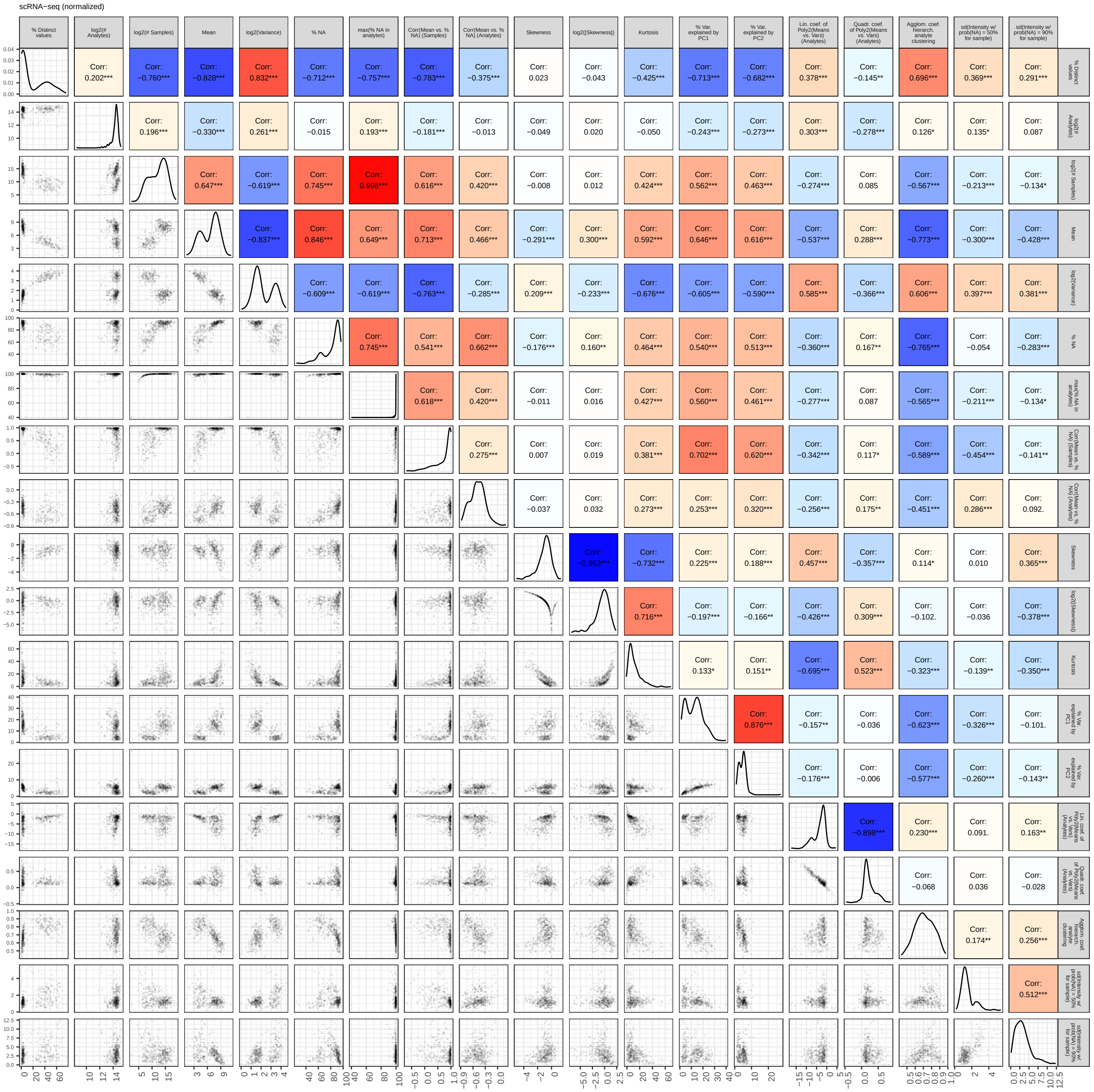

Figure S22. Spearman correlation plot of selected data characteristics across scRNA-seq (normalized) datasets. In the upper right corner the Spearman correlation coefficients for pairs of data characteristics are displayed, where *** = p<0.001, ** = p<0.01, * = p<0.05, and . = p<0.1. The intensity of red and blue corresponds to the strength of the positive and negative correlations, respectively. In the bottom left corner, scatter plots of pairs of data characteristics are shown, with each data point representing a dataset. On the diagonal, the distribution of each data characteristic is displayed.

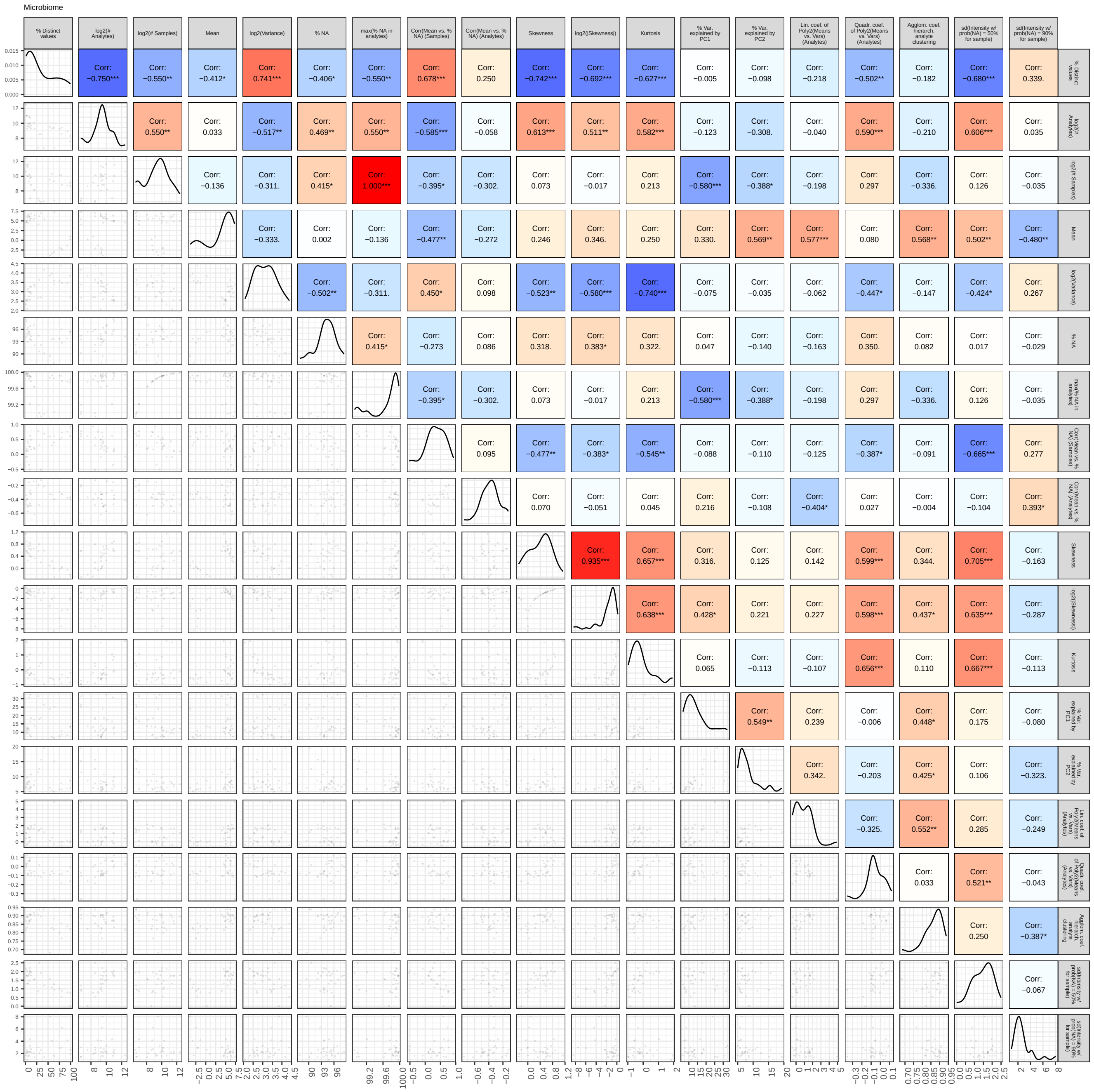

Figure S23. Spearman correlation plot of selected data characteristics across Microbiome datasets. In the upper right corner the Spearman correlation coefficients for pairs of data characteristics are displayed, where *** = p<0.001, ** = p<0.01, * = p<0.05, and . = p<0.1. The intensity of red and blue corresponds to the strength of the positive and negative correlations, respectively. In the bottom left corner, scatter plots of pairs of data characteristics are shown, with each data point representing a dataset. On the diagonal, the distribution of each data characteristic is displayed.

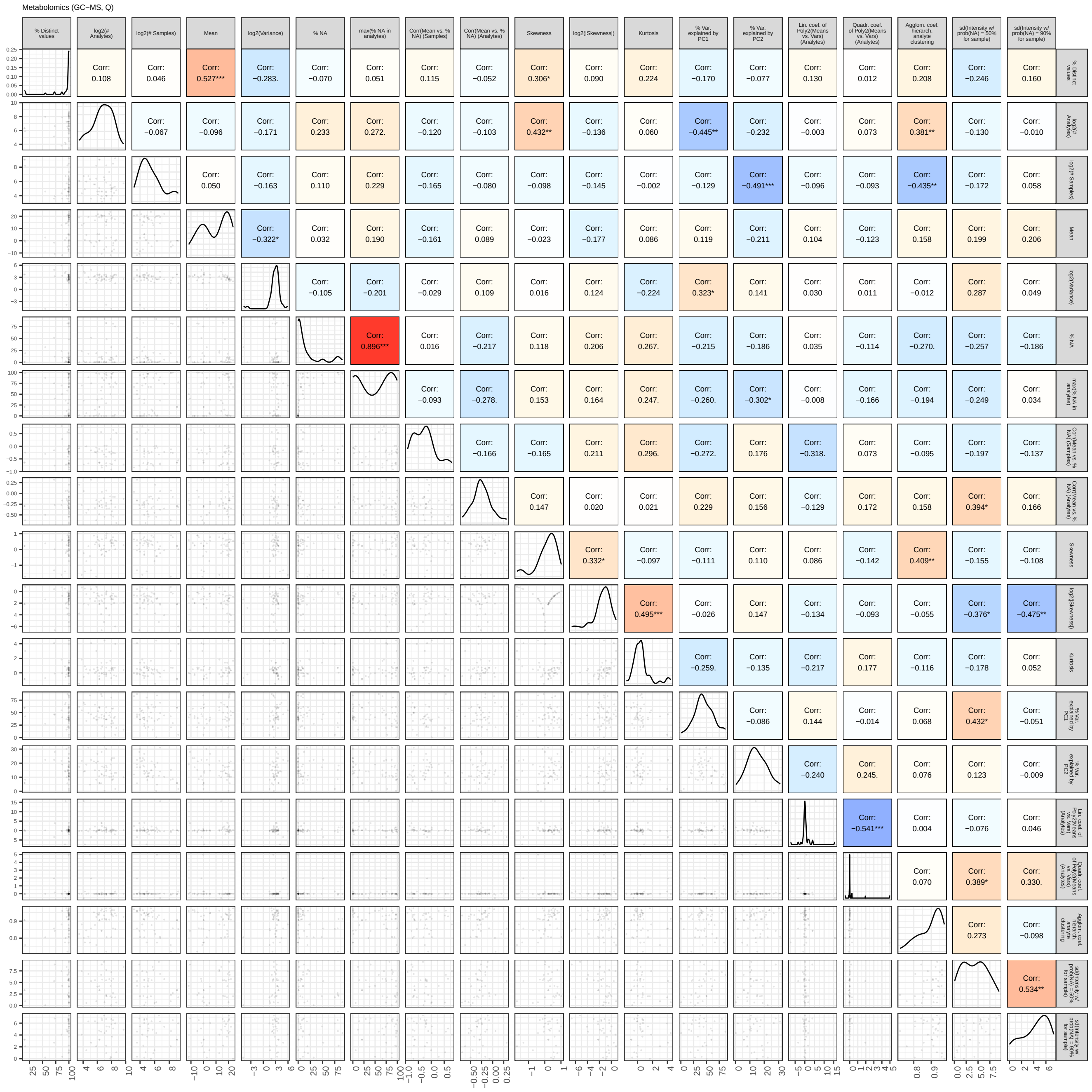

Figure S24. Spearman correlation plot of selected data characteristics across Metabolomics gas chromatography (GC)-quadrupole (Q) mass spectrometry (MS) datasets. In the upper right corner the Spearman correlation coefficients for pairs of data characteristics are displayed, where *** = p<0.001, ** = p<0.01, * = p<0.05, and . = p<0.1. The intensity of red and blue corresponds to the strength of the positive and negative correlations, respectively. In the bottom left corner, scatter plots of pairs of data characteristics are shown, with each data point representing a dataset. On the diagonal, the distribution of each data characteristic is displayed.

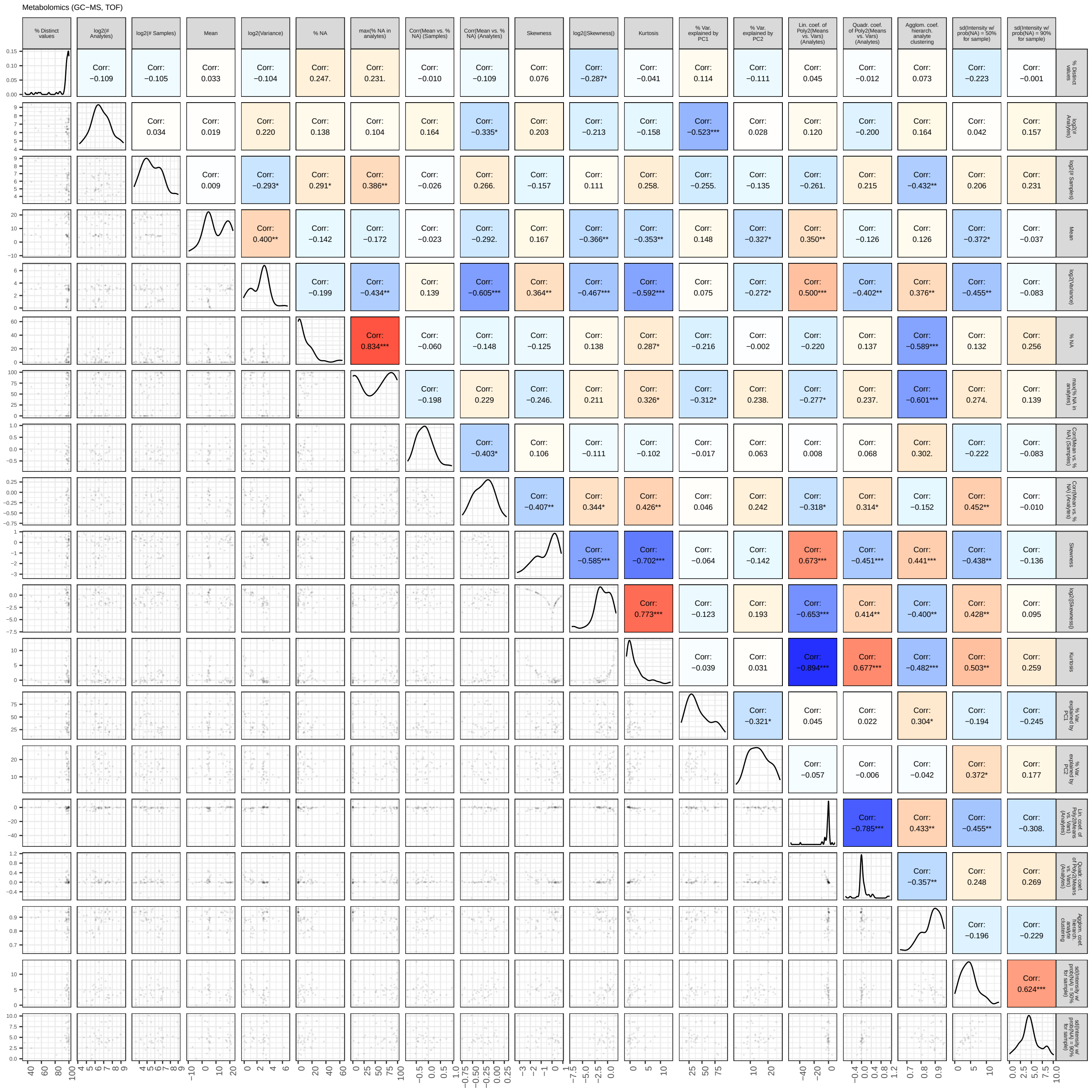

Figure S25. Spearman correlation plot of selected data characteristics across Metabolomics gas chromatography (GC)-time-of-flight (TOF) mass spectrometry MS datasets. In the upper right corner the Spearman correlation coefficients for pairs of data characteristics are displayed, where *** = p<0.001, ** = p<0.01, * = p<0.05, and . = p<0.1. The intensity of red and blue corresponds to the strength of the positive and negative correlations, respectively. In the bottom left corner, scatter plots of pairs of data characteristics are shown, with each data point representing a dataset. On the diagonal, the distribution of each data characteristic is displayed.

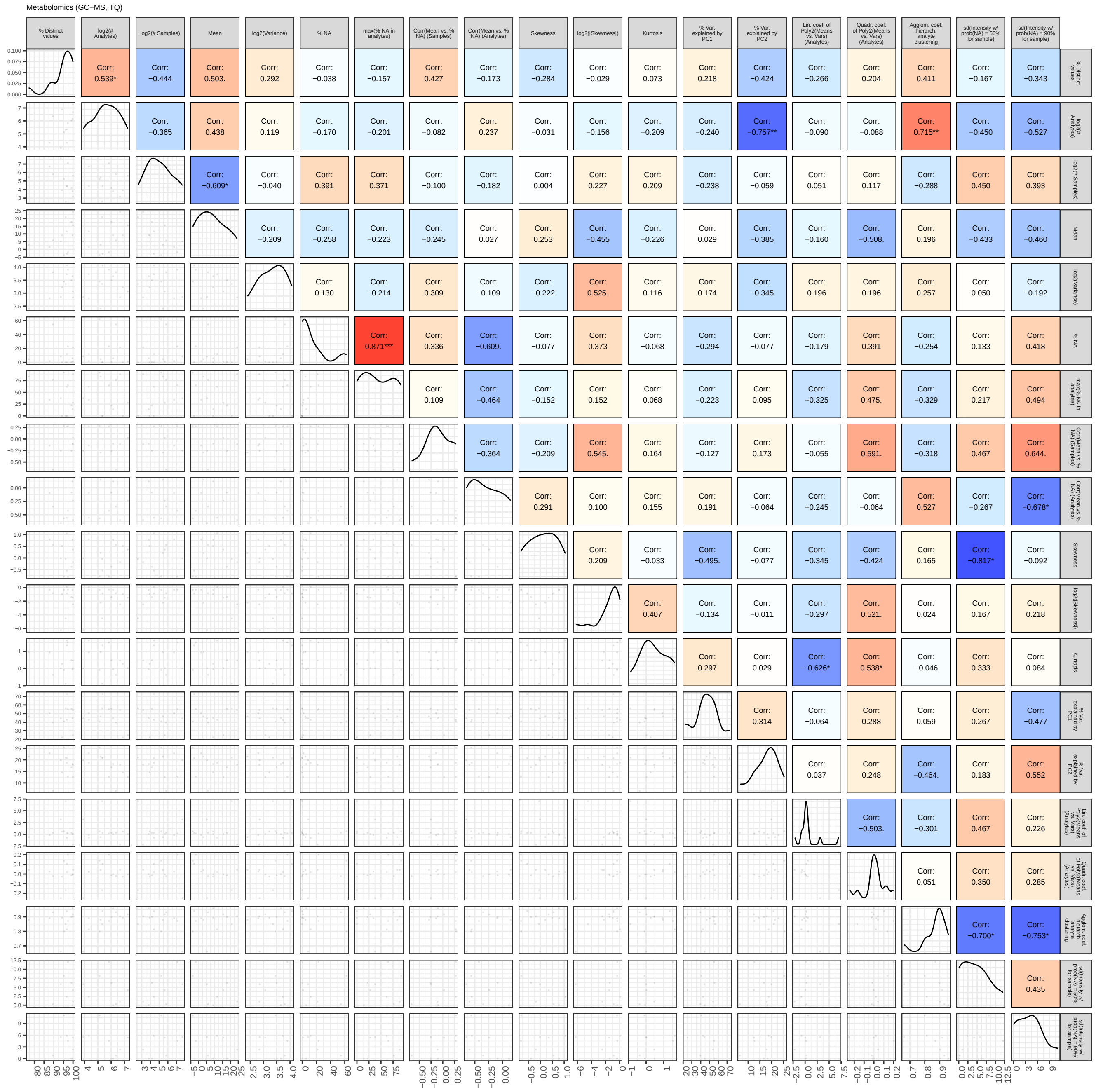

Figure S26. Spearman correlation plot of selected data characteristics across Metabolomics gas chromatography (GC)-triple quadrupole (TQ) mass spectrometry (MS) datasets. In the upper right corner the Spearman correlation coefficients for pairs of data characteristics are displayed, where *** = p<0.001, ** = p<0.01, * = p<0.05, and . = p<0.1. The intensity of red and blue corresponds to the strength of the positive and negative correlations, respectively. In the bottom left corner, scatter plots of pairs of data characteristics are shown, with each data point representing a dataset. On the diagonal, the distribution of each data characteristic is displayed.

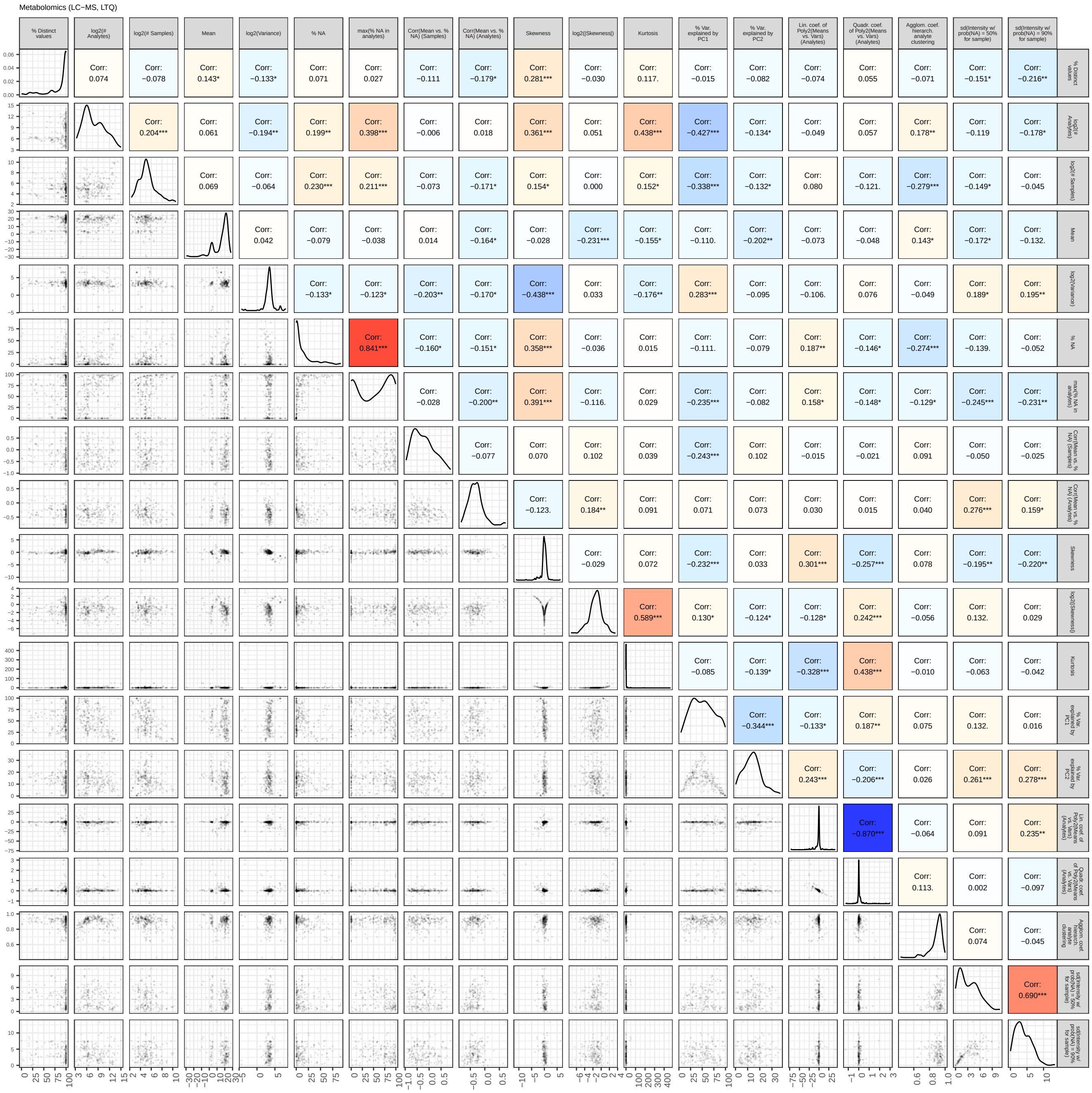

Figure S27. Spearman correlation plot of selected data characteristics across Metabolomics liquid chromatography (LC)-linear trap quadrupole (LTQ) mass spectrometry (MS) datasets. In the upper right corner the Spearman correlation coefficients for pairs of data characteristics are displayed, where *** = p<0.001, ** = p<0.01, * = p<0.05, and . = p<0.1. The intensity of red and blue corresponds to the strength of the positive and negative correlations, respectively. In the bottom left corner, scatter plots of pairs of data characteristics are shown, with each data point representing a dataset. On the diagonal, the distribution of each data characteristic is displayed.

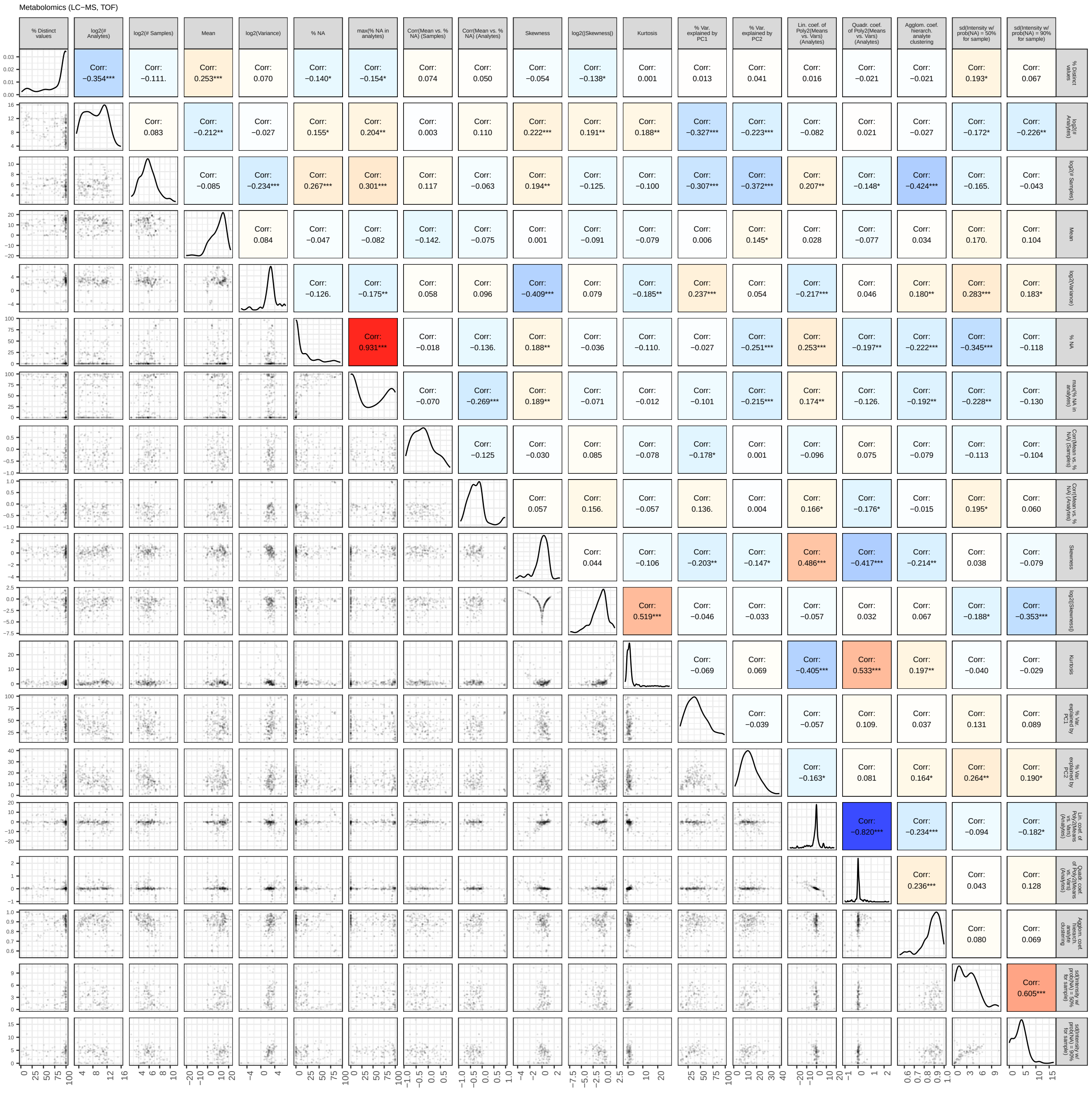

Figure S28. Spearman correlation plot of selected data characteristics across Metabolomics liquid chromatography (LC)-time-of-flight (TOF) mass spectrometry (MS) datasets. In the upper right corner the Spearman correlation coefficients for pairs of data characteristics are displayed, where *** = p<0.001, ** = p<0.01, * = p<0.05, and . = p<0.1. The intensity of red and blue corresponds to the strength of the positive and negative correlations, respectively. In the bottom left corner, scatter plots of pairs of data characteristics are shown, with each data point representing a dataset. On the diagonal, the distribution of each data characteristic is displayed.

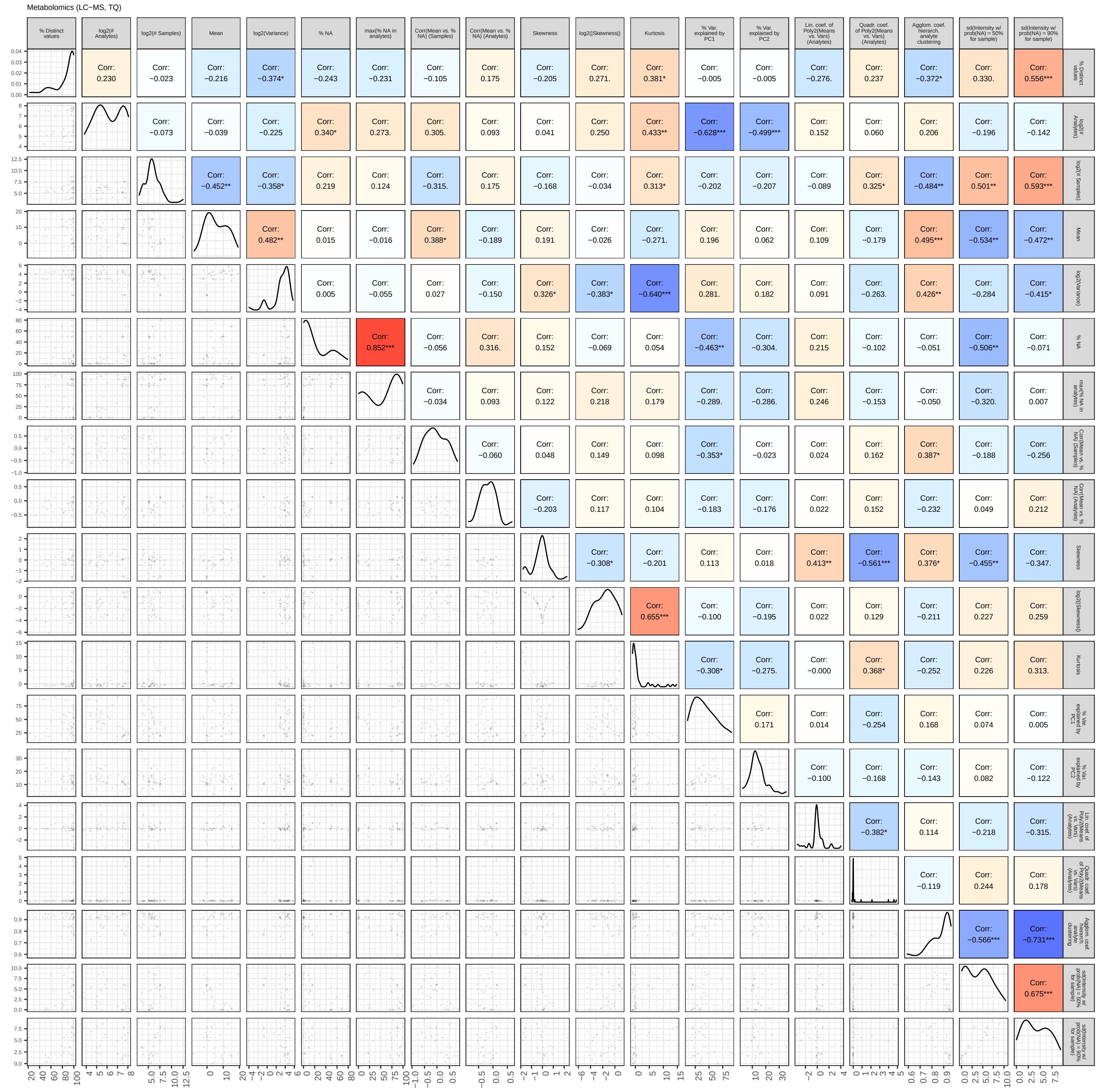

Figure S29. Spearman correlation plot of selected data characteristics across Metabolomics liquid chromatography (LC)-triple quadrupole (TQ) mass spectrometry (MS) datasets. In the upper right corner the Spearman correlation coefficients for pairs of data characteristics are displayed, where *** = $p<0.001$, ** = $p<0.01$, * = $p<0.05$, and . = $p<0.1$. The intensity of red and blue corresponds to the strength of the positive and negative correlations, respectively. In the bottom left corner, scatter plots of pairs of data characteristics are shown, with each data point representing a dataset. On the diagonal, the distribution of each data characteristic is displayed.

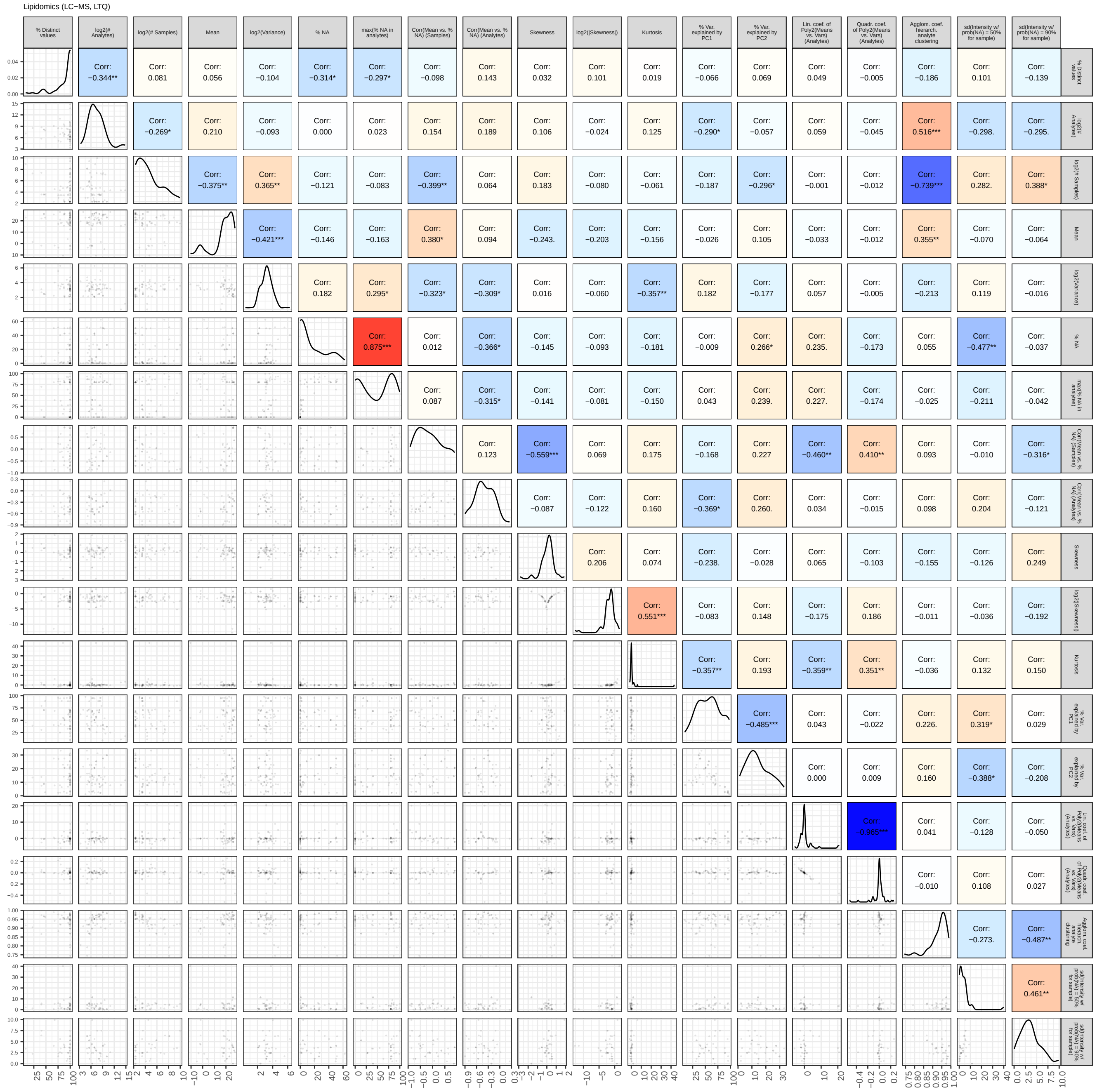

Figure S30. Spearman correlation plot of selected data characteristics across Lipidomics liquid chromatography (LC)-linear trap quadrupole (LTQ) mass spectrometry (MS) datasets. In the upper right corner the Spearman correlation coefficients for pairs of data characteristics are displayed, where *** = p<0.001, ** = p<0.01, * = p<0.05, and . = p<0.1. The intensity of red and blue corresponds to the strength of the positive and negative correlations, respectively. In the bottom left corner, scatter plots of pairs of data characteristics are shown, with each data point representing a dataset. On the diagonal, the distribution of each data characteristic is displayed.

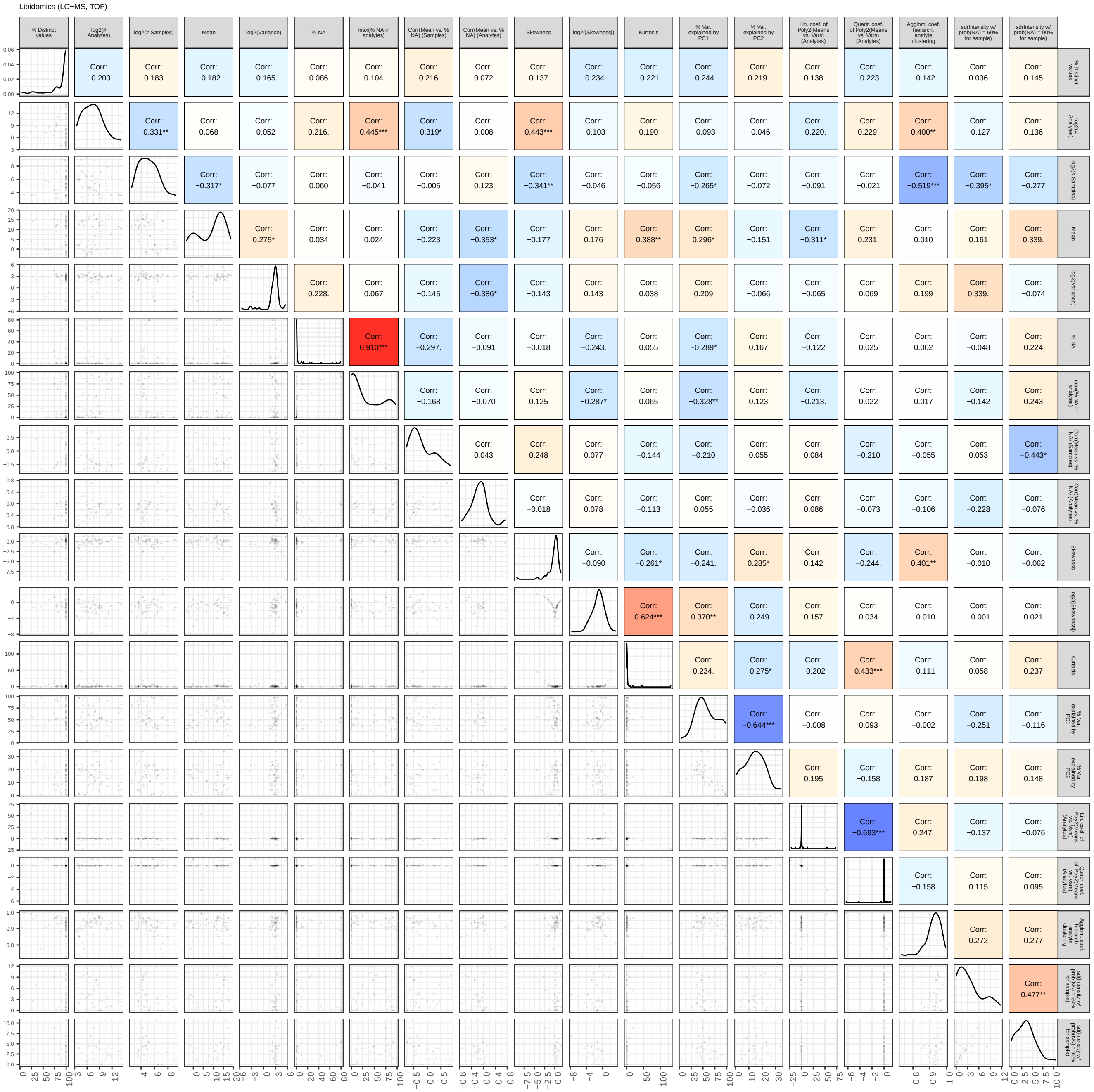

Figure S31. Spearman correlation plot of selected data characteristics across Lipidomics liquid chromatography (LC)-time-of-flight (TOF) mass spectrometry (MS) datasets. In the upper right corner the Spearman correlation coefficients for pairs of data characteristics are displayed, where *** = p<0.001, ** = p<0.01, * = p<0.05, and . = p<0.1. The intensity of red and blue corresponds to the strength of the positive and negative correlations, respectively. In the bottom left corner, scatter plots of pairs of data characteristics are shown, with each data point representing a dataset. On the diagonal, the distribution of each data characteristic is displayed.

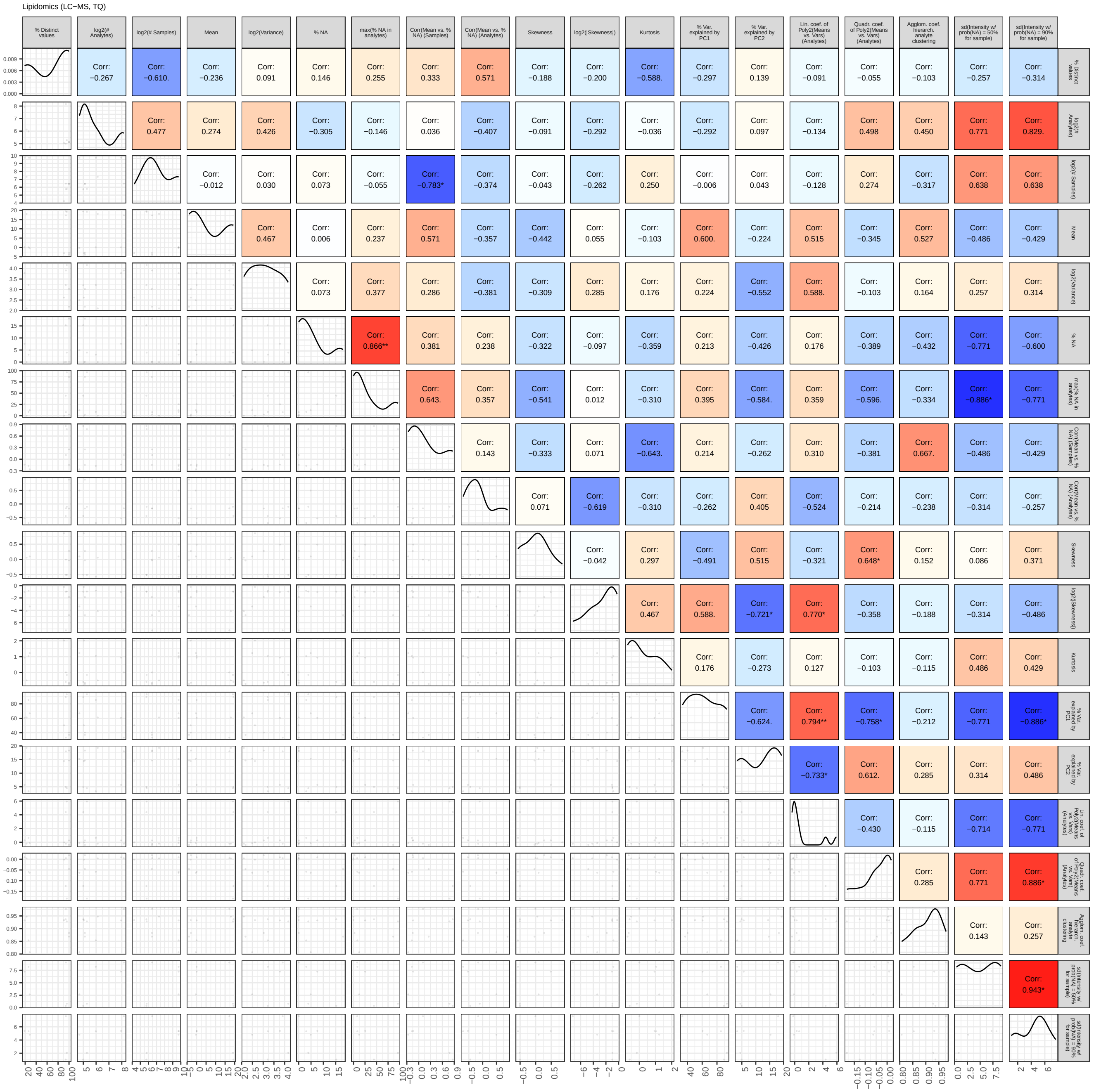

Figure S32. Spearman correlation plot of selected data characteristics across Lipidomics liquid chromatography (LC)-triple quadrupole (TQ) mass spectrometry (MS) datasets. In the upper right corner the Spearman correlation coefficients for pairs of data characteristics are displayed, where *** = p<0.001, ** = p<0.01, * = p<0.05, and . = p<0.1. The intensity of red and blue corresponds to the strength of the positive and negative correlations, respectively. In the bottom left corner, scatter plots of pairs of data characteristics are shown, with each data point representing a dataset. On the diagonal, the distribution of each data characteristic is displayed.

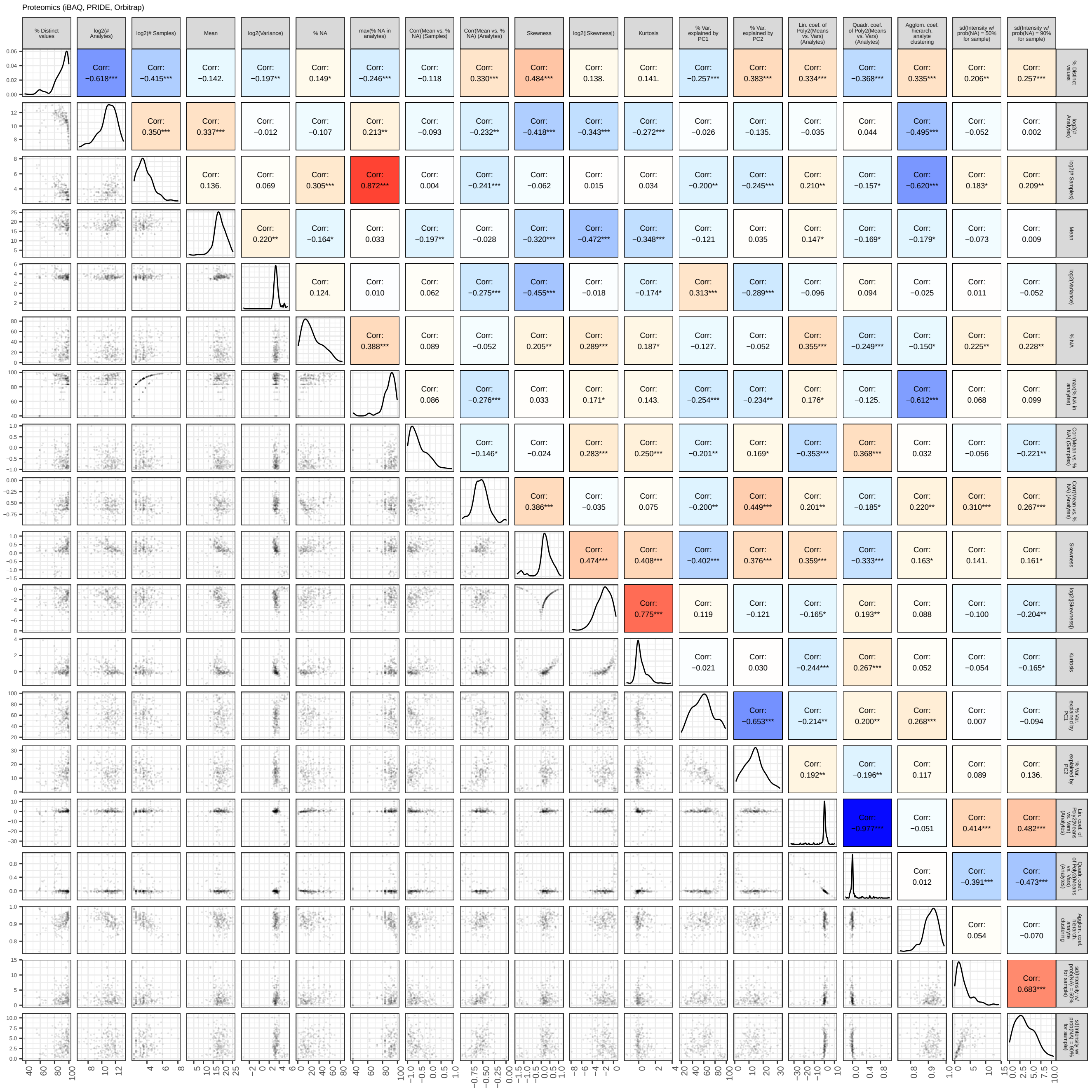

Figure S33. Spearman correlation plot of selected data characteristics across Proteomics (iBAQ, PRIDE, Orbitrap) datasets. In the upper right corner the Spearman correlation coefficients for pairs of data characteristics are displayed, where *** = p<0.001, ** = p<0.01, * = p<0.05, and . = p<0.1. The intensity of red and blue corresponds to the strength of the positive and negative correlations, respectively. In the bottom left corner, scatter plots of pairs of data characteristics are shown, with each data point representing a dataset. On the diagonal, the distribution of each data characteristic is displayed.

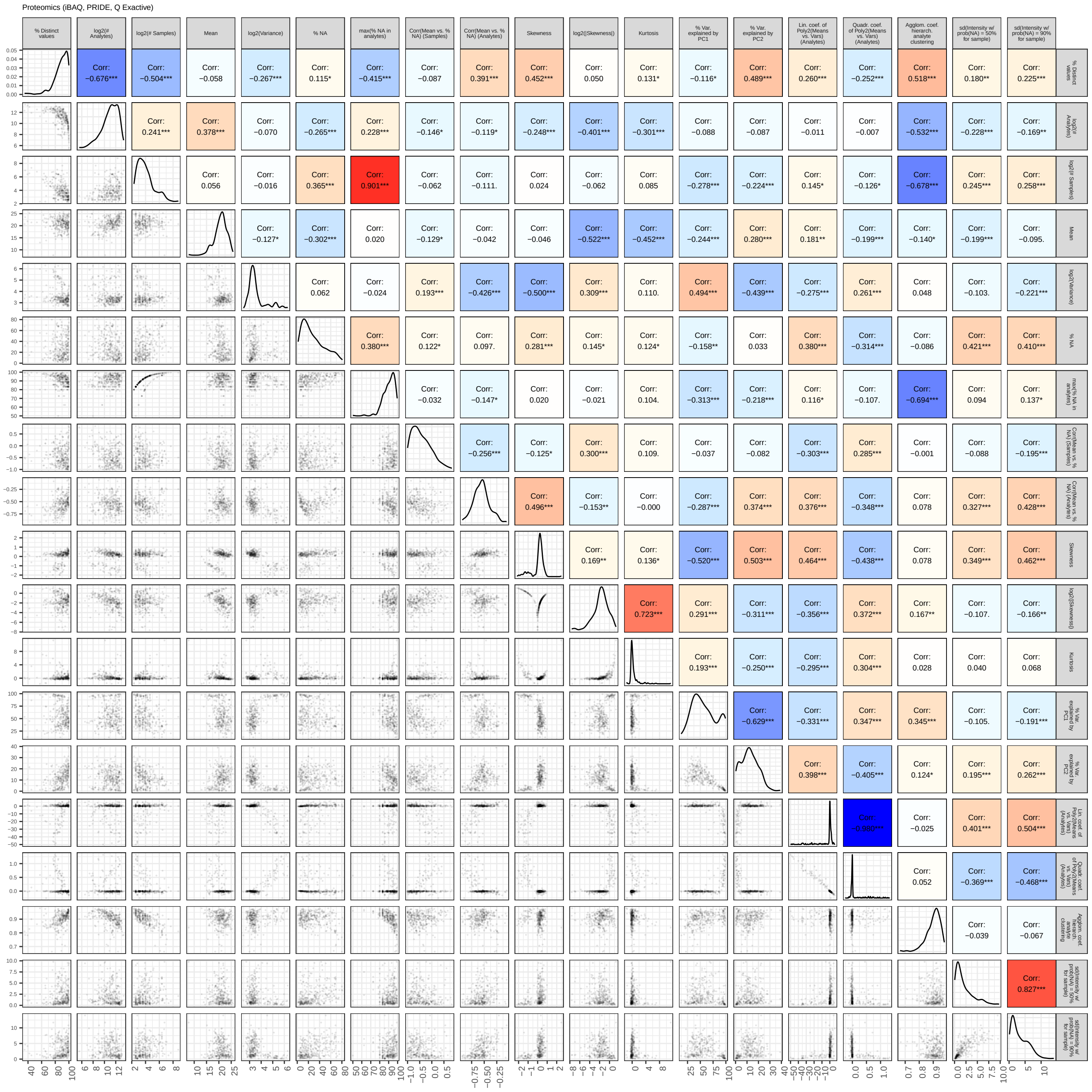

Figure S34. Spearman correlation plot of selected data characteristics across Proteomics (iBAQ, PRIDE, Q Exactive) datasets. In the upper right corner the Spearman correlation coefficients for pairs of data characteristics are displayed, where *** = p<0.001, ** = p<0.01, * = p<0.05, and . = p<0.1. The intensity of red and blue corresponds to the strength of the positive and negative correlations, respectively. In the bottom left corner, scatter plots of pairs of data characteristics are shown, with each data point representing a dataset. On the diagonal, the distribution of each data characteristic is displayed.

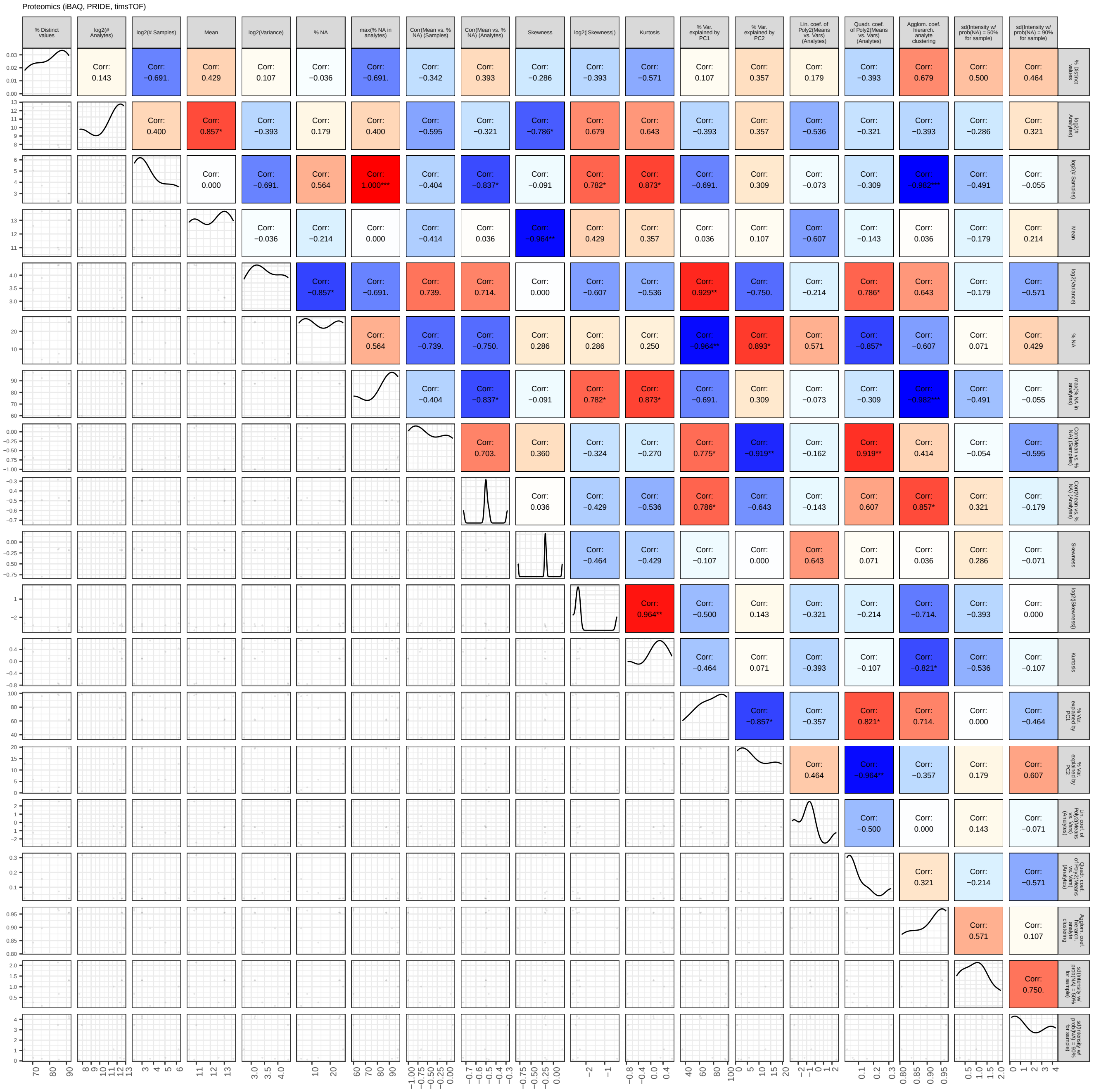

Figure S35. Spearman correlation plot of selected data characteristics across Proteomics (iBAQ, PRIDE, timsTOF) datasets. In the upper right corner the Spearman correlation coefficients for pairs of data characteristics are displayed, where *** = p<0.001, ** = p<0.01, * = p<0.05, and . = p<0.1.The intensity of red and blue corresponds to the strength of the positive and negative correlations, respectively. In the bottom left corner, scatter plots of pairs of data characteristics are shown, with each data point representing a dataset. On the diagonal, the distribution of each data characteristic is displayed.

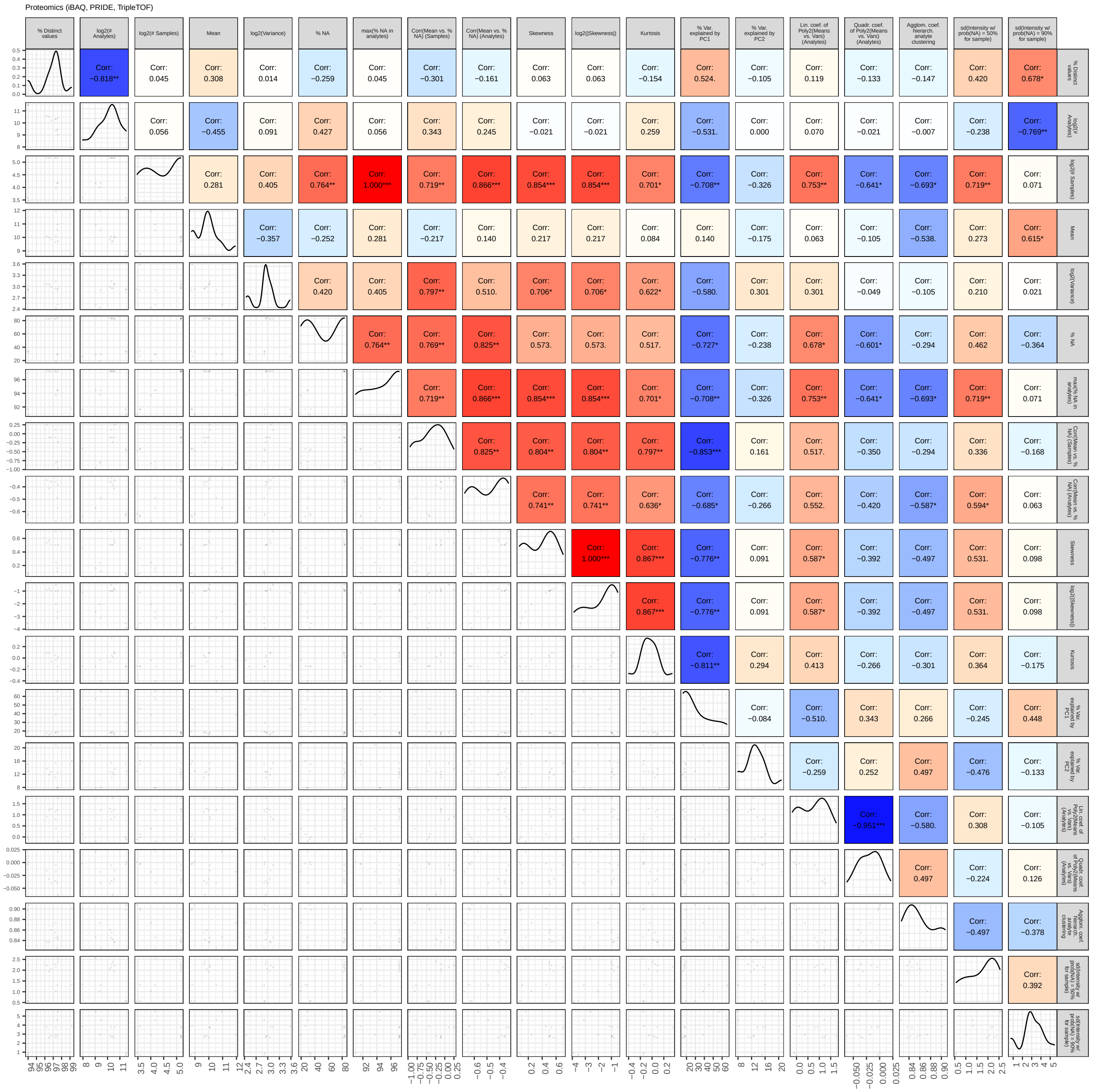

Figure S36. Spearman correlation plot of selected data characteristics across Proteomics (iBAQ, PRIDE, TripleTOF) datasets. In the upper right corner the Spearman correlation coefficients for pairs of data characteristics are displayed, where *** = p<0.001, ** = p<0.01, * = p<0.05, and . = p<0.1. The intensity of red and blue corresponds to the strength of the positive and negative correlations, respectively. In the bottom left corner, scatter plots of pairs of data characteristics are shown, with each data point representing a dataset. On the diagonal, the distribution of each data characteristic is displayed.

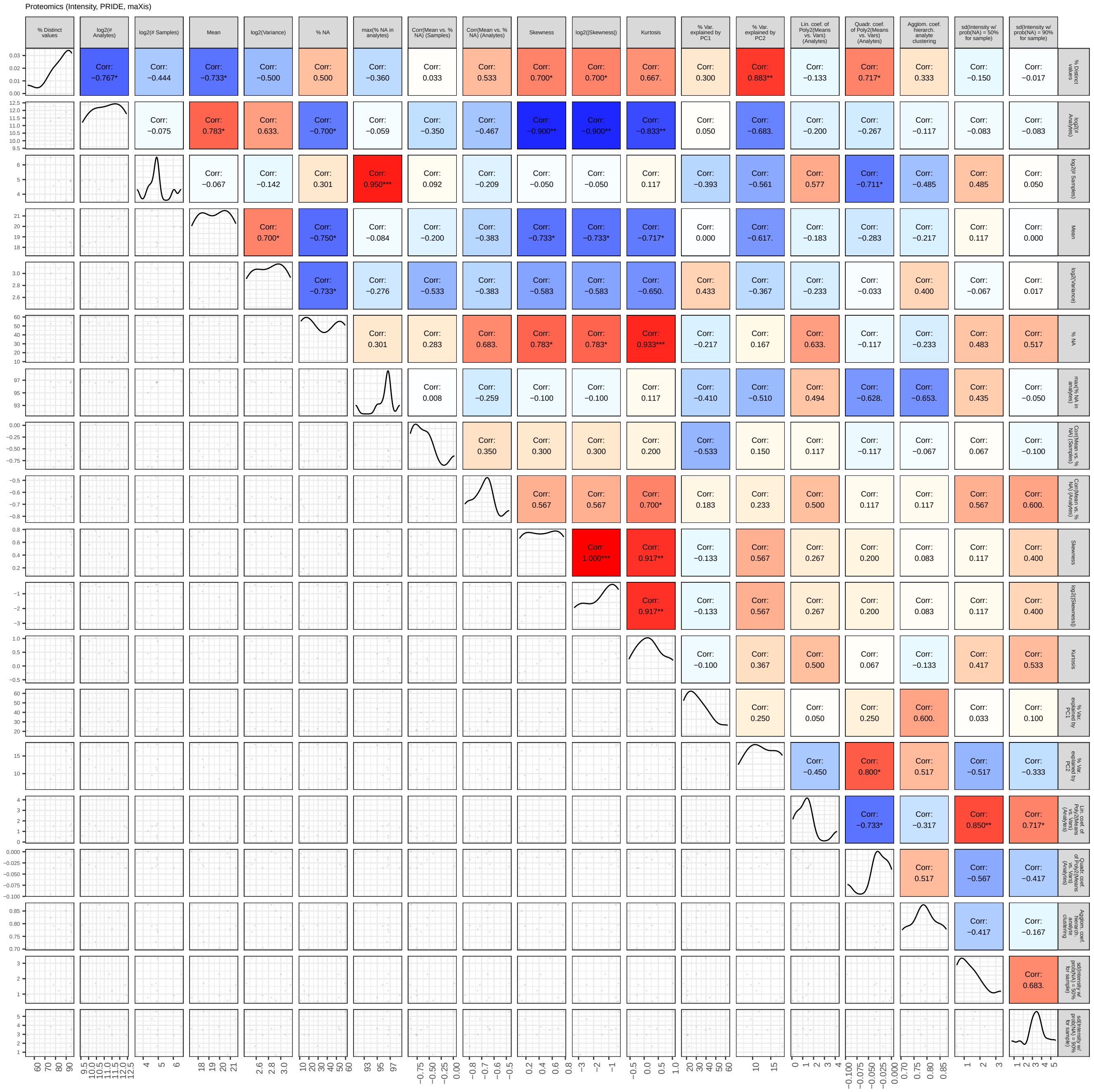

Figure S37. Spearman correlation plot of selected data characteristics across Proteomics (Intensity, PRIDE, maXis) datasets. In the upper right corner the Spearman correlation coefficients for pairs of data characteristics are displayed, where *** = p<0.001, ** = p<0.01, * = p<0.05, and . = p<0.1. The intensity of red and blue corresponds to the strength of the positive and negative correlations, respectively. In the bottom left corner, scatter plots of pairs of data characteristics are shown, with each data point representing a dataset. On the diagonal, the distribution of each data characteristic is displayed.

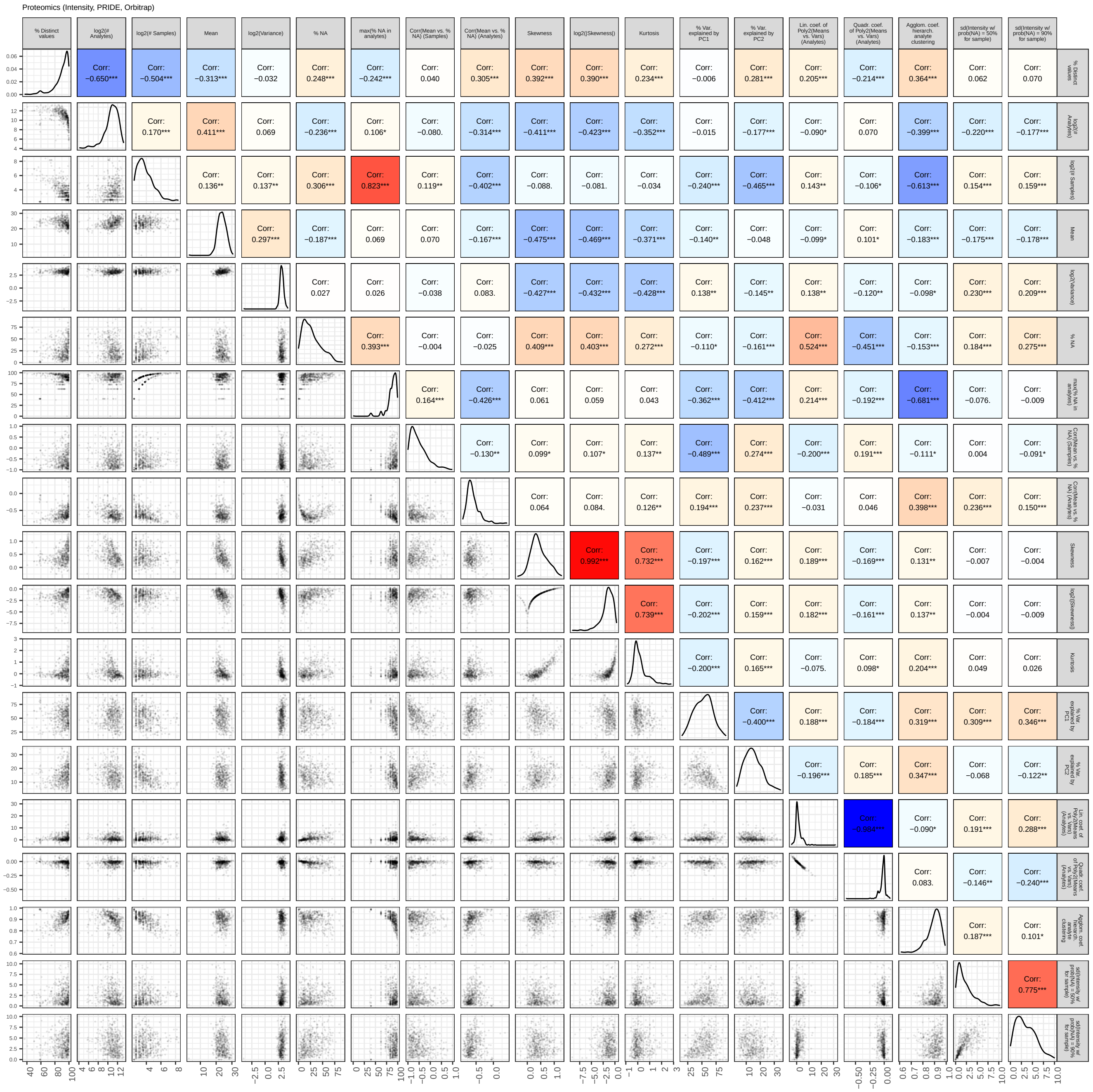

Figure S38. Spearman correlation plot of selected data characteristics across Proteomics (Intensity, PRIDE, Orbitrap) datasets. In the upper right corner the Spearman correlation coefficients for pairs of data characteristics are displayed, where *** = p<0.001, ** = p<0.01, * = p<0.05, and . = p<0.1. The intensity of red and blue corresponds to the strength of the positive and negative correlations, respectively. In the bottom left corner, scatter plots of pairs of data characteristics are shown, with each data point representing a dataset. On the diagonal, the distribution of each data characteristic is displayed.

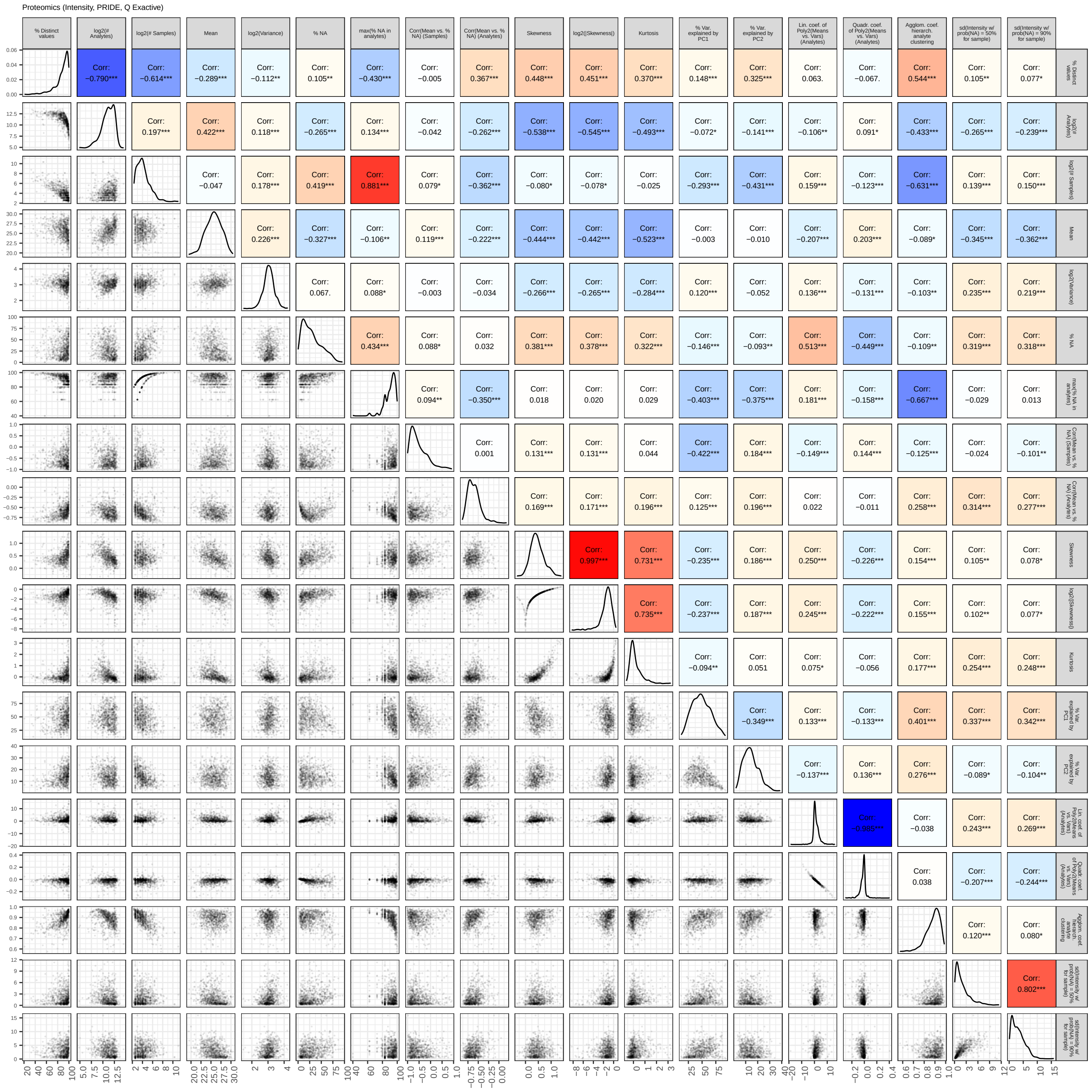

Figure S39. Spearman correlation plot of selected data characteristics across Proteomics (Intensity, PRIDE, Q Exactive) datasets. In the upper right corner the Spearman correlation coefficients for pairs of data characteristics are displayed, where *** = p<0.001, ** = p<0.01, * = p<0.05, and . = p<0.1. The intensity of red and blue corresponds to the strength of the positive and negative correlations, respectively. In the bottom left corner, scatter plots of pairs of data characteristics are shown, with each data point representing a dataset. On the diagonal, the distribution of each data characteristic is displayed.

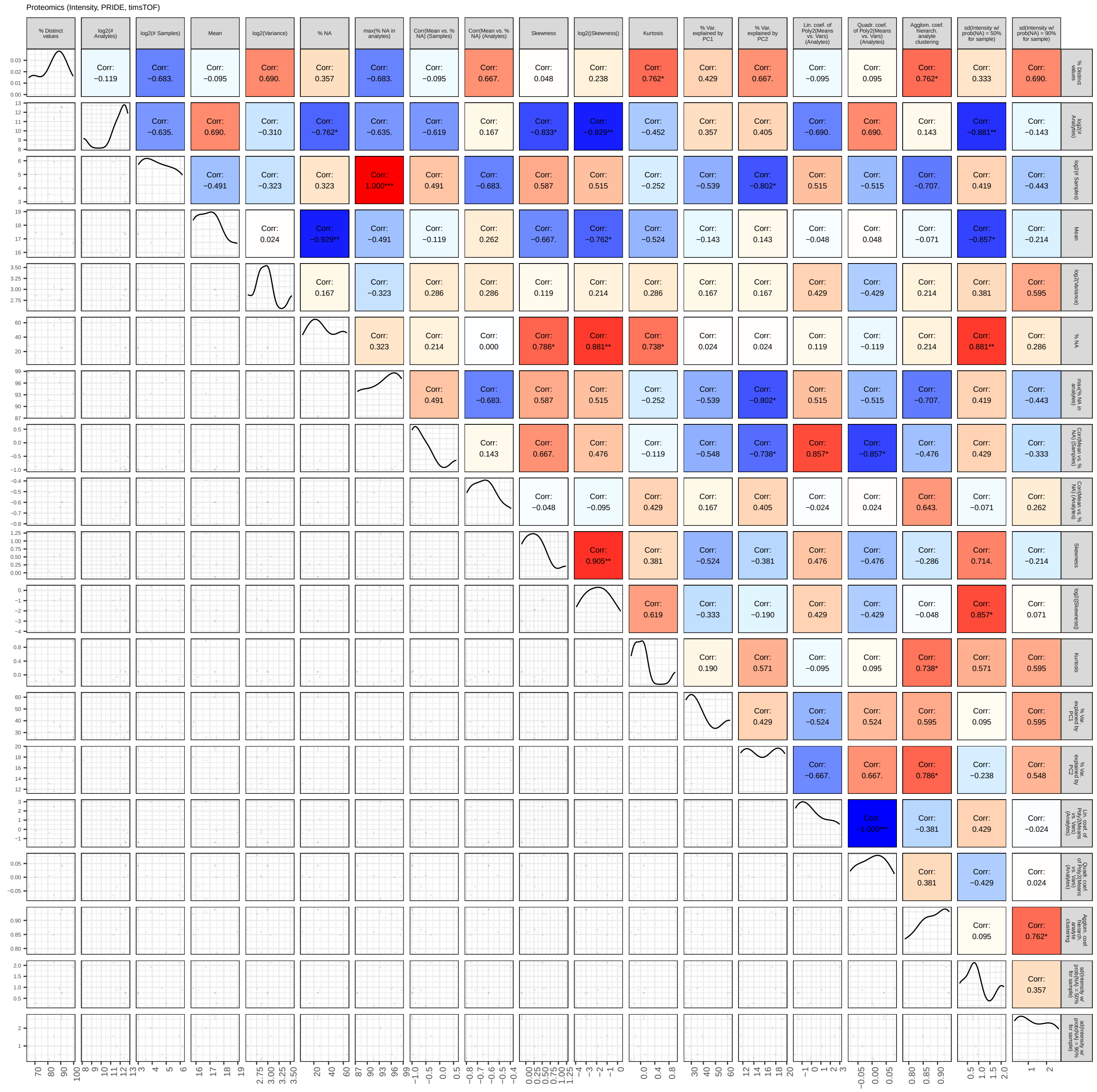

Figure S40. Spearman correlation plot of selected data characteristics across Proteomics (Intensity, PRIDE, timsTOF) datasets. In the upper right corner the Spearman correlation coefficients for pairs of data characteristics are displayed, where *** = p<0.001, ** = p<0.01, * = p<0.05, and . = p<0.1. The intensity of red and blue corresponds to the strength of the positive and negative correlations, respectively. In the bottom left corner, scatter plots of pairs of data characteristics are shown, with each data point representing a dataset. On the diagonal, the distribution of each data characteristic is displayed.

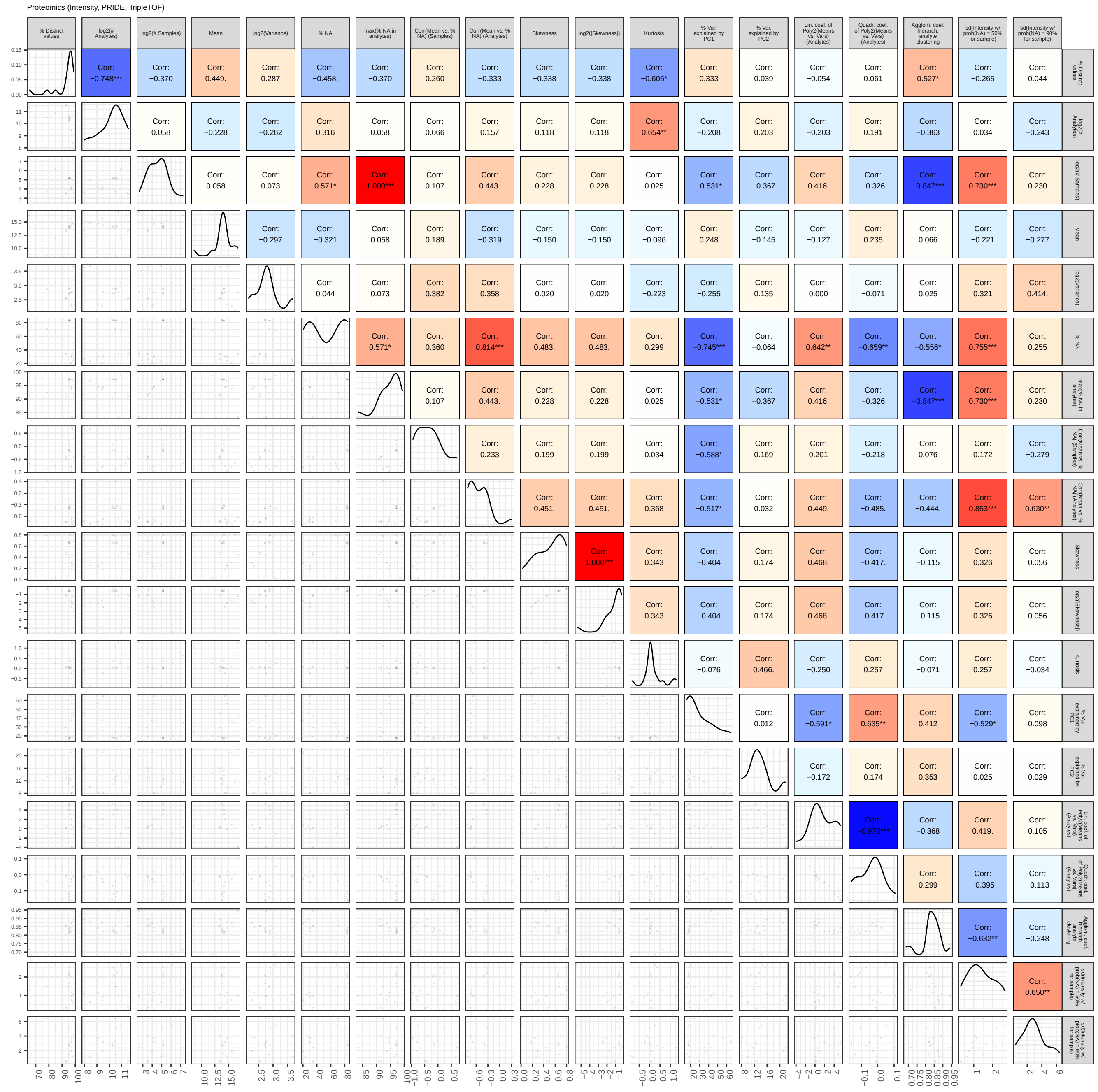

Figure S41. Spearman correlation plot of selected data characteristics across Proteomics (Intensity, PRIDE, TripleTOF) datasets. In the upper right corner the Spearman correlation coefficients for pairs of data characteristics are displayed, where *** = p<0.001, ** = p<0.01, * = p<0.05, and . = p<0.1. The intensity of red and blue corresponds to the strength of the positive and negative correlations, respectively. In the bottom left corner, scatter plots of pairs of data characteristics are shown, with each data point representing a dataset. On the diagonal, the distribution of each data characteristic is displayed.

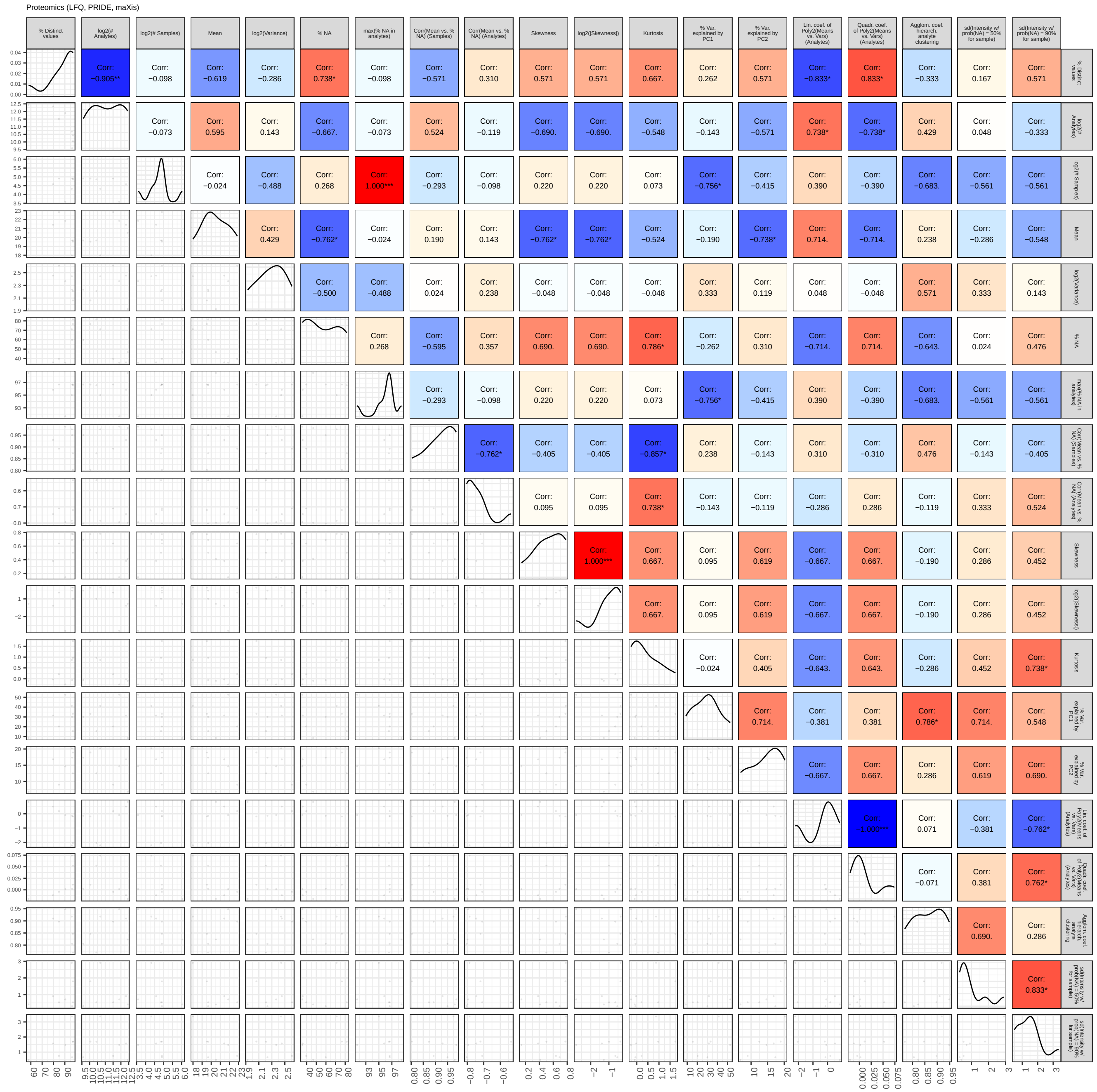

Figure S42. Spearman correlation plot of selected data characteristics across Proteomics (LFQ, PRIDE, maXis) datasets. In the upper right corner the Spearman correlation coefficients for pairs of data characteristics are displayed, where *** = p<0.001, ** = p<0.01, * = p<0.05, and . = p<0.1. The intensity of red and blue corresponds to the strength of the positive and negative correlations, respectively. In the bottom left corner, scatter plots of pairs of data characteristics are shown, with each data point representing a dataset. On the diagonal, the distribution of each data characteristic is displayed.

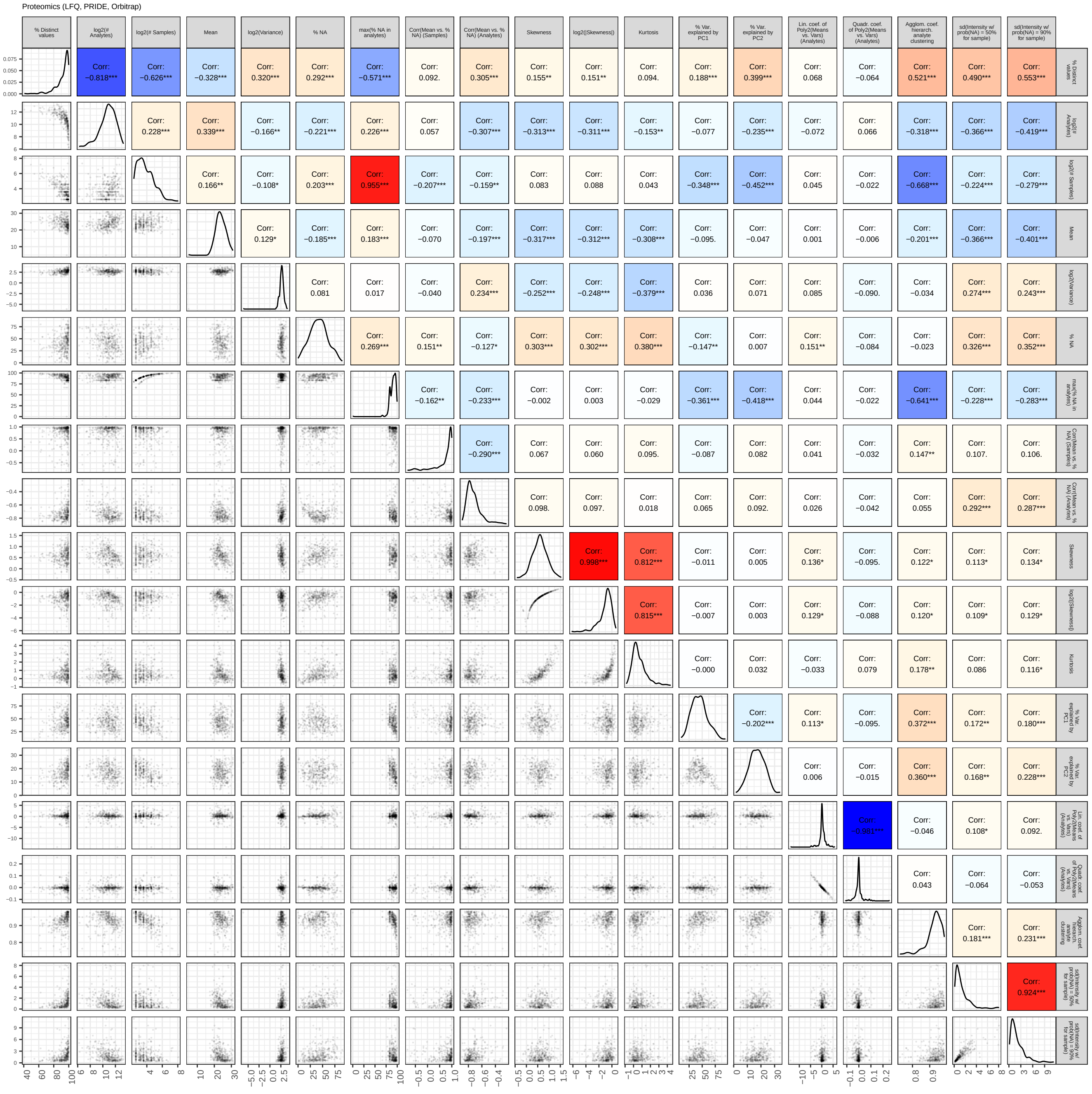

Figure S43. Spearman correlation plot of selected data characteristics across Proteomics (LFQ, PRIDE, Orbitrap) datasets. In the upper right corner the Spearman correlation coefficients for pairs of data characteristics are displayed, where *** = p<0.001, ** = p<0.01, * = p<0.05, and . = p<0.1. The intensity of red and blue corresponds to the strength of the positive and negative correlations, respectively. In the bottom left corner, scatter plots of pairs of data characteristics are shown, with each data point representing a dataset. On the diagonal, the distribution of each data characteristic is displayed.

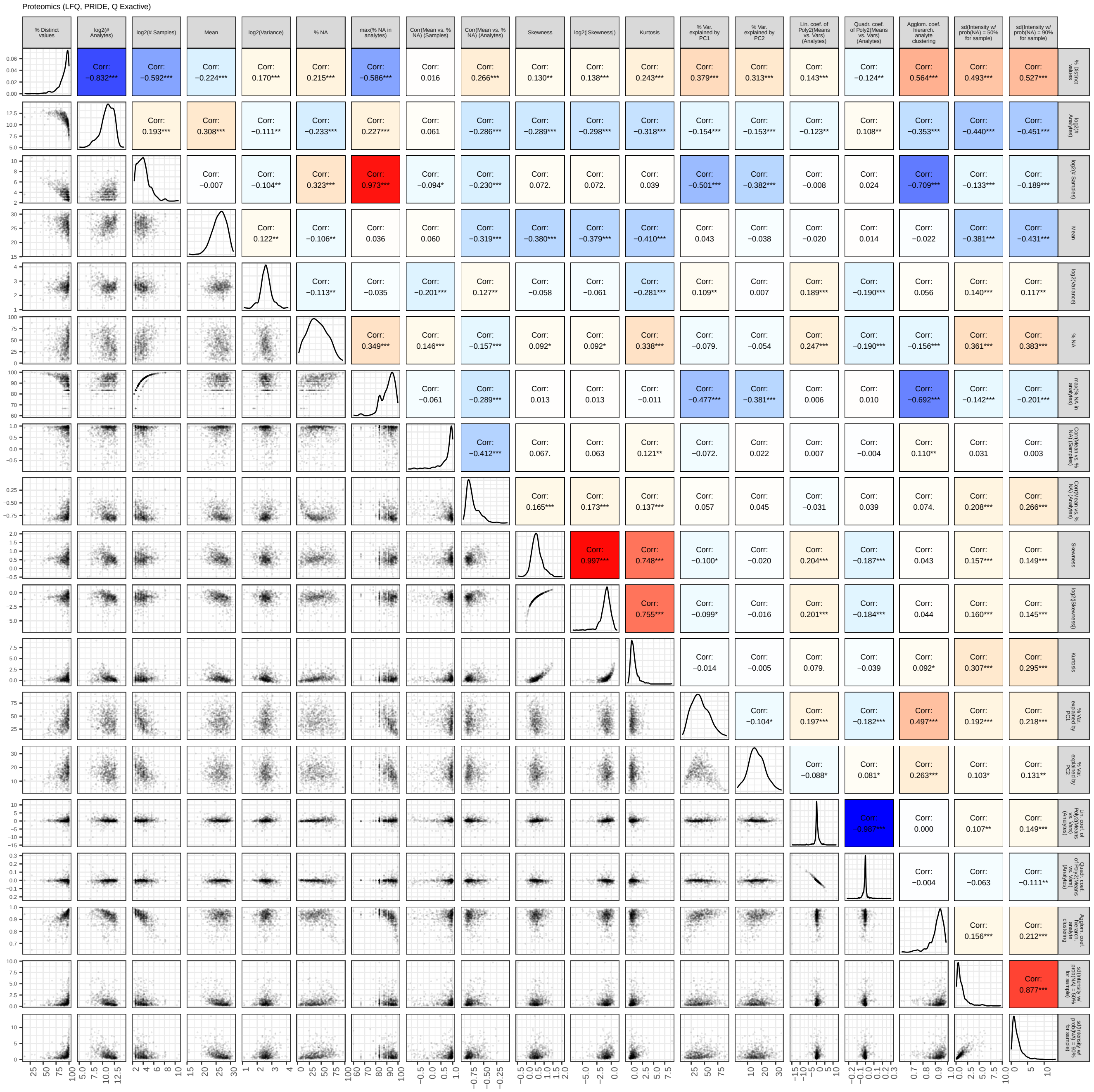

Figure S44. Spearman correlation plot of selected data characteristics across Proteomics (LFQ, PRIDE, Q Exactive) datasets. In the upper right corner the Spearman correlation coefficients for pairs of data characteristics are displayed, where *** = p<0.001, ** = p<0.01, * = p<0.05, and . = p<0.1. The intensity of red and blue corresponds to the strength of the positive and negative correlations, respectively. In the bottom left corner, scatter plots of pairs of data characteristics are shown, with each data point representing a dataset. On the diagonal, the distribution of each data characteristic is displayed.

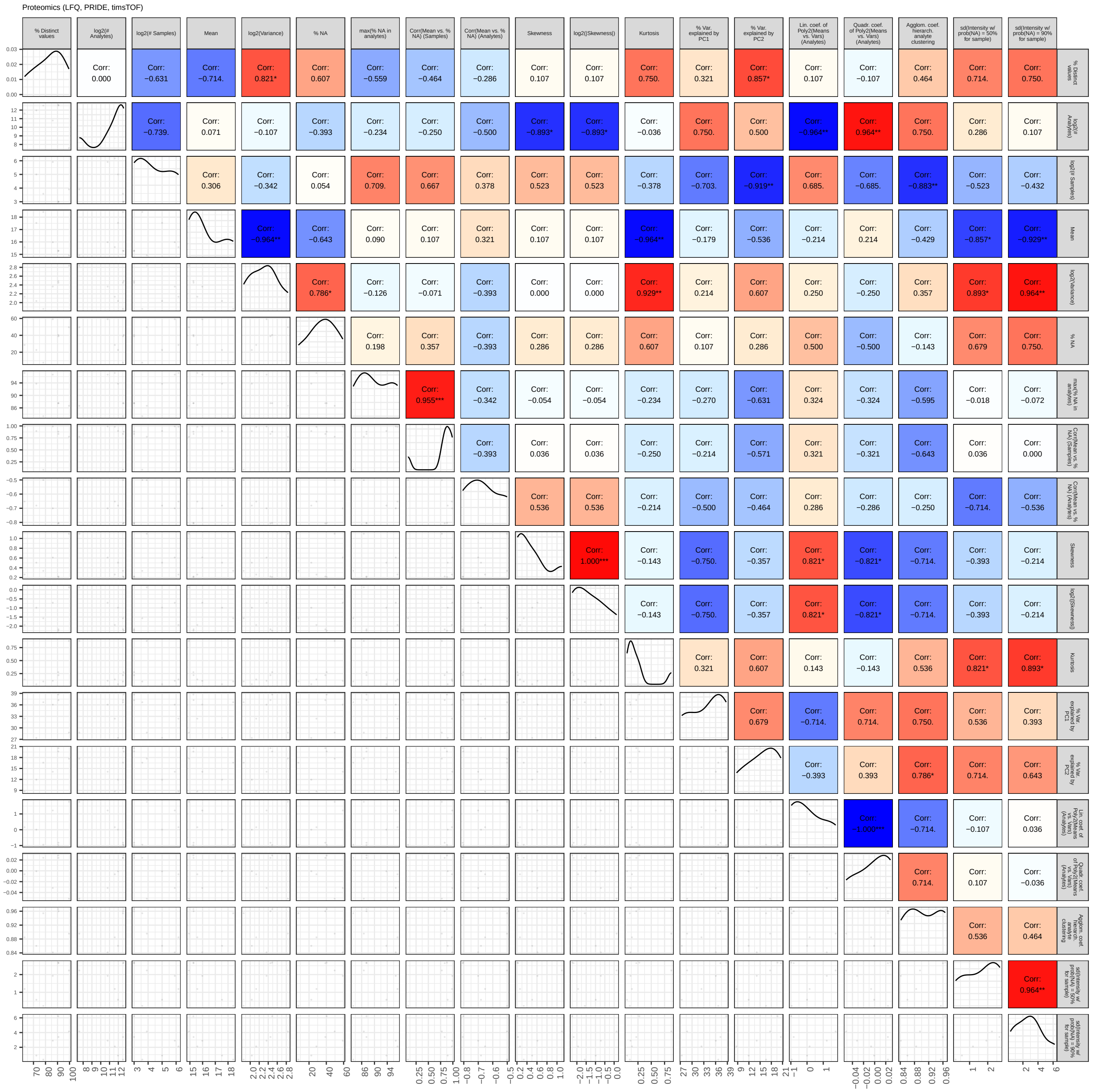

Figure S45. Spearman correlation plot of selected data characteristics across Proteomics (LFQ, PRIDE, timsTOF) datasets. In the upper right corner the Spearman correlation coefficients for pairs of data characteristics are displayed, where *** = p<0.001, ** = p<0.01, * = p<0.05, and . = p<0.1. The intensity of red and blue corresponds to the strength of the positive and negative correlations, respectively. In the bottom left corner, scatter plots of pairs of data characteristics are shown, with each data point representing a dataset. On the diagonal, the distribution of each data characteristic is displayed.

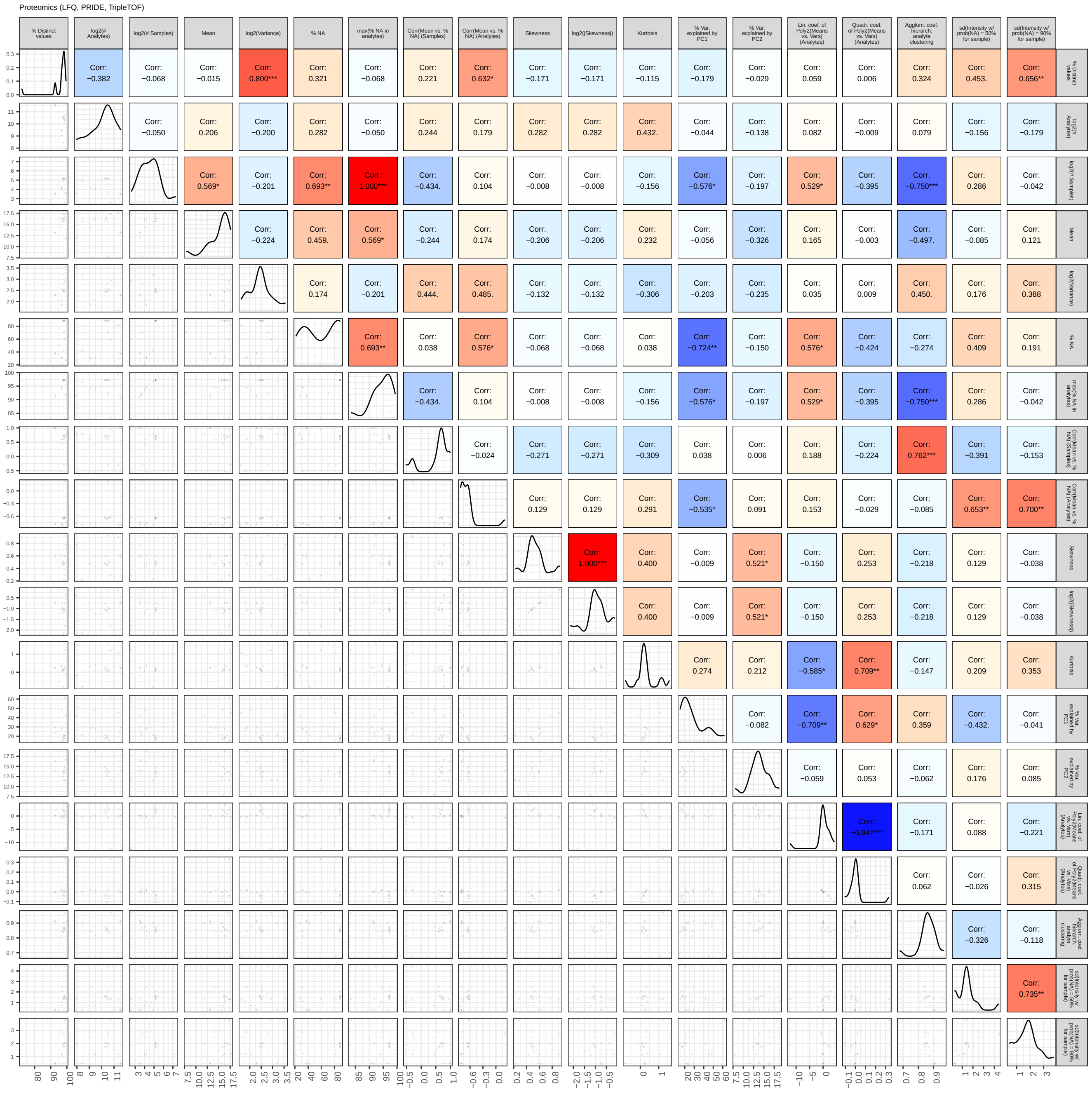

Figure S46. Spearman correlation plot of selected data characteristics across Proteomics (LFQ, PRIDE, TripleTOF) datasets. In the upper right corner the Spearman correlation coefficients for pairs of data characteristics are displayed, where *** = p<0.001, ** = p<0.01, * = p<0.05, and . = p<0.1. The intensity of red and blue corresponds to the strength of the positive and negative correlations, respectively. In the bottom left corner, scatter plots of pairs of data characteristics are shown, with each data point representing a dataset. On the diagonal, the distribution of each data characteristic is displayed.

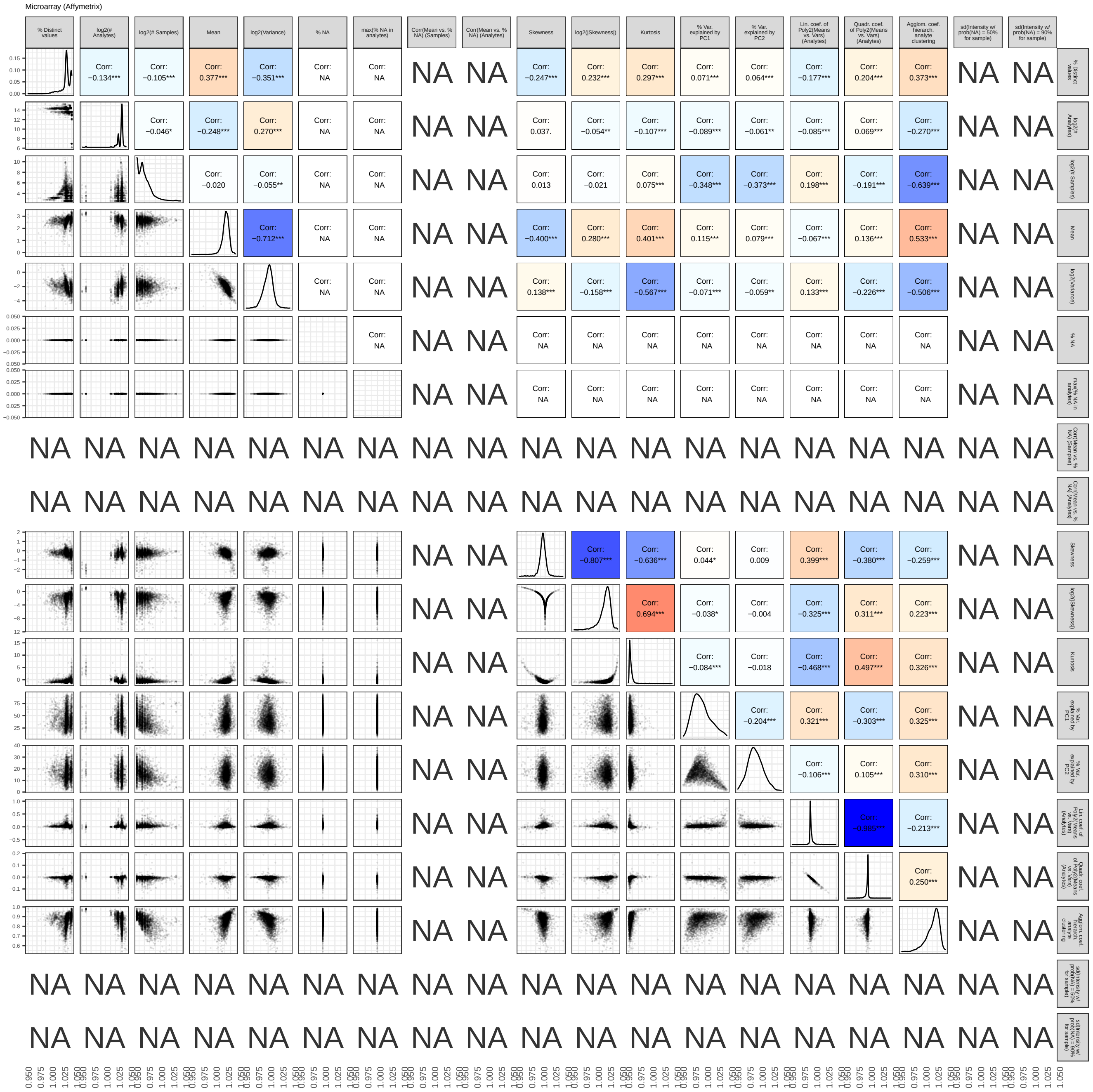

Figure S47. Spearman correlation plot of selected data characteristics across Microarray (Affymetrix) datasets. In the upper right corner the Spearman correlation coefficients for pairs of data characteristics are displayed, where *** = p<0.001, ** = p<0.01, * = p<0.05, and . = p<0.1. The intensity of red and blue corresponds to the strength of the positive and negative correlations, respectively. In the bottom left corner, scatter plots of pairs of data characteristics are shown, with each data point representing a dataset. On the diagonal, the distribution of each data characteristic is displayed. As microarray data does not contain missing values, no correlations could be calculated for data characteristics related to missing values, indicated by ‘NA’.

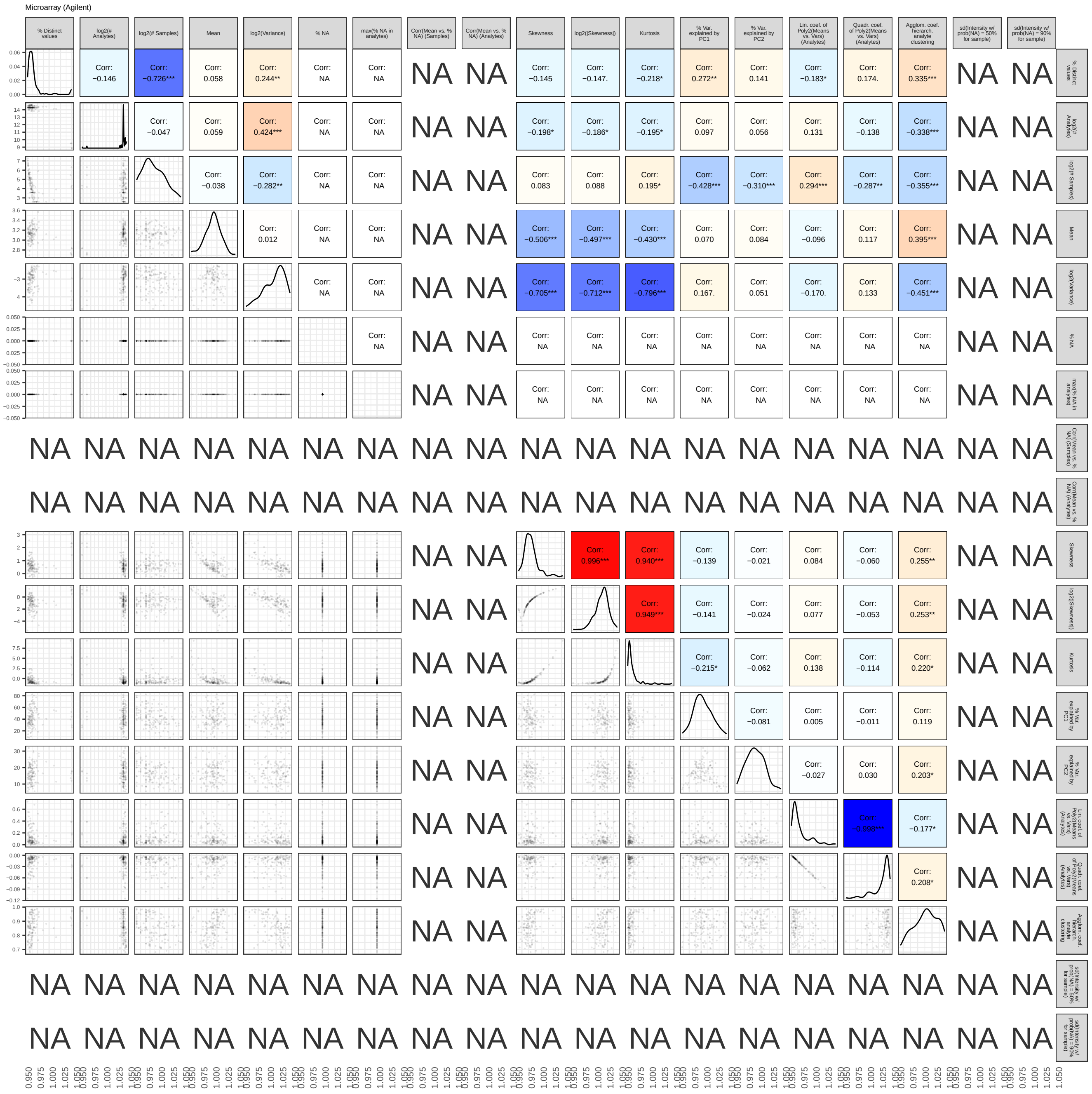

Figure S48. Spearman correlation plot of selected data characteristics across Microarray (Agilent) datasets. In the upper right corner the Spearman correlation coefficients for pairs of data characteristics are displayed, where *** = p<0.001, ** = p<0.01, * = p<0.05, and . = p<0.1. The intensity of red and blue corresponds to the strength of the positive and negative correlations, respectively. In the bottom left corner, scatter plots of pairs of data characteristics are shown, with each data point representing a dataset. On the diagonal, the distribution of each data characteristic is displayed. As microarray data does not contain missing values, no correlations could be calculated for data characteristics related to missing values, indicated by 'NA'.

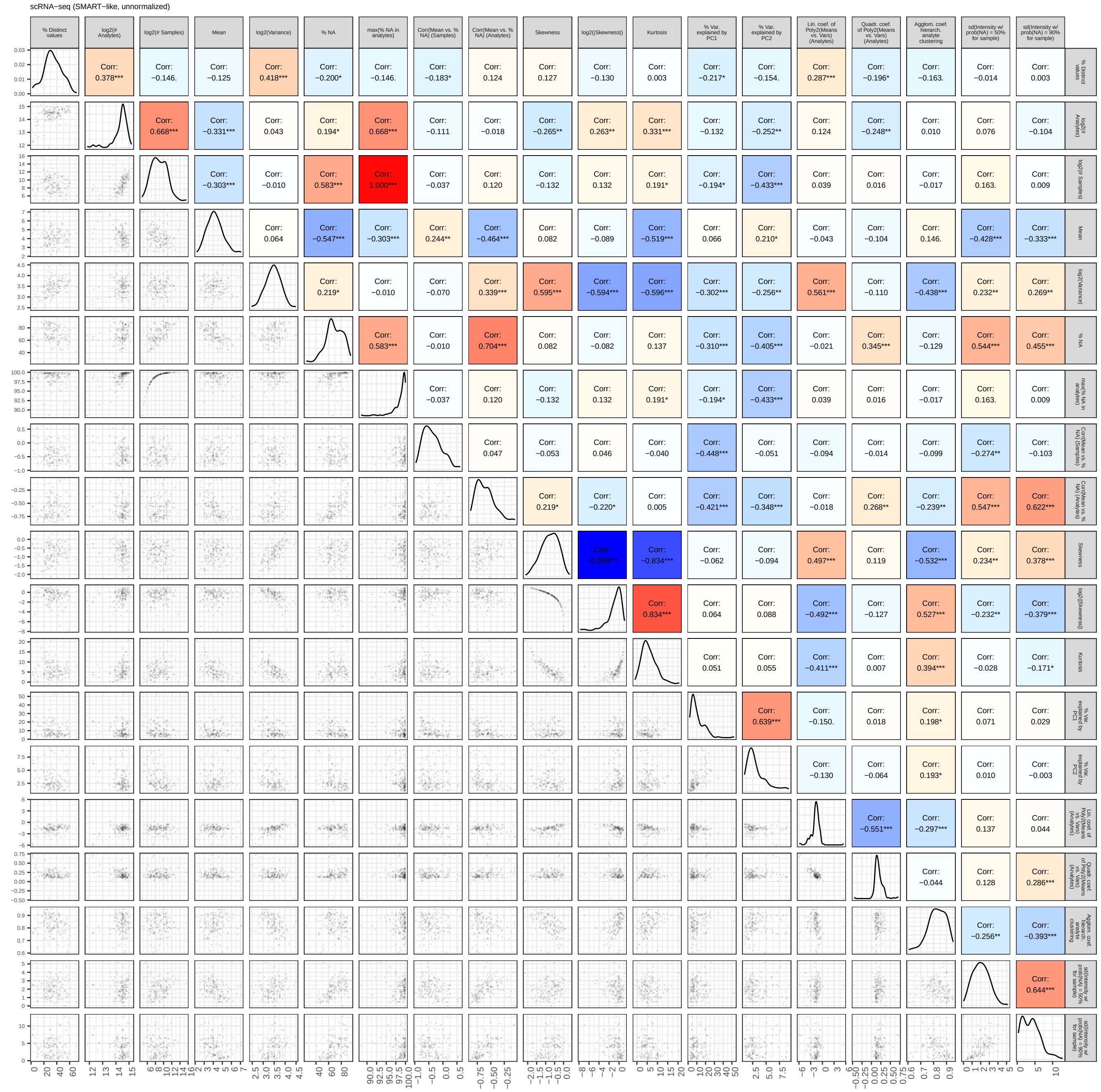

Figure S49. Spearman correlation plot of selected data characteristics across scRNA-seq (SMART-like, unnormalized) datasets. In the upper right corner the Spearman correlation coefficients for pairs of data characteristics are displayed, where *** = p<0.001, ** = p<0.01, * = p<0.05, and . = p<0.1. The intensity of red and blue corresponds to the strength of the positive and negative correlations, respectively. In the bottom left corner, scatter plots of pairs of data characteristics are shown, with each data point representing a dataset. On the diagonal, the distribution of each data characteristic is displayed.

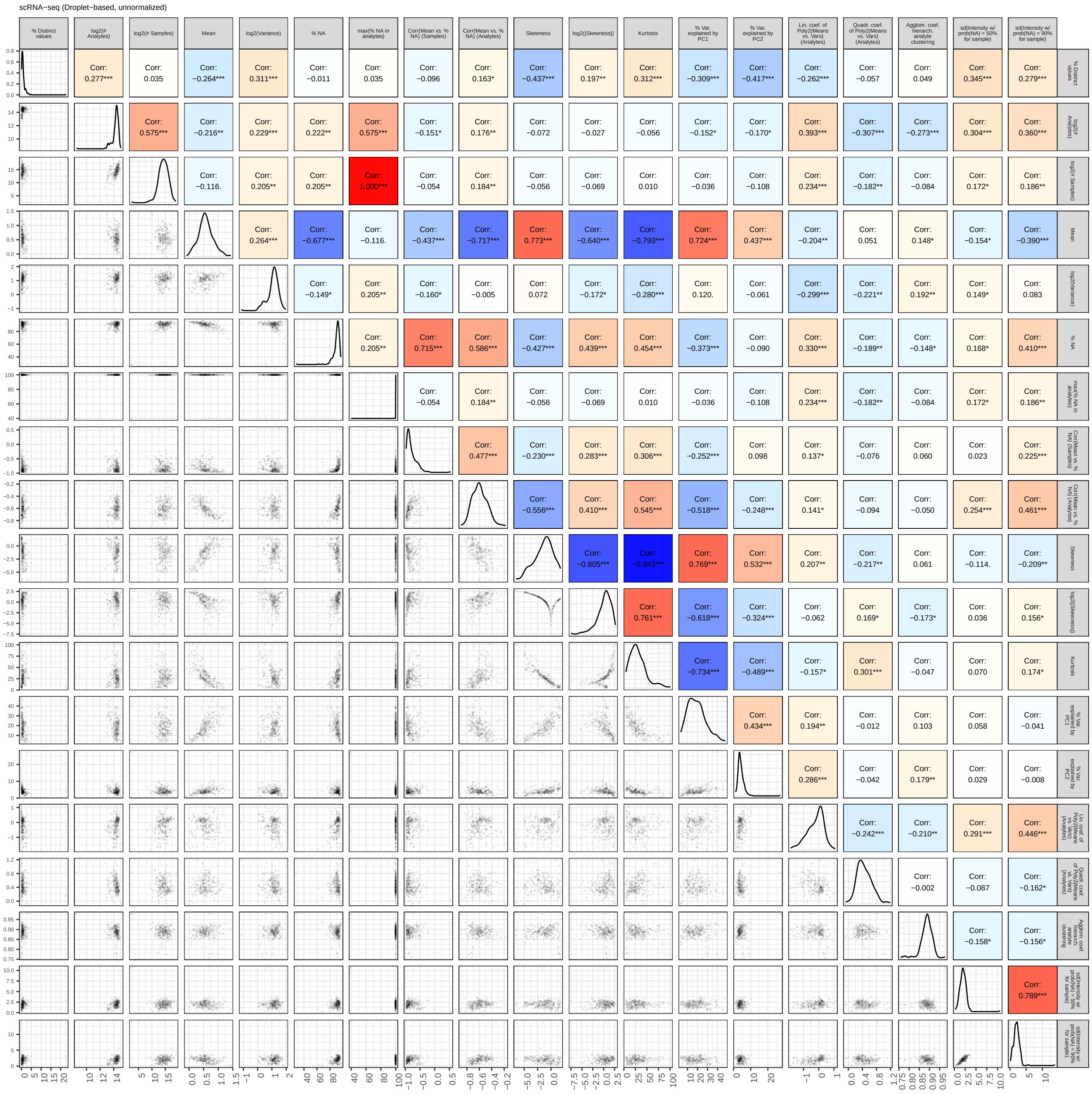

Figure S50. Spearman correlation plot of selected data characteristics across scRNA-seq (Droplet-based, unnormalized) datasets. In the upper right corner the Spearman correlation coefficients for pairs of data characteristics are displayed, where *** = p<0.001, ** = p<0.01, * = p<0.05, and . = p<0.1. The intensity of red and blue corresponds to the strength of the positive and negative correlations, respectively. In the bottom left corner, scatter plots of pairs of data characteristics are shown, with each data point representing a dataset. On the diagonal, the distribution of each data characteristic is displayed.

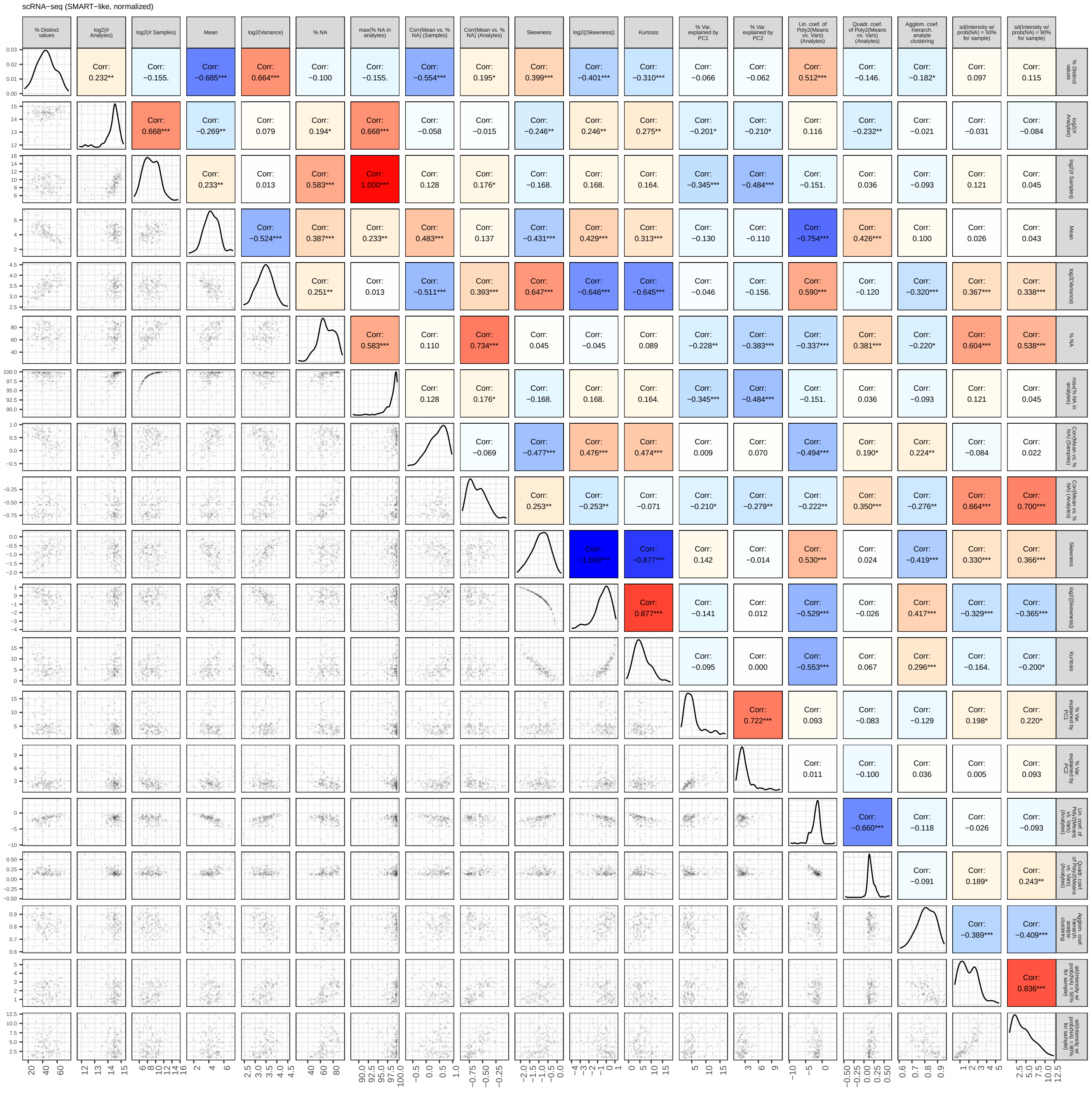

Figure S51. Spearman correlation plot of selected data characteristics across scRNA-seq (SMART-like, normalized) datasets. In the upper right corner the Spearman correlation coefficients for pairs of data characteristics are displayed, where *** = p<0.001, ** = p<0.01, * = p<0.05, and . = p<0.1. The intensity of red and blue corresponds to the strength of the positive and negative correlations, respectively. In the bottom left corner, scatter plots of pairs of data characteristics are shown, with each data point representing a dataset. On the diagonal, the distribution of each data characteristic is displayed.

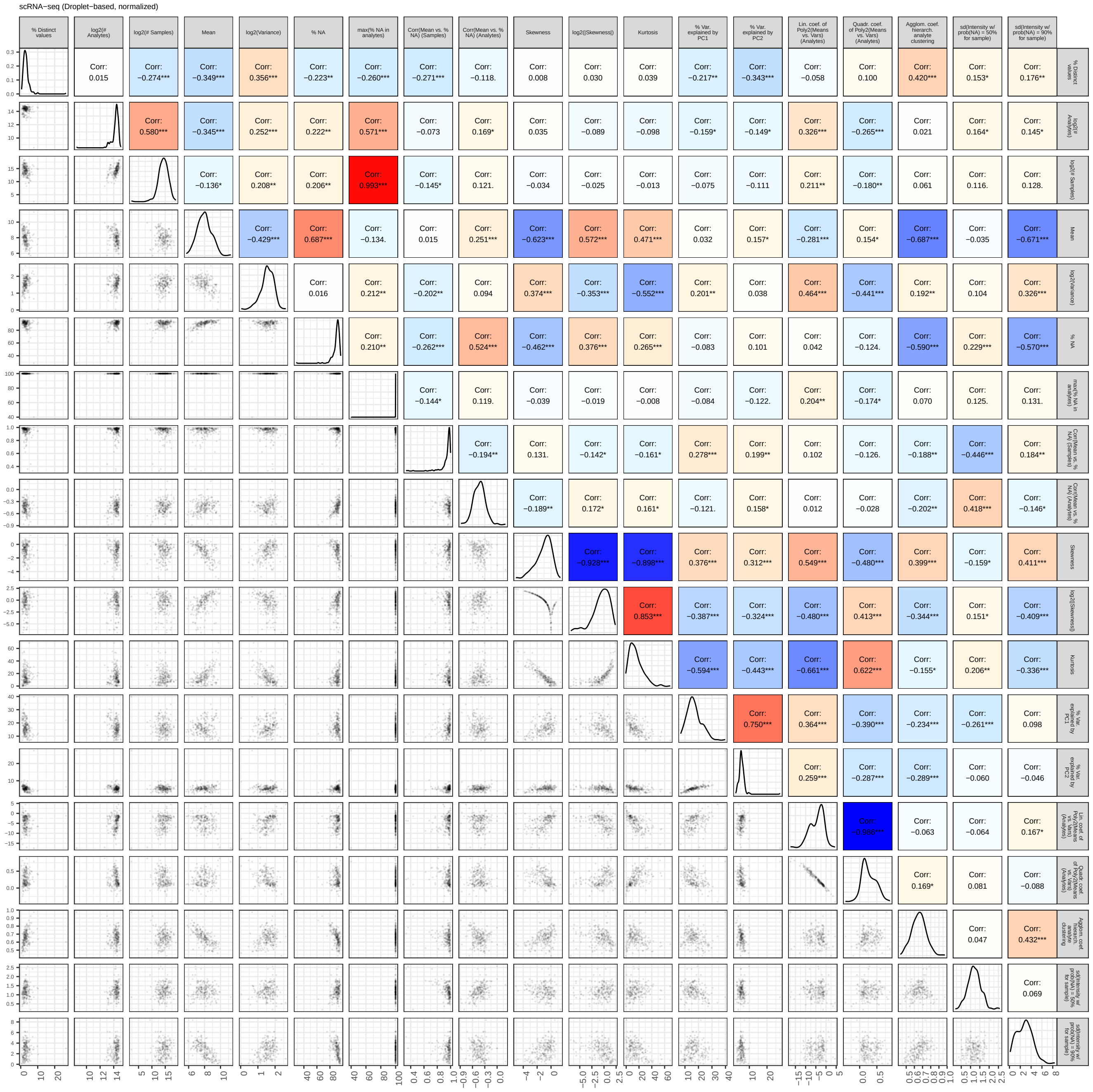

Figure S52. Spearman correlation plot of selected data characteristics across scRNA-seq (Droplet-based, normalized) datasets. In the upper right corner the Spearman correlation coefficients for pairs of data characteristics are displayed, where *** = p<0.001, ** = p<0.01, * = p<0.05, and . = p<0.1. The intensity of red and blue corresponds to the strength of the positive and negative correlations, respectively. In the bottom left corner, scatter plots of pairs of data characteristics are shown, with each data point representing a dataset. On the diagonal, the distribution of each data characteristic is displayed.

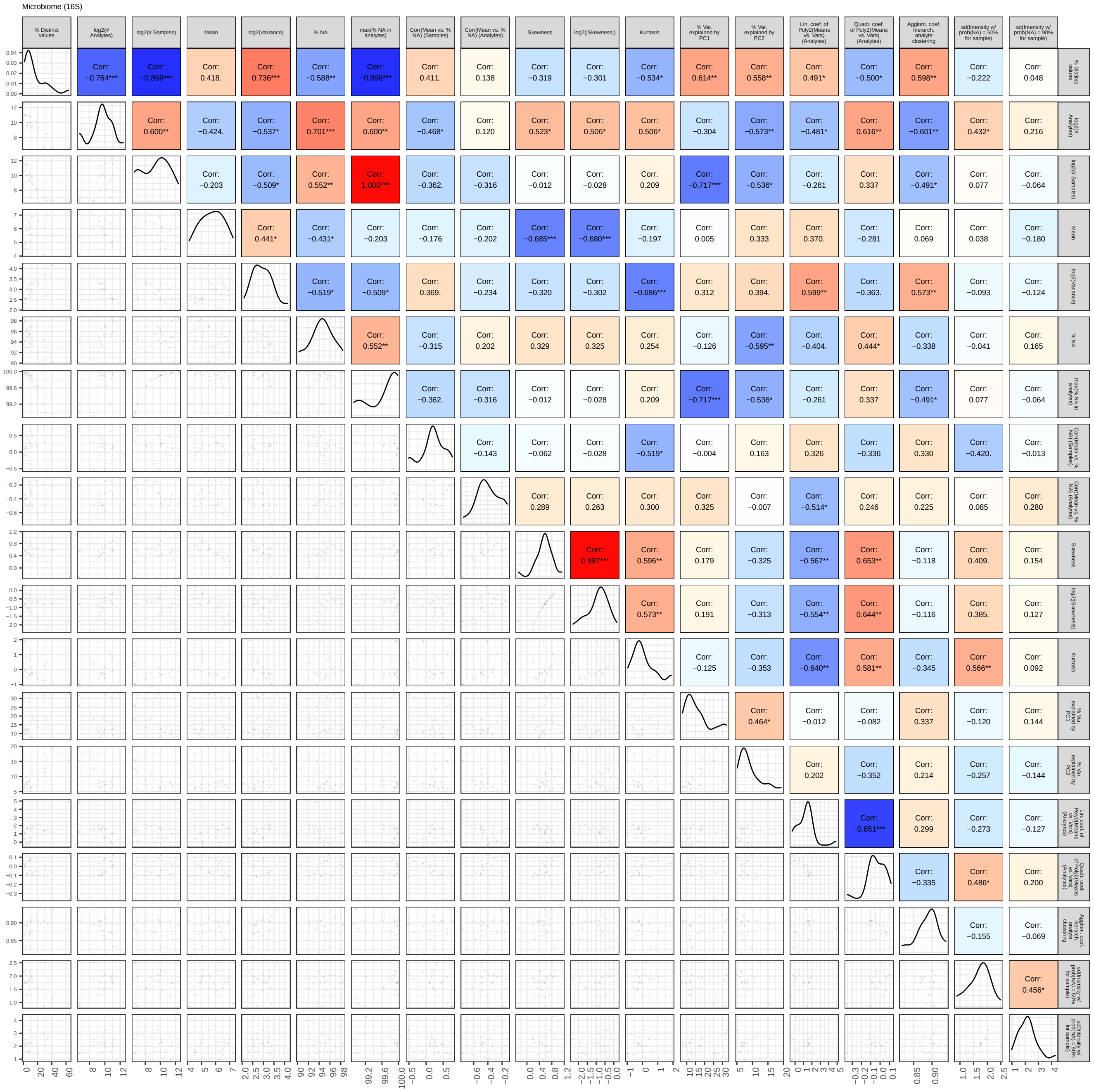

Figure S53. Spearman correlation plot of selected data characteristics across Microbiome (16S) datasets. In the upper right corner the Spearman correlation coefficients for pairs of data characteristics are displayed, where *** = p<0.001, ** = p<0.01, * = p<0.05, and . = p<0.1. The intensity of red and blue corresponds to the strength of the positive and negative correlations, respectively. In the bottom left corner, scatter plots of pairs of data characteristics are shown, with each data point representing a dataset. On the diagonal, the distribution of each data characteristic is displayed.

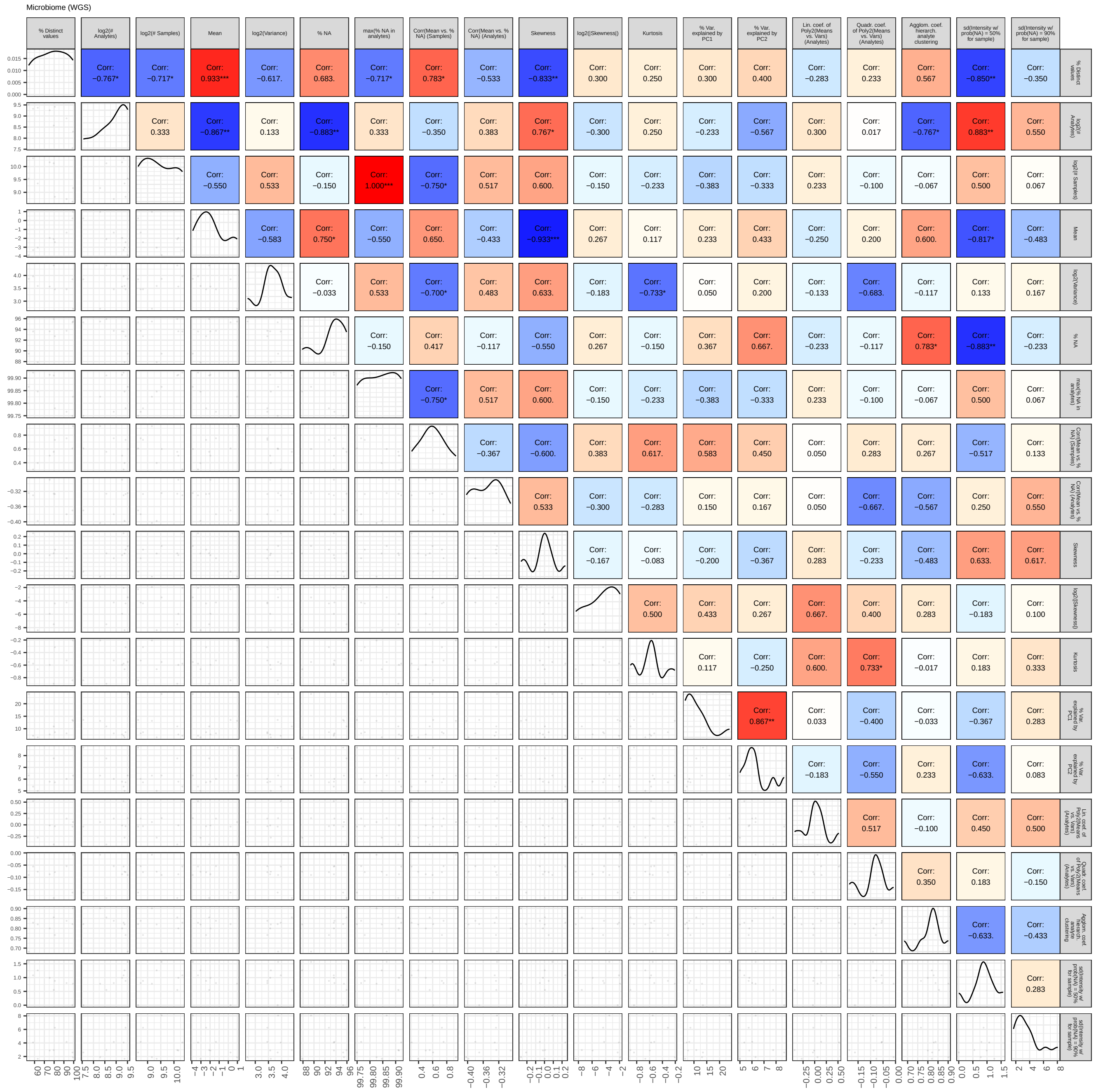

Figure S54. Spearman correlation plot of selected data characteristics across Microbiome (WGS) datasets. In the upper right corner the Spearman correlation coefficients for pairs of data characteristics are displayed, where *** = p<0.001, ** = p<0.01, * = p<0.05, and . = p<0.1. The intensity of red and blue corresponds to the strength of the positive and negative correlations, respectively. In the bottom left corner, scatter plots of pairs of data characteristics are shown, with each data point representing a dataset. On the diagonal, the distribution of each data characteristic is displayed.

(a)

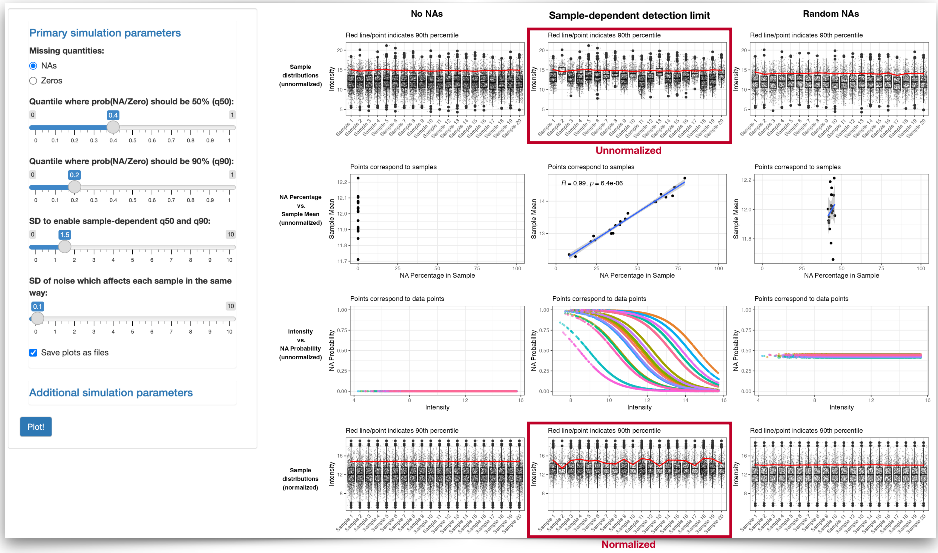

(b)

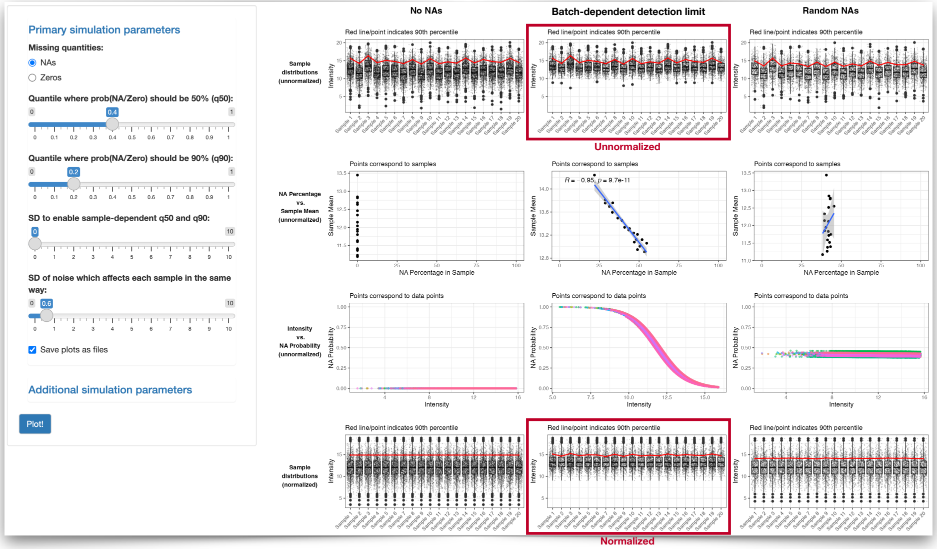

Figure S55. Simulation of datasets with (a) sample-dependent and (b) batch-dependent detection limits, accessible at `https://missingvaluesimulation.imbi.uni-freiburg.de`.

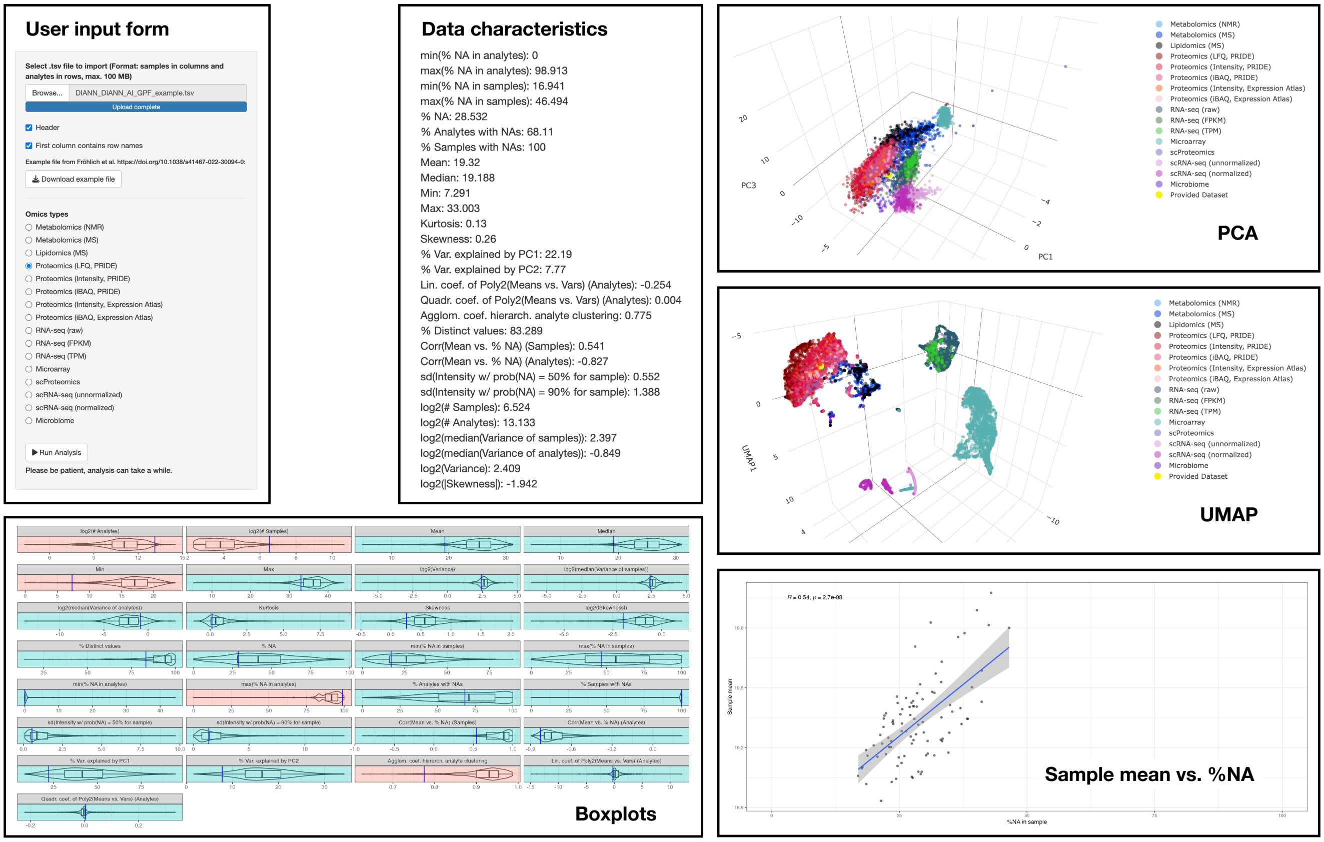

Figure S56. Components of the tool for assessing how representative a provided dataset is for a specific omics discipline based on its data characteristics, accessible at `https://omicscharacterization.imbi.uni-freiburg.de/`. Using the input form, users can upload a dataset to be assessed and specify the omics discipline the dataset should be compared to. The tool generates boxplots with a green background if the dataset falls between the 5th and 95th percentile, and a red background otherwise. Additionally, the data characteristics are displayed alongside principal component analysis (PCA) and uniform manifold approximation and projection (UMAP) plots, showing where the dataset fits within the clusters of different omics disciplines. Lastly, a plot of the sample mean versus the percentage of missing values is provided to assess potential correlations that suggest whether normalization should be performed.
